# Exploring Sesquiterpene Lactones from *Saussurea lappa*: Isolation, Structural Modifications, and Herbicide Bioassay Evaluation

**DOI:** 10.3390/plants14071111

**Published:** 2025-04-02

**Authors:** Elson S. Alvarenga, Francisco A. Macías, Stephani S. Ferreira, Juan C. G. Galindo, José M. G. Molinillo

**Affiliations:** 1 Department of Chemistry, Universidade Federal de Viçosa, Viçosa 36570-900, MG, Brazil; stephani.ferreira@ufv.br; 2 Department of Organic Chemistry, Faculty of Sciences, Universidad de Cádiz, c/República Saharui s/n, Puerto Real, 11510 Cádiz, Spain; famacias@uca.es (F.A.M.); juancarlos.galindo@uca.es (J.C.G.G.); chema.gonzalez@uca.es (J.M.G.M.)

**Keywords:** sesquiterpene lactone, herbicide, coleoptile, dehydrocostus lactone, DHC, epoxide

## Abstract

Considering the resistance of weeds to different herbicides with different mechanisms of action, the search for new, more selective compounds with low toxicity to other species in nature has been very important for the development of agriculture. Because of that, considering the biological activity of allelochemicals and natural epoxides, four new epoxy compounds derived from dehydrocostus lactone were synthetized and evaluated for their potential herbicide activity against three species of seeds, *Allium cepa* (onion), *Lepidium sativum* (garden cress), and *Lactuca sativa* (lettuce). In assays with *A. cepa*, compound **4** inhibited radicle length by 80% at 100 μM. Notably, for *L. sativum*, compound **4** showed significant inhibition, reducing stalk and radicle lengths by 80% at 100 μM, surpassing the performance of the commercial herbicide Logran. However, diol **5** notably inhibited radicle growth by 28% at 100 μM, making the most significant observed effect. One of the noteworthy lactones studied is epoxide **4**. This highlights the importance of the epoxide functional group in affecting both radicle and shoot lengths of seeds. Therefore, the synthesis of these compounds has proven advantageous and holds great potential for the development of new herbicides.

## 1. Introduction

The constant search for efficient herbicides is of the utmost importance for the agricultural industry. This is due to the presence of pests, which substantially affect global agricultural production, negatively interfering with the productivity of the crop, the final quality of the products, and consequently the production costs, mainly because of the relationship of competition of the culture with the plants for water, light, and nutrients [1,2].

Since the 20th century, synthetic herbicides have been used to control these unwanted plants; however, the constant and excessive use of herbs with the same mechanism of action (MoA) in large areas of cultivation, has resulted in the slow evolution and resistance of these pests against the exploited agrochemicals [1]. Thus, there is a growing demand for new, efficient herbicides with alternative mechanisms of action and low environmental impact [3,4].

In this context, natural products can be considered a viable option to replace traditional synthetic agrochemicals [5,6]. This is because they can be synthesized by living organisms, as well as produced through complex synthetic pathways that lead to molecules employing mechanisms of action different from those used by current agrochemicals [7]. Furthermore, herbicides like phosphinothricin and phosalacin, derived from natural products, underscore the feasibility of studying and evaluating these compounds as potential future herbicides [8].

The sesquiterpenic lactones, one of the largest families of natural products, are compounds widely distributed in plants and more than 7000 structures of this class have already been described [9,10]. In addition, studies referring to these compounds indicate that they have a broad biological potential, standing out in terms of cytotoxic, antitumoral, antibacterial, anti-inflammatory, antimalaric, and antifungal activities [11,12,13,14].

These natural compounds can be isolated mainly from the aerial parts of certain plants of the *Asteraceae* family, also being found in families such as *Umbelliferae*, *Lauraceae,* or *Magnoliaceae* [7]. Sesquiterpenes also represent an important group of secondary metabolites of the *Asteraceae* family [15,16,17].

Sesquiterpenic lactones also have great potential for application in the agricultural sector [16,18]. Studies show that some secondary metabolites can affect the germination and growth of certain plant species, but the nature and the extent of the effects produced depend on several factors, such as the structure of the lactone tested, its concentration, and the species on which it will act [19,20,21].

**Figure 1:**
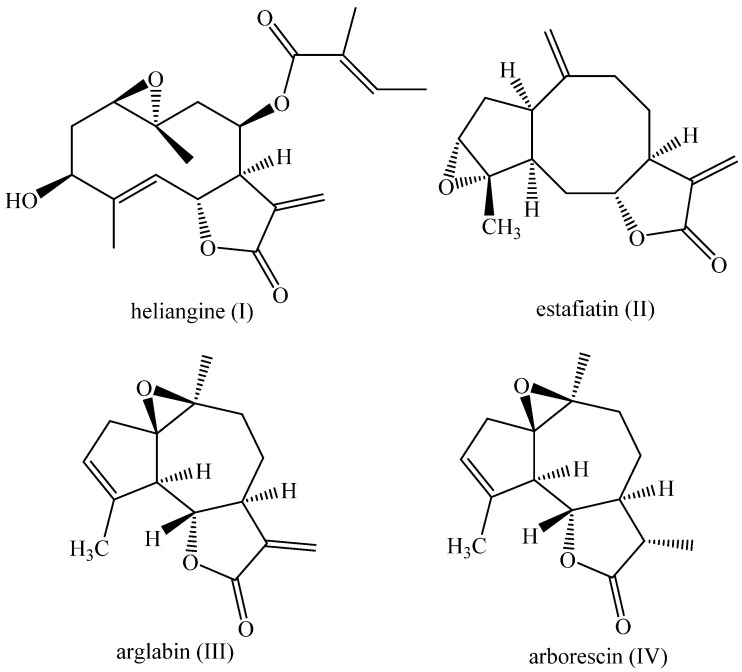
Sesquiterpene lactones: heliangine (**I**), estafiatin (**II**), arglabin (**III**), and arborescin (**IV**).

This effect, called allelopathy, is of great importance in agriculture for pest control, provided that a correct association is established between the species producing allelopatic substances and the cultivated species [22,23]. Sesquiterpene lactones such as estafiatin, arglabin, arborescin, and heliangine serve as examples of compounds with allelopathic effects, producing chemicals that negatively impact the growth and development of competing plants [20] (Figure 1).

The compounds arglabin and arborescin, guaianolides structurally related to estafiatin [24], are isolated natural epoxides of *Artemisia glabella* and *Artemissa arborescens*, respectively [25,26]. The stereoselective syntheses of these natural products are described in the literature [25,26]. As for the compound heliangine, it is an epoxide of a germacranolide with plant physiological activity, isolated from *Helinthus tuberosus* L., which can act as a regulator of plant growth [27,28].

Studies suggest that the α-methylene-butirolactone group, present in many natural sesquiterpene lactones (SLs), plays an important role in the bioactivity of these compounds [29,30]. This group has been proposed as one of the structural aspects that can determine their allelopathic activity, as well as their biological activity in general [29,30,31].

The activity of SLs is linked to their α-methylene-butirolactone component, which serves as a potent and selective alkylating agent for nucleophilic substrates. In fact, the primary determinant of SL cytotoxicity is the presence of an α,β-unsaturated keto group, which is not necessarily associated with the lactone’s carbonyl group [7,32]. Additionally, another study found that the absence of this system does not significantly diminish the growth of weeds [7]. The spatial arrangement of the carbon skeleton has been linked to the lower activity of sesquiterpene lactones. The eudesmanolides reynosin and santamarin feature a backbone that closely resembles the spatial arrangement found in germacranolides and also possess an α,β-unsaturated carbonyl system. Compounds with a “double crown”-like spatial configuration (such as germacranolides and eudesmanolides) resemble strigolactones and can fit into the receptor’s cavity. However, the observed activity of compounds lacking the unsaturated double bond in the lactone ring remains unexplained. Further research exploring precise biochemical pathways is needed, reinforcing its potential as a sustainable resource for enhancing herbicidal activity and well-being [33,34,35,36].

Thus, given the well-documented biological activity of allelochemicals—natural compounds that influence the growth and development of other organisms—and natural epoxides, which are known for their diverse bioactive properties, our research is directed toward the synthesis of four novel epoxy sesquiterpene lactones and other derivatives. These derivatives are designed from dehydrocostus lactone, a sesquiterpene lactone that has already been recognized for exhibiting multiple biological activities [37,38]. The primary objective of this study is to evaluate the potential herbicidal activity of these newly synthesized compounds, assessing their effectiveness in inhibiting plant growth and exploring their possible application as natural herbicides in agricultural weed management.

## 2. Results and Discussion

### 2.1. Synthesis

**Figure 2:**
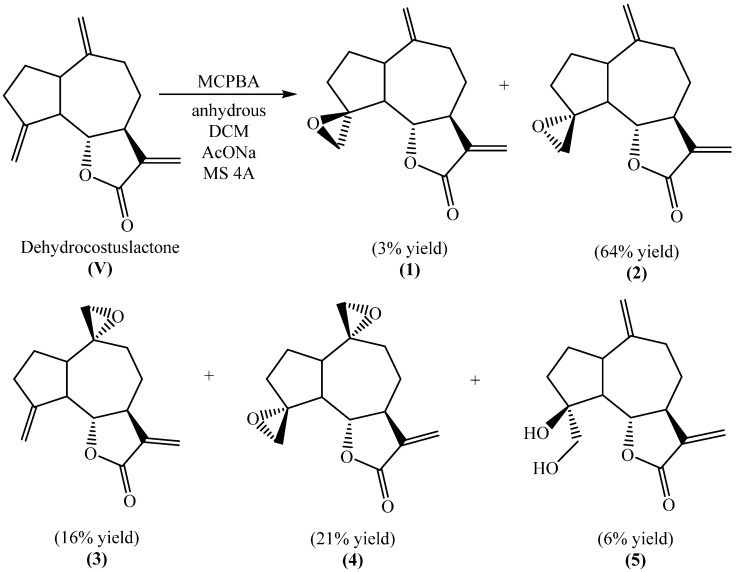
Epoxidation reaction of dehydrocostus lactone (**V**).

For the study of the herbicidal activity of sesquiterpene lactones, new epoxy derivatives were synthesized from dehydrocostus lactone (DHC, **V**), yielding derivatives (**1**), (**2**), (**3**), (**4**), and (**5**). These compounds were obtained from the reaction of DHC with *meta*-chloroperbenzoic acid (MCPBA) in anhydrous DCM, with yields of 3%, 64%, 16%, 21%, and 6%, respectively, as shown in Figure 2.

As expected, compounds **1**–**3** exhibited similar spectrometric data, as they differ only in the positions and relative stereochemistry of the epoxide moieties and hydroxyl groups attached to the new components. The IR spectra display peaks in the range of 1765–1770 cm^−1^, indicating the presence of lactone carbonyl groups, and a band near 1260 cm^−1^, attributed to C–O stretching. Additionally, compound **5** shows a peak at 3472 cm^−1^, indicating the presence of a hydroxyl group [39].

The ^1^H NMR and ^13^C NMR spectra were crucial for the identification of the compounds, providing detailed information about their molecular structure, including the chemical environment of hydrogen and carbon atoms. These spectra allowed for the accurate determination of functional groups, the number of protons and carbons, as well as their connectivity within the molecule.

In the ^13^C NMR spectra, signals between *δ* = 168.0 and 169.5 ppm observed in all five spectra correspond to a carbonyl group. Signals from sp^2^-hybridized carbon and carbon bonded to electronegative atoms, such as those in epoxides and hydroxyl-bonded carbons in molecule **5**, are exhibited in the deshielded regions of the spectrum.

In the ^1^H NMR spectra, the signals corresponding to hydrogens bonded to sp^2^ carbons are found in the deshielded regions of the spectrum due to the anisotropic effect occurring in double bonds. An example is the signal of hydrogen H13 at δ = 6.06–6.07 ppm in molecule 1, appearing as a doublet of doublets (*J* = 0.4, 2.8 Hz), as shown in Figure S9 in the Supplementary Information (SI). This technique also aids in the analysis of hydrogen bonded to sp^3^ carbons, resulting in signals in more shielded regions of the spectrum, such as the signal of hydrogen H8 at δ = 1.29–1.36 ppm in molecule 2. This signal, integrated for one hydrogen, appears as a multiplet in Figure S13 (SI).

The analyses of COSY (correlation spectroscopy), HMBC (heteronuclear multiple bond coherence), and HMQC (heteronuclear multiple quantum correlation) were also equally important for the complete assignment of the signals [40].

To determine the relative configuration of the molecules by NMR spectroscopy, the spatial distances between the hydrogen atoms of the molecules were measured using Overhauser nuclear effect (NOE) experiments. These experiments provide crucial information about through-space interactions between nuclei, allowing for the identification of proximity relationships within the molecular structure. By analyzing NOE enhancements, it is possible to infer the three-dimensional arrangement of atoms, which aids in distinguishing between different stereochemical configurations. This approach is particularly useful for rigid molecules in solution, where direct bond connectivity alone may not be sufficient to establish the full spatial arrangement [41].

**Figure 3:**
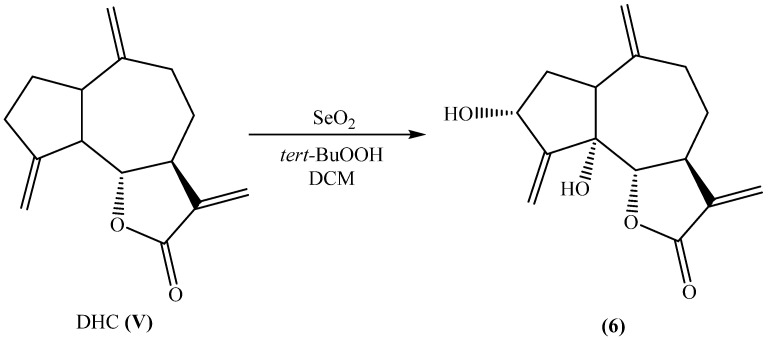
Dihydroxilation of dehydrocostuslactone.

As previously described, compound 5 was also utilized in the synthesis of 5α-hydroxy-isozaluzanine C (**6**) through a dihydroxylation reaction. This transformation was carried out using DHC as the starting material, with selenium oxide and *tert*-butyl hydroperoxide serving as the key oxidizing agents. The reaction conditions were carefully controlled to promote selective oxidation, leading to the formation of the hydroxylated product. This method provides an efficient approach to modifying the molecular structure, potentially enhancing its biological activity. The reaction pathway and structural changes involved in this transformation are illustrated in Figure 3.

**Figure 4:**
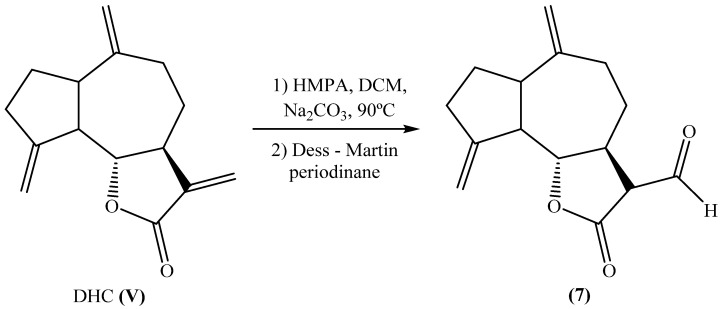
Michael addition of a nucleophilic hydroxyl group to DHC and Dess–Martin periodinane oxidation.

The formation of 13-oxo-epi-costuslactone (**7**) involved a two-step synthetic process starting with the reaction of DHC with aqueous sodium carbonate in hexamethylphosphoramide (HMPA). Following this transformation, the primary alcohol underwent oxidation using Dess–Martin periodinane, a mild and selective oxidizing agent commonly employed for converting primary alcohols to aldehydes. This oxidation step was crucial for achieving the desired structural modification, ultimately leading to the formation of 13-oxo-epi-costuslactone (Figure 4).

### 2.2. Coleoptile Bioactivity

The study of coleoptile bioactivity provides valuable information about the mechanisms of action of bioactive substances and their potential agricultural applications. The wheat coleoptile bioassay is used to evaluate plant growth stimulation and assess the potential of natural products, synthetic compounds, or agricultural chemicals as herbicides.

Tests using etiolated wheat coleoptiles have been employed to assess the herbicidal potential of compounds prepared by microwave irradiation of costunolide [42], extracts from the leaves of *Origanum majorana* L. [43], steroidal saponins [44], furanocoumarins isolated from the aerial parts of *Ducrosia anethifolia* [45], and many other sources.

Seven compounds were obtained from DHC and subjected to a bioassay using etiolated wheat coleoptiles. Six dilutions (1000, 600, 300, 100, 30, and 10 μmol L^−1^) were used in the assay. The etiolated wheat coleoptile bioassay was chosen as a preliminary method to evaluate the bioactivity of compounds **1**–**7** due to its sensitivity to a broad spectrum of bioactive substances.

**Figure 5:**
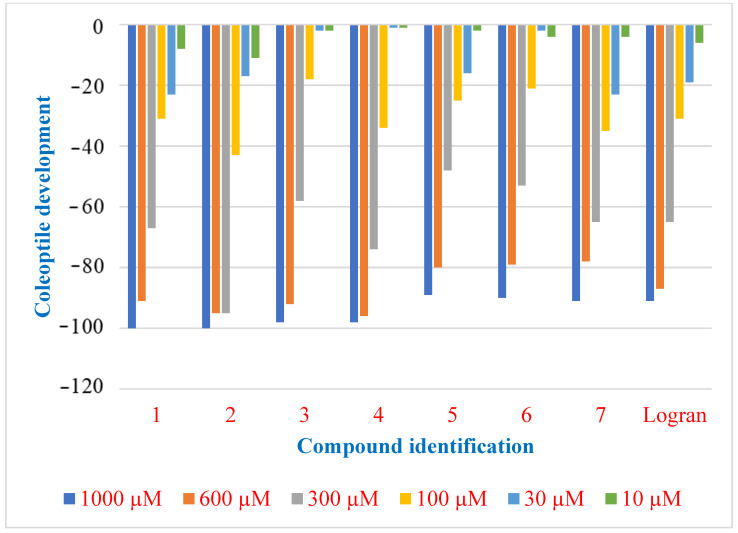
Bioactivity evaluation of compounds **1**–**7** and Logran on coleoptile development.

The results shown in Figure 5 indicate that all compounds exhibited high inhibitory activity at a concentration of 1000 μM, with compounds **1** and **2** standing out. A decrease in inhibitory activity was observed as the concentration was reduced. Notably, even at lower concentrations, compounds **1** and **2** remained the most active, confirming that compounds **1** and **2** were the most effective in the coleoptile bioassay.

### 2.3. Herbicide Activity

**Figure 6:**
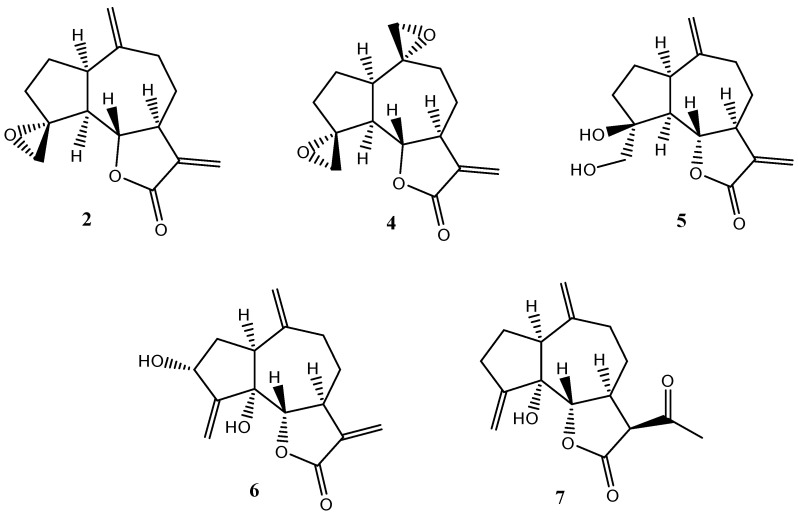
Compounds employed in the seed’s bioassay.

In this study, the efficacy of compounds **2**, **4**, **5**, **6**, and **7** (Figure 6) in stimulating or inhibiting root and shoot development was evaluated using three plant species: *Allium cepa* (onion), *Lepidium sativum* (garden cress), and *Lactuca sativa* (lettuce). These species were selected due to their well-documented sensitivity to growth-regulating compounds, making them suitable bioindicators for assessing potential stimulatory or inhibitory effects.

The experimental process involved exposing seeds of each species to different concentrations of the selected compounds under controlled conditions. After the germination period, the seedlings were carefully analyzed and measured to determine variations in growth parameters. The collected data were then presented in graphical form, illustrating percentage differences compared to the control group.

In this analysis, the value of zero corresponds to the control, indicating no change in growth under water treatment. Positive percentage values denote stimulation, reflecting an increase in root or shoot development, while negative percentage values indicate inhibition, representing a reduction in growth compared to the control. These results provide insights into the bioactivity of the tested compounds, contributing to a better understanding of their potential applications in plant growth regulation [46,47].

**Figure 7:**
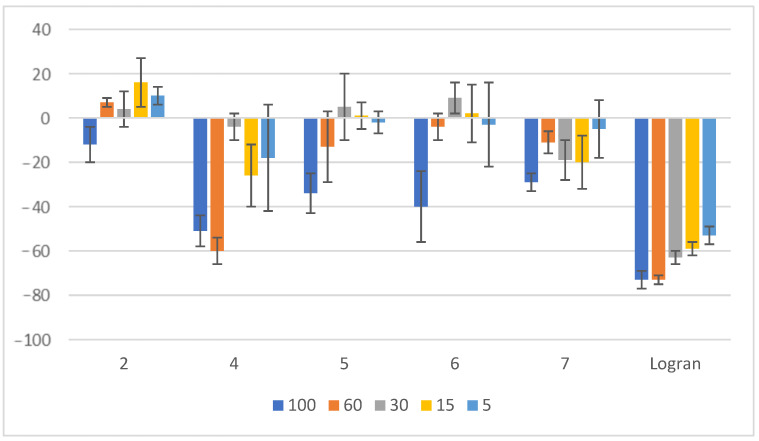
Effect of compounds **2** and **4**–**7** on the shoot length of *A. cepa*. Shoot length of seedlings from onion seeds exposed to aqueous 0.1% (*v/v*) DMSO solutions of compounds at different concentrations. Controls consisted of deionized water with the same concentration of DMSO. Values are expressed as percentage difference from the negative control, calculated as: shoot length (%) = [(length − length of negative control)/length of negative control] × 100. Error bars represent the standard deviation.

As illustrated in Figure 7, compounds **4** and **6** demonstrated the most significant inhibitory effects on the shoot length of *A. cepa* when compared to the other tested compounds. The data indicate that compound **4** exhibits inhibitory effect, achieving 51% inhibition at a concentration of 100 μM and an even higher 60% inhibition at 60 μM, suggesting a potent dose-dependent response. Similarly, compound **6** also displayed notable activity, with an inhibition percentage of 40% at 100 μM. These findings highlight the promising potential of these compounds as effective plant growth inhibitors, reinforcing their role in herbicidal applications.

**Figure 8:**
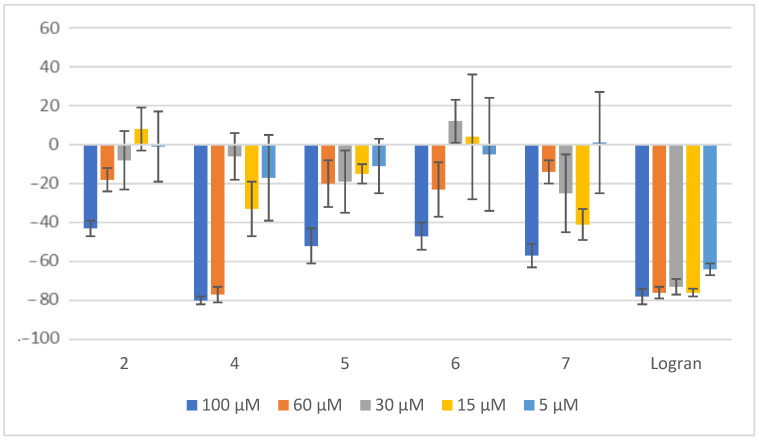
Effect of compounds **2** and **4**–**7** on the radicle length of *A. cepa*. Radicle length of seedlings from onion seeds exposed to aqueous 0.1% (*v/v*) DMSO solutions of compounds at different concentrations. Controls consisted of deionized water with the same concentration of DMSO. Values are expressed as percentage difference from the negative control, calculated as: shoot length (%) = [(length − length of negative control)/length of negative control] × 100. Error bars represent the standard deviation.

Regarding the inhibition of radicle length in *A. cepa*, compound **4** stands out as the most effective among all the tested compounds, exhibiting an impressive 80% inhibition at 100 μM and 77% inhibition at 60 μM (Figure 8). This indicates a strong inhibitory effect even at a slightly lower concentration. Furthermore, all other tested compounds also demonstrated notable inhibitory activity, with inhibition percentages exceeding 40% at 100 μM. These results emphasize the high efficacy of these compounds, particularly at a concentration of 100 μM, in suppressing root elongation.

**Figure 9:**
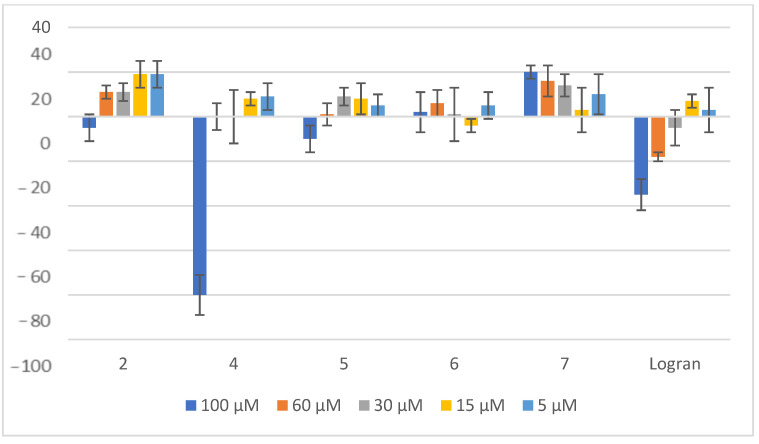
Effect of compounds **2** and **4**–**7** on the stalk length of *L. sativum*. Shoot length of seedlings of garden cress seeds exposed to aqueous 0.1% (*v/v*) DMSO solutions of the tested compounds at different concentrations. Controls consisted of deionized water with the same concentration of DMSO. Values are expressed as percentage difference from the negative control, calculated as: shoot length (%) = [(length − length of negative control)/length of negative control] × 100. Error bars represent the standard deviation.

Compound **4** once again demonstrates remarkable biological activity, this time in the inhibition of *L. sativum* stalk length (Figure 9). At a concentration of 100 μM, this compound exhibits an impressive inhibition rate of 80%, which is twice as effective as the commercially available herbicide Logran, used as a reference compound in this study. This significant difference highlights the potential of compound **4** as a potent growth inhibitor.

**Figure 10:**
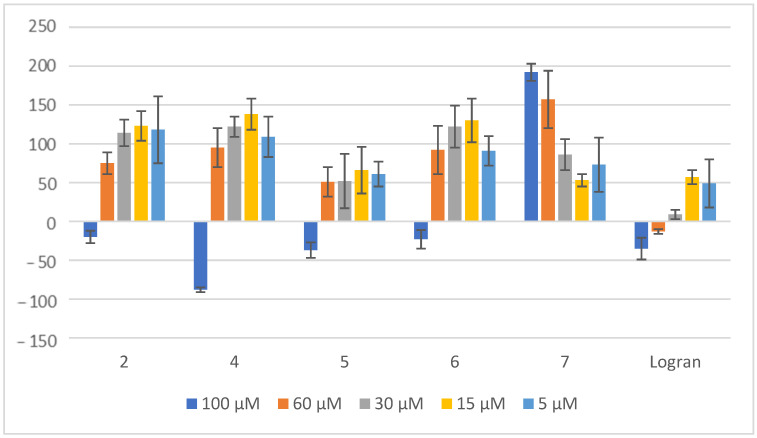
Effect of compounds **2** and **4**–**7** on the radicle length of *L. sativum*. Radicle length of seedlings from garden cress seeds exposed to aqueous 0.1% (*v/v*) DMSO solutions of compounds at different concentrations. Controls consisted of deionized water with the same concentration of DMSO. Values are expressed as percentage difference from the negative control, calculated as: shoot length (%) = [(length − length of negative control)/length of negative control] × 100. Error bars represent the standard deviation.

As illustrated in Figure 10, all the tested compounds demonstrated a stimulatory effect on the growth of the radicle of garden cress. This suggests that, at certain concentrations, these compounds may play a role in promoting root elongation and overall seedling development. However, an exception was observed for compound **4**, which, at a concentration of 100 μM, significantly inhibited the root growth of *L. sativum* by 80%. This indicates a strong suppressive effect at this specific concentration, potentially interfering with root elongation and development. Such findings highlight the varying influence of different compounds on plant growth, depending on their concentration and the species under study. 

**Figure 11:**
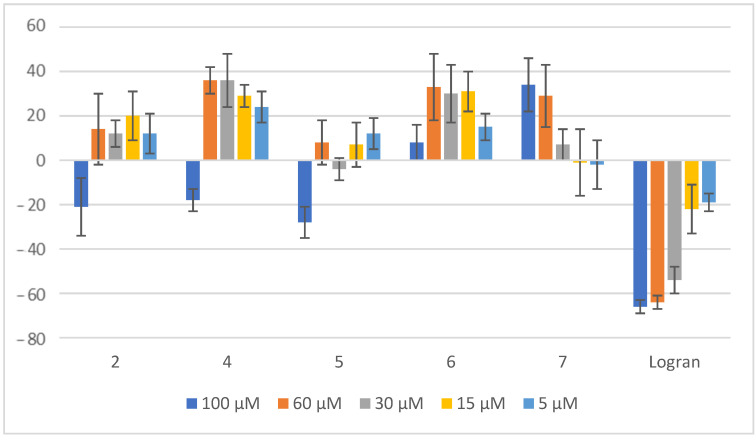
Effect of compounds **2** and **4**–**7** on the radicle length of *L. sativa*. Radicle length of seedlings from lettuce seeds exposed to aqueous 0.1% (*v/v*) DMSO solutions of compounds at different concentrations. Controls consisted of deionized water with the same concentration of DMSO. Values are expressed as percentage difference from the negative control, calculated as: shoot length (%) = [(length − length of negative control)/length of negative control] × 100. Error bars represent the standard deviation.

No significant changes were observed for the compounds tested when compared to the length of the stem of *L. sativa*. However, upon further analysis, as illustrated in Figure 11, it was verified that compound **5** had a notable inhibitory effect on radicle growth. Specifically, at a concentration of 100 μM, compound **5** reduced radicle length by 28%, making it the most significant change observed among all tested compounds. This suggests that compound **5** exhibits a measurable impact on early seedling development, potentially affecting root elongation more than stem growth. Compounds **4**, **6**, and **7** presented stimulatory effect on the growth of lettuce. These three compounds stimulated almost 40% the growth of the radicle of *L. sativa*. 

## 3. Materials and Methods

**General experimental procedures.** The *Saussurea lappa* extract (100 g) was subjected to chromatographic separation using silica gel column chromatography. The column used for this process measured 30 cm in height and 10 cm in diameter. The separation was carried out using a mobile phase consisting of hexane and ethyl acetate in a 95:5 (*v/v*) ratio, ensuring effective elution of the desired compounds. After fractionation and purification, pure dehydrocostus lactone (DHC) (5.0 g) was successfully isolated. The structural identity and purity of the isolated DHC were confirmed using various spectroscopic techniques, including ^1^H and ^13^C nuclear magnetic resonance (NMR) spectroscopy, as well as two-dimensional (2D) high-resolution NMR analyses (Table 1). The detailed spectral data supporting this identification are provided in Figures S2–S7 in the Supplementary Information (SI).

Epoxide derivatives—(3a*S*,9*S*,9b*S*)-3,6-dimethylenedecahydro-2*H*-spiro[azuleno[4,5-b]furan-9,2′-oxiran]-2-one **(1)**, (3a*S*,9*R*,9b*S*)-3-methylenedecahydro-2*H*-spiro[azuleno[4,5-b]furan-9,2′-oxiran]-2-one **(2)**, (3a*S*,6*R*,9b*S*)-3,9-dimethylenedecahydro-2*H*-spiro[azuleno[4,5-b]furan-6,2′-oxiran]-2-one **(3)**, (2*R*,3a’*S*,9′*R*,9b’*S*)-3′-methyleneoctahydrodispiro[oxirane-2,6′-azuleno[4,5-b]furan-9′,2″-oxiran]-2′(3′*H*)-one **(4)** and the diol (3a*S*,9*S*,9b*S*)-9-hydroxy-9-(hydroxymethyl)-6-methylenedecahydroazuleno[4,5-b]furan-2(3*H*)-one **(5)**—were prepared by epoxidation reaction of DHC with *meta*-chloroperbenzoic acid. All the structures were confirmed by high resolution NMR spectroscopy (the compounds were separated by HPLC, analytic column, refractive index detector, and characterized by ^1^H and ^13^C NMR, n.O.e experiments, HSQC and HMBC).

### 3.1. Synthetic Procedures

Epoxidation of DHC. *meta*-chloroperbenzoic acid (0.79 g, MW 172.57 g/mol, 4.58 mmol) in 15 mL anhydrous dichloromethane (DCM) was added to an ice cooled suspension of DHC (0.50 g, MW 230 g/mol, 2.17 mmol), anhydrous sodium acetate (0.46 g, MW 82.03 g/mol, 5.61 mmol), and powder molecular sieves (2 g) in anhydrous DCM (10 mL). The ice bath was removed, and the reaction mixture was stirred for 3 h. The mixture was fractioned through a neutral alumina column and eluted with DCM (100 mL) and a mixture of hexane–ethyl acetate 20% (500 mL). The fractions were combined and concentrated under reduced pressure to afford clear yellow oil (0.270 g). The oily residue (4 mg) was dissolved in hexane–ethyl acetate 60% (0.2 mL) and injected in the HPLC with semi-preparative column eluting with hexane–ethyl acetate 60%. The reaction yield was calculated employing the chromatogram obtained.

*(3aS,9S,9bS)-3,6-dimethylenedecahydro-2H-spiro[azuleno[4,5-b] furan-9,2′-oxiran]-2-one* (**1**)

[*α*]_D_ = 10.1 (methanol). IR $\bar{v}$ cm^−1^: 2931, 1768, 1638, 1259, 1140, 999. ^1^H NMR (400 MHz, C_6_D_6_): *δ* 6.06 (dd, *J* = 0.4, 2.8 Hz, H13), 4.81 (dd, *J* = 0.4, 2.8 Hz, H13′), 4.64 (d, *J* = 4.8 Hz, H14), 4.61 (d, *J* = 4.8 Hz, H14′), 3.31 (d, *J* = 4.8 Hz, H15), 3.23 (dd, *J* = 8.8, 11.0 Hz, H6), 2.76 (q, *J* = 8 Hz, H1), 2.57 (d, *J* = 4.8 Hz, H15′), 1.71–1.94 (m, H2, H3, H5, H7, H9), 1.38–1.56 (m, H2′, H3′, H8, H9′), 0.67–0.77 (m, H8′). ^13^C NMR (100 MHz, C_6_D_6_): *δ* 28.7 (C2), 29.6 (C8), 31.7 (C3), 33.1 (C9), 45.5 (C7), 47.02 (C1), 49.6 (C15), 53.4 (C5), 65.8 (C4), 81.0 (C6), 113.5 (C14), 118.6 (C13), 140.5 (C10), 148.6 (C11), 168.7 (C=O).

*(3aS,9R,9bS)-3-methylenedecahydro-2H-spiro[azuleno[4,5-b] furan-9,2′-oxiran]-2-one* (**2**)

[*α*]_D_ = −20.0 (methanol). IR $\bar{v}$ cm^−1^: 2930, 1767, 1635, 1260, 1130, 995. ^1^H NMR (400 MHz, C_6_D_6_): *δ* 6.03 (d, *J* = 3.2 Hz, H13), 4.88 (m, H14), 4.82 (d, *J* = 3.2 Hz, H13′), 4.74 (m, H14′), 3.80–3.83 (m, H6, d, *J* =12 Hz, H15), 3.31 (d, *J* =12 Hz, H15′), 2.22–2.29 (m, H1), 2.12 (dt, *J*= 4, 12 Hz, H7), 1.87–1.96 (m, H5, H3′), 1.63–1.77 (m, H2′, H3), 1.40–1.54 (m, H2, H9, H9′), 1.29–1.36 (m, H8), 0.60–0.77 (m, H8′). ^13^C NMR: (100 MHz, C_6_D_6_): *δ* 24.4 (C8), 26.7 (C2), 32.4 (C3), 35.5 (C9), 44.3 (C7), 45.4 (C1), 49.4 (C15), 50.3 (C5), 57.1 (C4), 83.4 (C6), 108.9 (C14), 118.9 (C13), 140.4 (C10), 151.4 (C11), 168.9 (C=O).

*(3aS,6R,9bS)-3,9-dimethylenedecahydro-2H-spiro[azuleno[4,5-b]furan-6,2′-oxiran]-2-one* (**3**)

[*α*]_D_ = −7.80 (methanol). IR $\bar{v}$ cm^−1^: 2933, 1765, 1257, 1149, 999. ^1^H NMR (400 MHz, C_6_D_6_): *δ* 5.32–5.33 (m, H15), 4.92–4.95 (m, H15′), 4.84 (d, *J* = 2.8 Hz, H13′), 3.41 (dd, *J* = 8, 10 Hz, H6), 2.32–2.37 (m, H5), 2.00–2.15 (m, H7, H3, H3′, H14), 1.88–1.96 (m, H1, H14′), 1.27–1.53 (m, H2, H2′, H8, H9), 1.12–1.18 (m, H9′), 0.59–0.69 (m, H8′). ^13^C NMR (100 MHz, C_6_D_6_): *δ* 24.5 (C2), 26.6 (C8), 32.3 (C3), 35.4 (C9), 44.2 (C7), 45.3 (C1), 49.3 (C14), 50.5 (C5), 57.1 (C10), 83.4 (C6), 108.8 (C15), 119.1 (C13), 140.3 (C11), 151.4 (C4), 169.1 (C=O).

*(2R,3a’S,9′R,9b’S)-3′-methyleneoctahydrodispiro[oxirane-2,6′-azuleno[4,5-b]furan-9′,2′-oxiran]-2′(3′H)-one* (**4**)

[*α*]_D_ = 15.5 (methanol). IR $\bar{v}$ cm^−1^: 2935, 1767, 1256, 1130, 999. ^1^H NMR (400 MHz, C_6_D_6_): *δ* 6.10 (d, *J* = 2.8 Hz, H13), 4.82 (d, *J* = 2.8 Hz, H13’), 3.63 (dd, *J* = 9.2, 10 Hz, H6), 3.12 (d, *J* = 4.4 Hz, H15’), 2.71 (dd, *J* = 2, 4.4 Hz, H14), 2.35 (d, *J* = 4.8 Hz, H15’), 2.06–2.17 (m, H5, H14’, H15’), 1.86–1.91 (m, H1, H7), 1.54–1.65 (m, H2, H3), 1.36–1.49 (m, H2’, H9), 1.21–1.30 (m, H8), 1.12–1.18 (m, H3’, H9’), 0.50–0.61 (m, H8’). ^13^C NMR (100 MHz, C_6_D_6_): *δ* 22.4 (C2), 27.1 (C8), 32.7 (C3), 38.6 (C9), 43.6 (C5), 44.1 (C7), 47.3 (C14), 47.4 (C1), 48.4 (C15), 56.2 (C10), 64.6 (C4), 80.4 (C6), 119.3 (C13), 139.5 (C11), 168.9 (C=O).

*(3aS,9S,9bS)-9-hydroxy-9-(hydroxymethyl)-6-methylenedecahydroazuleno[4,5-b]furan-2(3H)-one* (**5**)

[*α*]_D_ = 19.2 (methanol). IR $\bar{v}$ cm^−1^: 3472, 2937, 1770, 1257, 1131, 996. ^1^H NMR (400 MHz, C_6_D_6_): *δ* 4.07 (d, *J* = 3.6 Hz, H13), 4.89–4.90 (m, H14), 4.83 (d, *J* = 3.6 Hz, H13′), 4.77–4.78 (m, H14′), 3.88 (t, *J* = 9.6 Hz, H6), 3.01 (d, *J* = 4.8 Hz, H15), 2.36 (d, *J* = 4.8 Hz, H15’), 2.22–2.27 (m, H1), 1.96 (t, *J* = 9.2 Hz, H5), 1.74–1.85 (m, H2, H3, H9), 1.42–1.66 (m, H2′, H3′, H8, H9′), 0.71–0.81 (m, H8′). ^13^C NMR (100 MHz, C_6_D_6_): *δ* 29.4 (C2), 31.0 (C8), 33.4 (C3), 38.5 (C9), 44.3 (C7), 46.3 (C1), 47.6 (C15), 49.0 (C5), 64.3 (C4), 81.0 (C6), 112.5 (C13), 118.9 (C14), 139.9 (C11), 149.3 (C10), 169.1 (C=O).

*Synthesis of 5α-hydroxy-isozaluzanine C* (**11**). Selenium oxide (2 eq. molar) and *tert*-butylhydroperoxide (2 eq. molar) were added to a solution of DHC (0.50 g, MW 230 g/mol, 2.17 mmol) in anhydrous DCM (50 ML) and stirred for 4 h under nitrogen atmosphere. Hydroxy-isozaluzanine C was fractioned in a column of silica gel and purified by HPLC (semi-preparative column; hexane–ethyl acetate 8:2) in 35% yield according to the previously reported conditions [48].

*Synthesis of 13-oxo-epi-costuslactone.* Aqueous sodium carbonate (20%) was added dropwise to a solution of DHC (0.200 g) in HMPA (20 mL) until it became turbid (around 16 mL). The reaction mixture was magnetically stirred and heated at 90 °C for 6 days. The mixture was extracted with ethyl acetate (4 × 10 mL) and the combined organic phases were washed with 2M aqueous hydrochloric acid (3 × 10 mL), 20% aqueous sodium carbonate (2 × 10 mL), and brine (2 × 10 mL). The organic phase was dried with anhydrous sodium sulphate, filtered, and concentrated under reduced pressure. The mixture of 13-hydroxycostuslactone and 13-hydroxy-epi-costuslactone was oxidized to the corresponding aldehyde using Dess–Martin periodinane according to the previously reported conditions [49].

### 3.2. Coleoptile Bioassay

Wheat seeds (*Triticum aestivum*) were sown in 15 cm diameter Petri dishes lined with moist filter paper and incubated in darkness at 25 °C for three days [50]. Coleoptiles measuring 25–35 mm in length were selected under a green safelight. A 3 mm section from the tip was removed and discarded, while the next 4 mm segment was used for the bioassay. After cutting, coleoptiles were soaked in distilled water for one hour before being randomly selected and placed in vials containing the test solutions. The commercial herbicide Logran (Novartis), containing 2-(2-chloroethoxy)-N-[[(4-methoxy-6-methyl-1,3,5-triazin-2-yl)amino]carbonyl] benzene-sulfonamide (Triasulfuron), was used as an internal positive reference.

Fractions were tested at concentrations of 1000, 600, 300, 100, 30, and 10 μM in a buffered nutritive aqueous solution (citric acid–sodium hydrogen phosphate buffer, pH 5.6; 2% sucrose). Stock solutions were prepared in DMSO and diluted with the buffer to achieve the desired concentrations, ensuring a final DMSO concentration of no more than 0.5% *v/v*. The control consisted of nutritive aqueous solution containing DMSO 0.5% *v/v*. Subsequent dilutions maintained the same buffer and DMSO concentrations.

Bioassays were conducted in 10 mL test tubes, with each tube containing five coleoptiles immersed in 2 mL of test solution. Three replicates were prepared for each test solution, and all experiments were performed in duplicate. The test tubes were placed in a roller tube apparatus and rotated at 6 rpm for 24 h at 22 °C in darkness. Results are displayed as percentage differences from the control in bar charts. A value of zero represents the control, while positive values indicate stimulation of the measured parameters, and negative values indicate inhibition [15].

### 3.3. Seed Germination Bioassay

The bioassays consisted of the germination of 25 seeds in the absence of light at 25 °C on 5 cm Petri plastic plates containing Whatman Nº 1 filter paper, and 5 mL of the test or control solution. The stock solutions were prepared using DMSO (0.1% *v/v*) and the test solutions were obtained from the dilution of that stock solution. The commercial herbicide Logran (Novartis), containing 2-(2-chloroethoxy)-*N*-[[(4-methoxy-6-methyl-1,3,5-triazin-2-yl)amino]carbonyl] benzenesulfonamide (Triasulfuron), was used as an internal reference.

Controls consisted of deionized water containing 0.1% (*v/v*) DMSO. Three repetitions of each treatment were carried out with concentrations of 100, 60, 30, 15, 5 m*M* and control. The species tested were as follows (incubation time between parentheses): *Allium cepa* L. (onion, monocotiledone, 5 days), *Lactuca sativa* L. (lettuce, dicotyledone, 5 days), and *Lepidum sativum* L. (garden cress, dicotyledone, 3 days). The statistical analysis of the data was carried out using Welch’s test. The significance levels were set at 0.01 and 0.05, meaning that differences were considered statistically significant if the probability of obtaining the observed results by chance was less than 1% (*p* < 0.01) or 5% (*p* < 0.05) [51].

## 4. Conclusions

In this study, five novel compounds were successfully synthesized from dehydrocostus lactone, a bioactive sesquiterpene lactone known for its diverse biological properties. A total of seven compounds were systematically evaluated for their coleoptile activity, assessing their potential effects on the elongation and growth of the coleoptile, an essential structure in seedling development. Additionally, five of these compounds were further tested for their herbicidal activity against three distinct plant species: *Allium cepa* (*onion*), *Lepidium sativum* (*garden cress*), and *Lactuca sativa* (*lettuce*). These plant species were selected due to their rapid germination and sensitivity to growth inhibitors, making them ideal models for herbicidal screening.

Among the lactone derivatives evaluated in this study, compound **4**, which was synthesized through the epoxidation of dehydrocostus lactone (DHC), emerged as the most potent inhibitor of plant growth. This compound demonstrated the highest inhibitory activity, significantly reducing the growth of *L. sativum*. Specifically, it exhibited an 80% inhibition of stalk length and an even greater 88% inhibition of radicle length of garden cress, indicating a strong growth-suppressing effect. In contrast, the majority of the other tested compounds stimulated the growth of *L. sativum*, further emphasizing the unique and powerful inhibitory nature of compound **4**. These findings suggest that this compound holds promising potential as a natural herbicidal agent, capable of effectively limiting plant development.

A possible explanation for the enhanced biological activity of compound **4** lies in the presence of two epoxide functional groups within its molecular structure. Epoxides are well known for their high electrophilicity, making them highly reactive toward nucleophilic attack by biomolecules, including plant enzymes. This reactivity allows epoxides to interact with key enzymatic pathways involved in plant growth and development, potentially leading to enzyme inhibition or disruption of metabolic processes essential for cell division and elongation. Consequently, the presence of two epoxide groups in compound **4** may significantly enhance its herbicidal potency, as it provides multiple reactive sites that increase its likelihood of interfering with plant biochemical mechanisms. This structural feature likely accounts for the superior inhibitory effects observed in the growth of *L. sativum* when compared to other tested compounds.

Future studies should focus on evaluating the stability of the synthesized compounds in environmental conditions, particularly in soil and water, to assess their biodegradability. Understanding the degradation pathways and persistence of these compounds is essential for determining their environmental impact and ensuring their suitability for agricultural applications. Additionally, bioassays should be conducted to assess the effects of the synthesized compounds on beneficial organisms, such as natural enemies of pests and pollinators, including ladybirds and bees. Evaluating their impact on these non-target species is crucial for determining the ecological safety of the compounds.

## Figures and Tables

**Table 1:** Nuclear magnetic resonance data of DHC.

*δ*_H_	Hydrogen	COSY	*δ*_C_	Carbon
0.82–0.72	8′	8 × 8′, 9 × 8′,7 × 8′	30.7	8
1.60–1.46	2,8,9′	9′ × 14, 7 × 8, 8′ × 8, 9′ × 9, 1 × 2	30.4	2
1.98	9	8′, 9′, 7, 14	32.8	3
2.11	7	6 × 7,7 × 8, 7 × 8′, 7 × 13, 7 × 13′	36.4	9
2.41–2.20	1,3,3′,5 (m)	1 × 2, 1 × 2′, 3 × 2, 3 × 2′, 1 × 14	44.7	7
3.48	6 (t, *J* = 8 Hz)	6 × 7, 5 × 6	47.5	5
4.60	14,14′ (m)	1 × 14, 9 × 14, 9′ × 14	52.1	1
4.87	13′ (d, *J* = 4 Hz)	13′ × 7, 13 × 13′	84.4	6
5.07	15′ (m)	15′ × 5, 15′ × 3, 15′ × 3′	109.7	15
5.46	15 (m)	15 × 5, 15 × 3, 15 × 3′	112.0	14
6.11	13 (d, *J* = 4 Hz)	13 × 7, 13 × 13′	118.8	13
			140.7	4
			149.6	10
			151.4	11
			169.2	C=O

## Data Availability

Data is contained within the article and Supplementary Materials.

## Supplementary Materials

The following supporting information can be downloaded at: https://www.mdpi.com/article/10.3390/plants14071111/s1, Figures S1–S40; Tables S1–S11.
