# Supplementary material for: Exploring Sesquiterpene Lactones from Saussurea lappa: Isolation, Structural Modifications, and Herbicide Bioassay Evaluation
Source: Plants (Basel). 2025 Apr 2;14(7):1111. doi: 10.3390/plants14071111 (PMC11991101; doi:10.3390/plants14071111)
Supplement: Supplementary file 1 [file plants-14-01111-s001.zip › plants-3538131-supplementary.pdf]

Isolation and Structural Modification of Sesquiterpene Lactones from *Saussurea lappa*: Evaluation through Herbicide Bioassays

Elson S. de Alvarenga[1], Francisco A. Macías[2], Stephani S. Ferreira[1], Juan C. G. Galindo[2], José M. G. Molinillo[2]

[1]Department. of Chemistry, Universidade Federal de Viçosa, Viçosa, MG, 36570-900, Brazil,

[2]Dept. of Organic Chemistry, Faculty of Sciences, University of Cádis; c/ República Saharui s/n, 11510-Puerto Real, Cádiz, Spain

* Corresponding author: elson@ufv.br

Supplementary Information

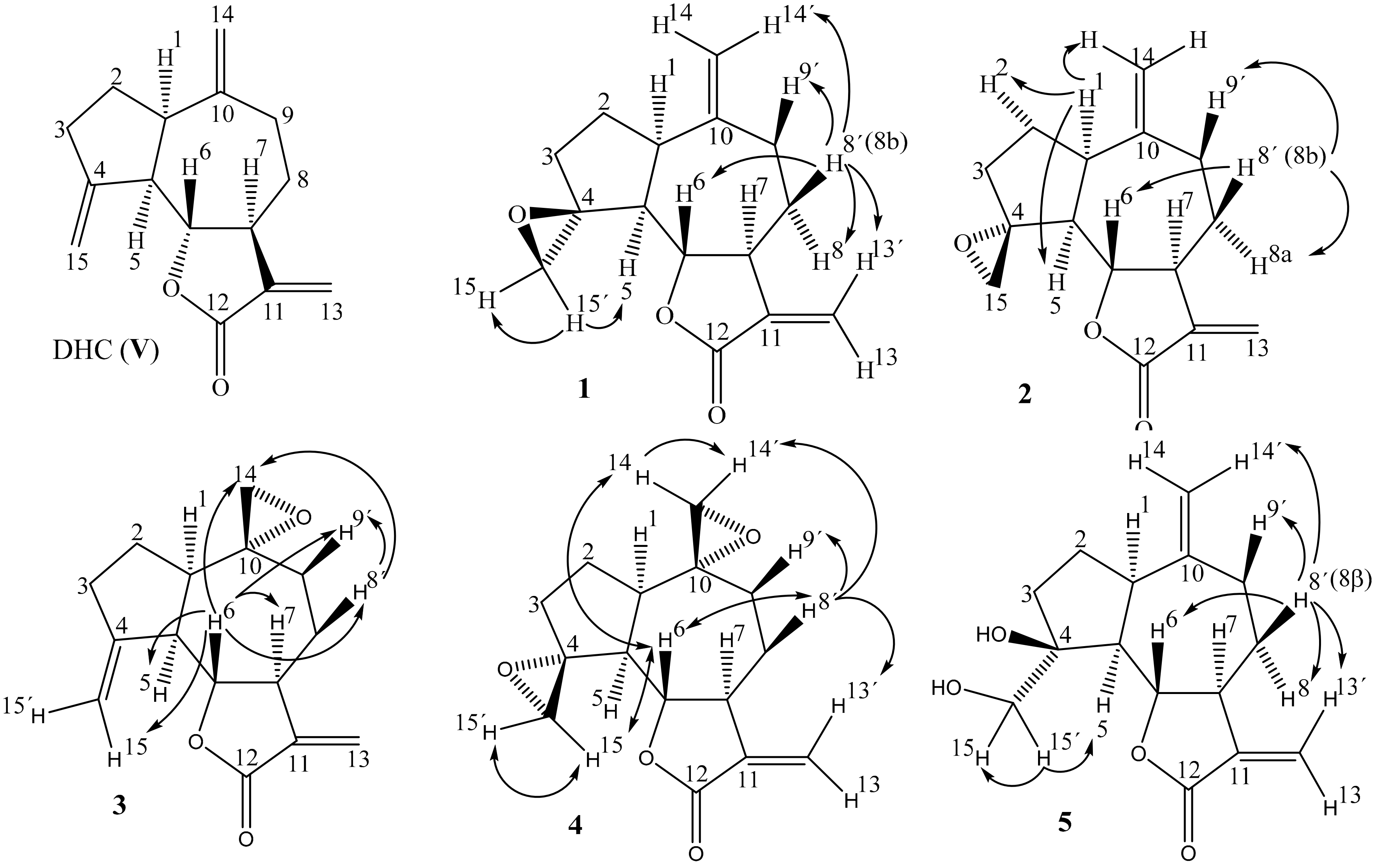

Figure S1: NOE effects observed for the epoxy derivatives (**1-5**).

Table S1: ^{13}C NMR chemical shifts of compounds (**1-5**).

CARBON	Carbon Shift	1	2	3	4	5
**1**	52.1	47.02	45.4	45.3	47.4	46.3
**2**	30.4	28.7	26.7	24.5	22.4	29.4
**3**	32.8	31.7	32.4	32.3	32.7	33.4
**4**	140.7	65.8	57.1	151.4	64.6	64.3
**5**	47.5	53.4	50.3	50.5	43.6	49.0
**6**	84.4	81.0	83.4	83.4	80.4	81.0
**7**	44.7	45.5	44.3	44.2	44.1	44.3
**8**	30.7	29.6	24.4	26.6	27.1	31.0
**9**	36.4	33.1	35.5	35.4	38.6	38.5
**10**	149.6	140.5	140.4	57.1	56.2	149.3
**11**	151.4	148.6	151.4	140.3	139.5	139.9
**13**	118.8	118.6	118.9	119.1	119.3	112.5
**14**	112.0	113.5	108.9	49.3	47.3	118.9
**15**	109.7	49.6	49.4	108.8	48.4	47.6
**C=O**	169.2	168.7	168.9	169.1	168.9	169.1

Table S2: Experimental parameters used in the NMR experiments.

Parameter	^{1}H NMR	^{13}C NMR	DEPT 135	HSQC	HMBC	COSY	NOESY
Manufacturer	Varian	Varian	Varian	Varian	Varian	Varian	Varian
Pulse Sequence	s2pul	s2pul	dept	HSQC	HMBC	COSY	NOESY
Probe	ASW-PFG	ASW-PFG	ASW-PFG	ASW-PFG	ASW-PFG	ASW-PFG	ASW-PFG
Number of Scans	128	256	128	4	40	4	64
Receiver Gain	60	60	54	60	60	60	60
Relaxation Delay	0.0000	10.000	10.000	1.0000	1.0000	1.0000	1.0000
Pulse Width	0.0000	0.0000	0.0000	0.0000	0.0000	0.0000	0.0000
Nucleus	^{1}H	^{13}C	^{13}C	^{1}H,^{13}C	^{1}H,^{13}C	^{1}H,^{1}H	^{1}H,^{1}H
Acquisition Time	29.999	11.994	11.994	0.2438	0.2438	0.2438	0.2438
Spectrometer Frequency	399.94	100.58	100.58	399.94,100.57	399.94, 100.58	399.94,399.94	399.94, 399.94
Spectral Width	4199.5	25141.4	26648.9	4199.5,7097.7	4199.5, 24132.7	4199.5,4199.5	4199.5,4199.5
Lowest Frequency	-370.8	-1493.7	-1507.9	-370.8, -980.9	-404.1, -1481.1	-370.8,-370.8	-371.5, -371.5
Acquired Size	12598	30154	31962	1024, 128	1024, 400	1024,128	1024, 128
Spectral Size	32768	65536	65536	1024/1024	1024/1024	1024,1024	1024, 1024

Table S3: Nuclear magnetic resonance data of DHC.

δ_H	Hydrogen	COSY	δ_C	Carbon
0.82-0.72	8´	8x8´, 9x8´,7x8´	30.7	8
1.60-1.46	2,8,9´	9´x14, 7x8, 8´x8, 9´x9, 1x2	30.4	2
1.98	9	8´, 9´, 7, 14	32.8	3
2.11	7	6x7,7x8, 7x8´, 7x13, 7x13´	36.4	9
2.41 - 2.20	1,3,3´,5 (m)	1x2, 1x2´, 3x2, 3x2´, 1x14	44.7	7
3.48	6 (t, *J*=8Hz)	6x7, 5x6	47.5	5
4.60	14,14´ (m)	1x14, 9x14, 9´x14	52.1	1
4.87	13´ (d, *J*=4Hz)	13´x7, 13x13´	84.4	6
5.07	15´ (m)	15´x5, 15´x3, 15´x3´	109.7	15
5.46	15 (m)	15x5, 15x3, 15x3´	112.0	14
6.11	13 (d, *J*=4Hz)	13x7, 13x13´	118.8	13
			140.7	4
			149.6	10
			151.4	11
			169.2	C=O

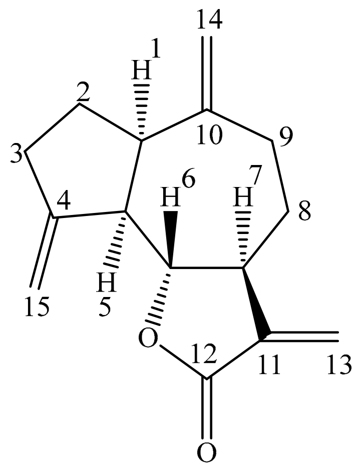

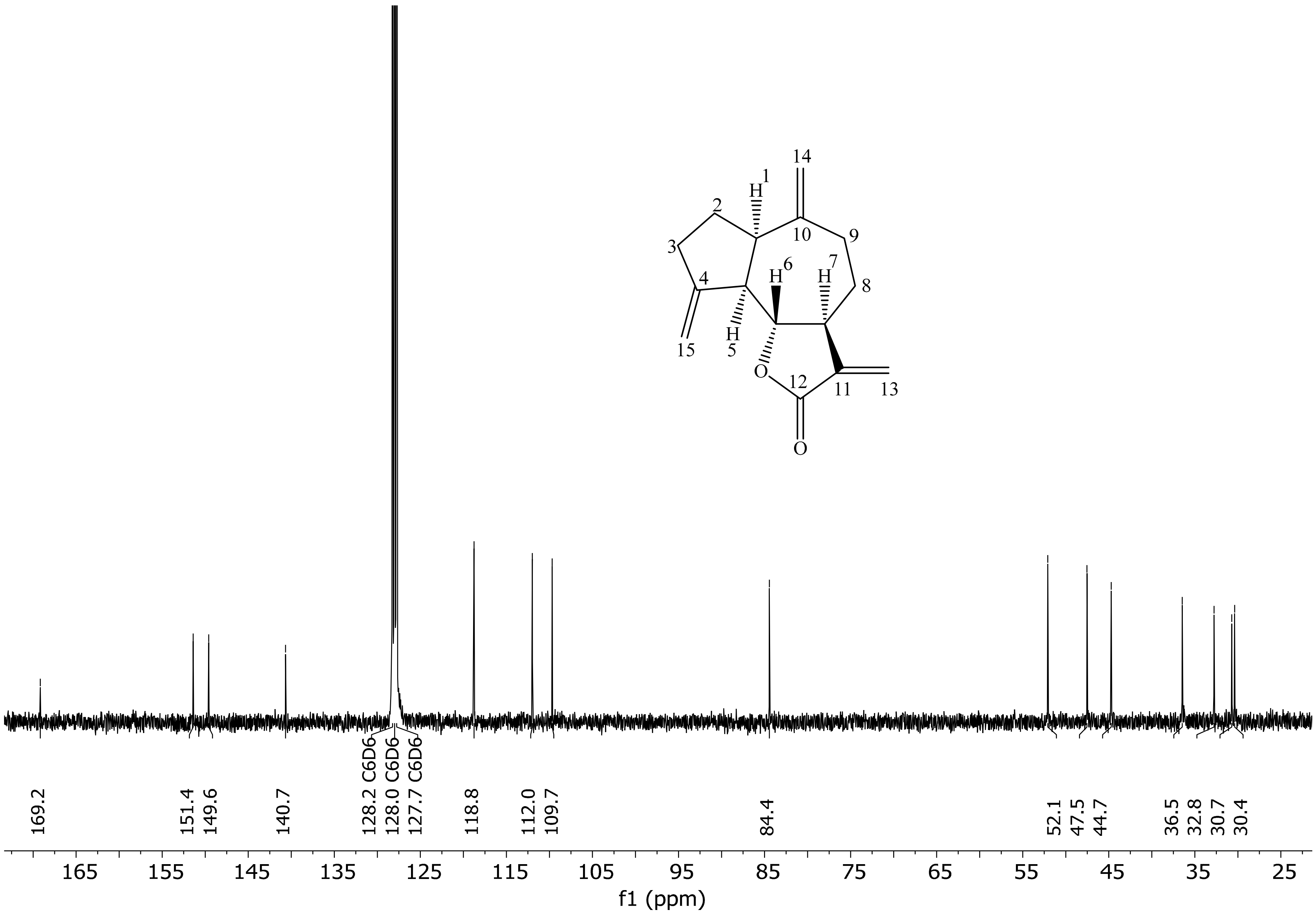

Figure S2: ^{13}C NMR spectrum (C_6D_6) of DHC (100 MHz)

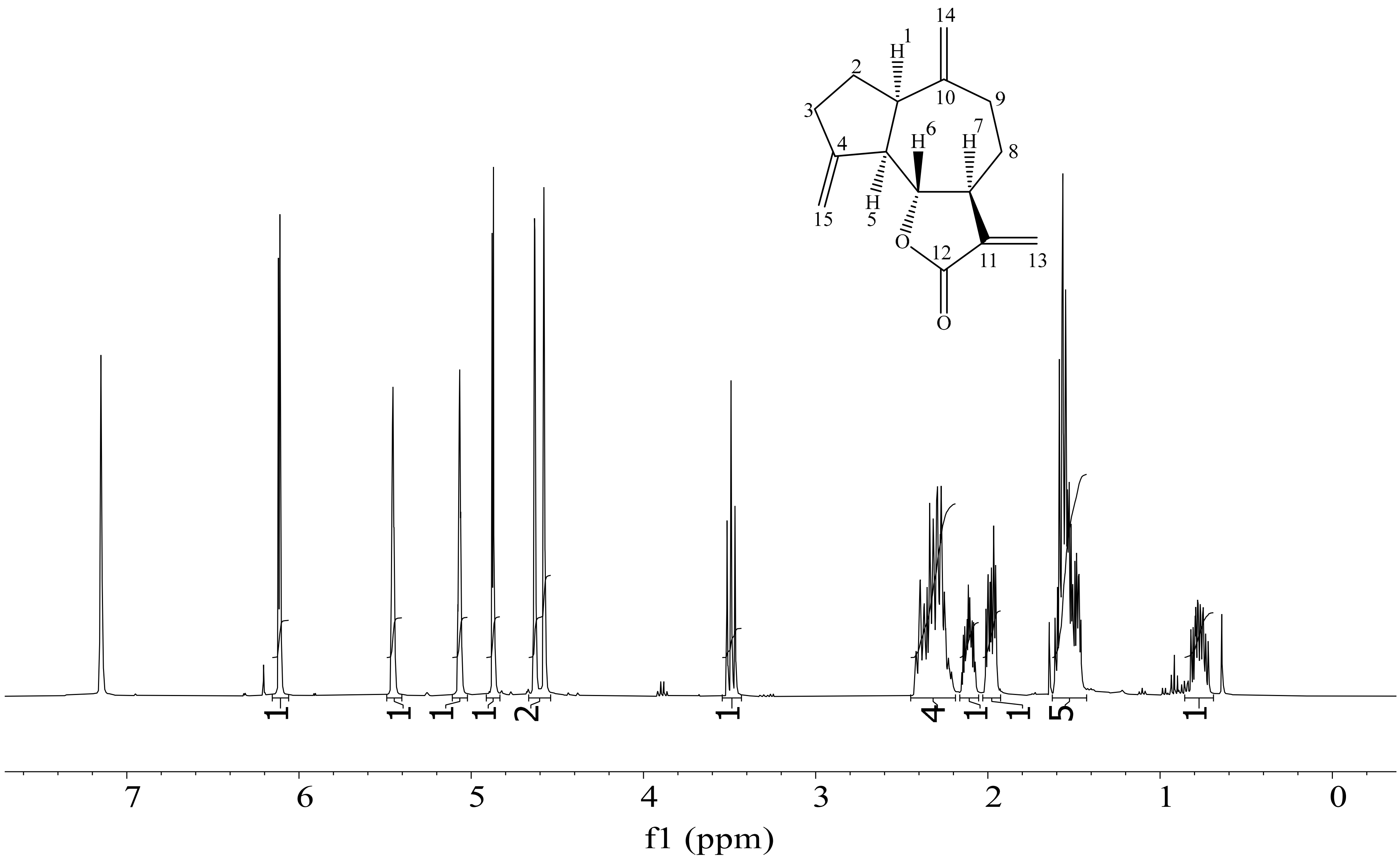

Figure S3: ^{1}H NMR spectrum (C_6D_6) of DHC (400 MHz).

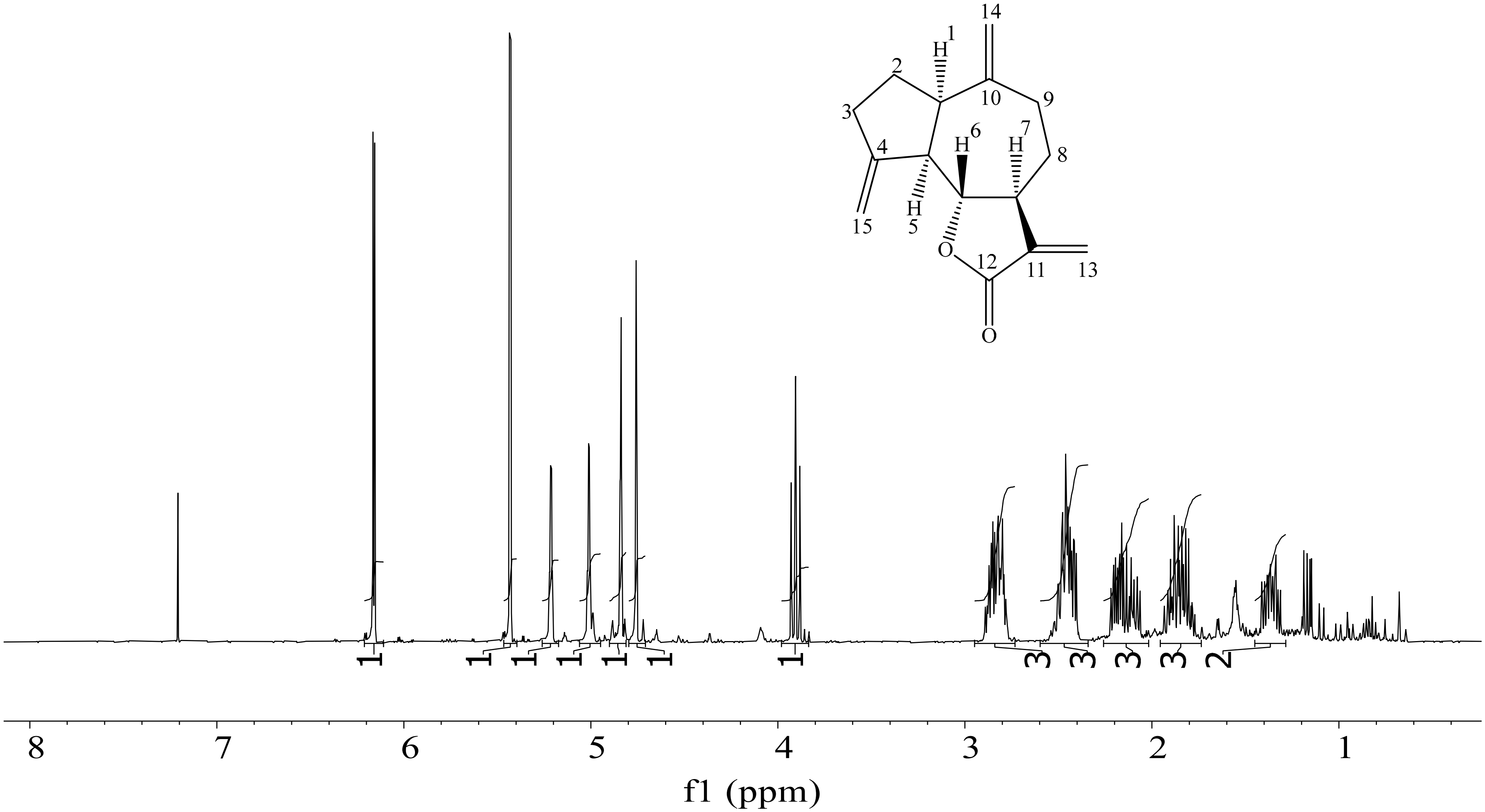

Figure S4: ^{1}H NMR spectrum ($CDCl_3$) of DHC (400 MHz).

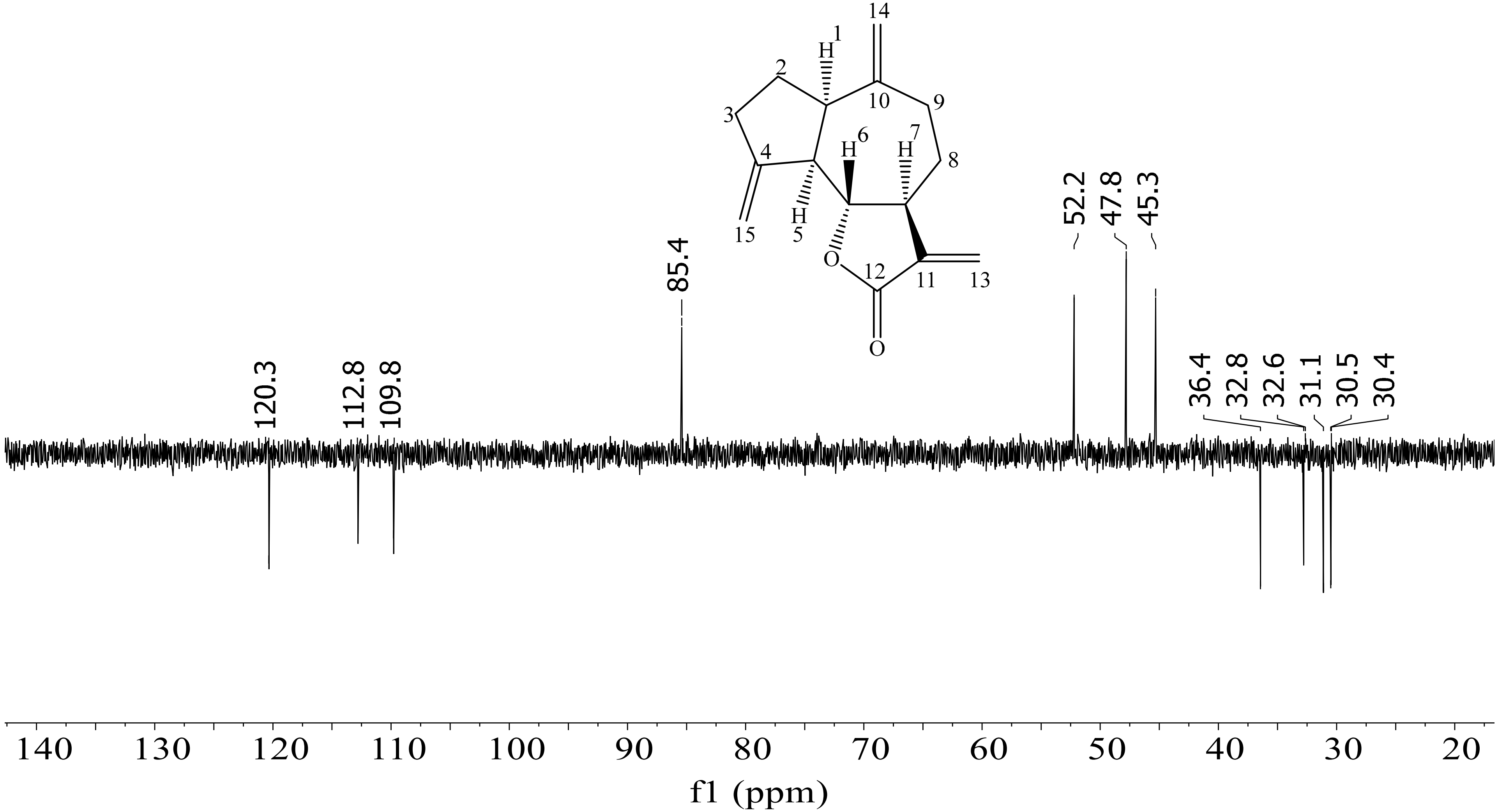

Figure S5: DEPT spectrum ($CDCl_3$) of DHC (100 MHz).

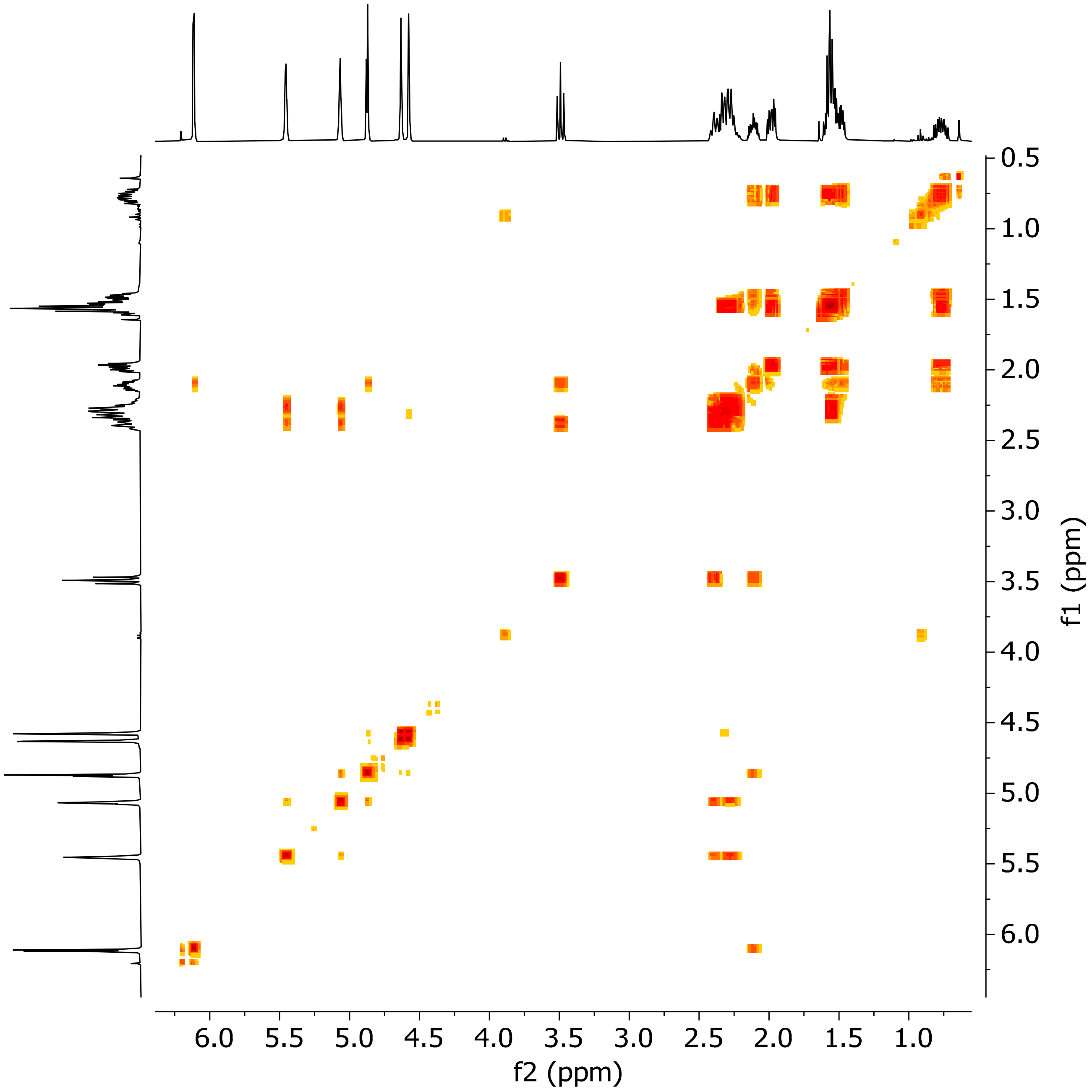

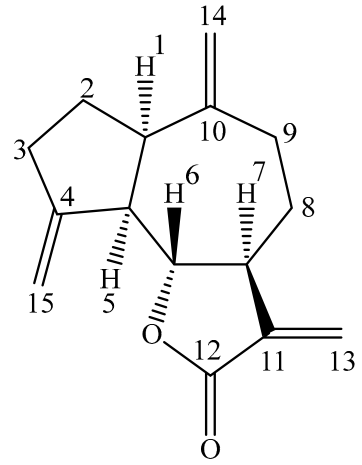

Figure S6: COSY spectrum (C_6D_6) of DHC (400 MHz).

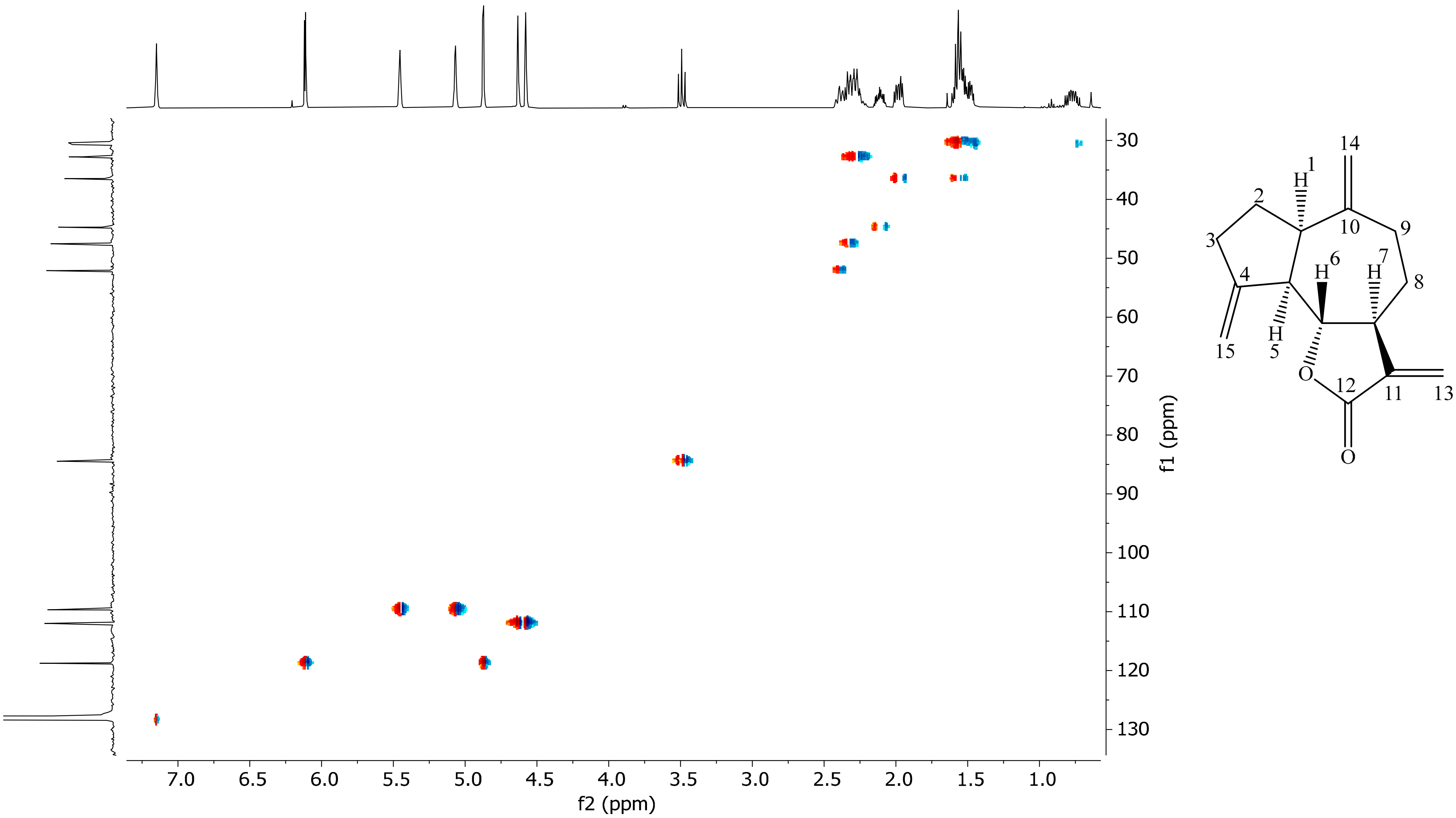

Figure S7: HSQC spectrum (C_6D_6) of DHC (400 MHz).

Table S4: (3a*S*,9*S*,9b*S*)-3,6-dimethylenedecahydro-2*H*-spiro[azulene[4,5-b] furan-9,2'-oxiran]-2-one **(1)** nuclear magnetic resonance data

δ_H	Hydrogen	COSY	δ_C	Carbon
0.67-0.77	8´ (m)	8x8´, 9x8´,7x8´, 9´x8	28.7	2
1.38-1.56	2', 3´, 8, 9´(m)	2x2´, 9´x14, 7x8, 8´x8, 9´x9, 1x2´, 3´x3, 2'x3´	29.6	8
1.71-1.94	2, 3, 5, 7, 9 (m)	8x9, 9x9´, 6x7, 7x13, 9x14, 5x6, 1x2, 2x3´	31.7	3
2.56	15´ (4.8Hz)	15x15´	33.1	9
2.76	1 (q, 8Hz)	1x2, 1x2´, 1x14´, 1x14	45.5	7
3.23	6 (dd, *J*=8.8, 11.0Hz)	6x5, 6x7	47.02	1
3.31	15 (4.8Hz)	15x15´	49.6	15
4.61	14´ (d, *J*=4.8Hz)	14´x14, 14´x1, 14´x9, 14´x9´	53.4	5
4.64	14 (d, *J*=4.8Hz)	14´x14, 14´x1, 14´x9, 14´x9´	65.8	4
4.81	13´ (dd,*J*=0.4, 2.8Hz)	13´x13, 13´x7	81.0	6
6.06	13 (dd, *J*=0.4, 2.8Hz)	13x7, 13x13´	113.5	14
			118.6	13
			140.5	10
			148.6	11
			168.7	C=O

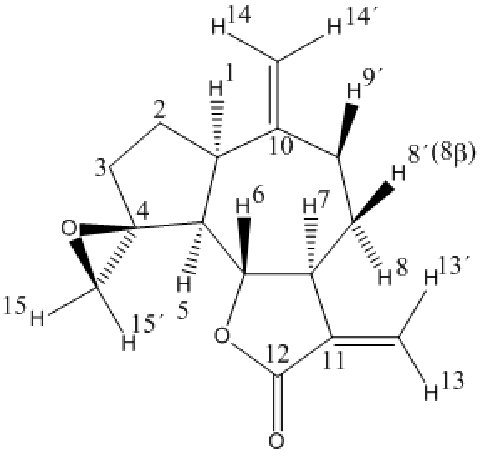

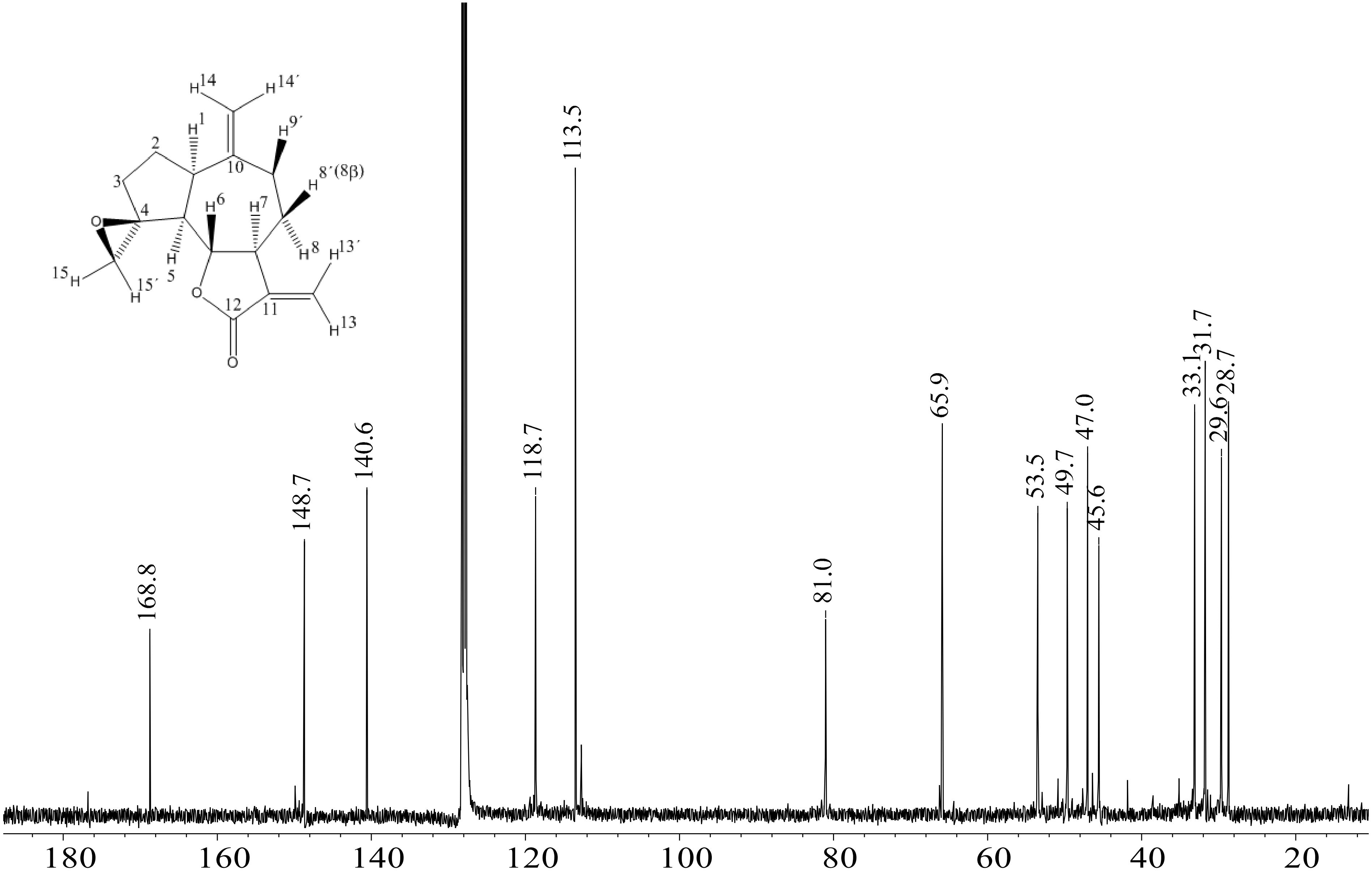

Figure S8: ^{13}C NMR spectrum (C_6D_6) of (3aS,6aR,9S,9aS,9bS)-3,6-dimethylenedecahydro-2H-spiro[azuleno[4,5-b]furan-9,2'-oxiran]-2-one **(1)** (100 MHz).

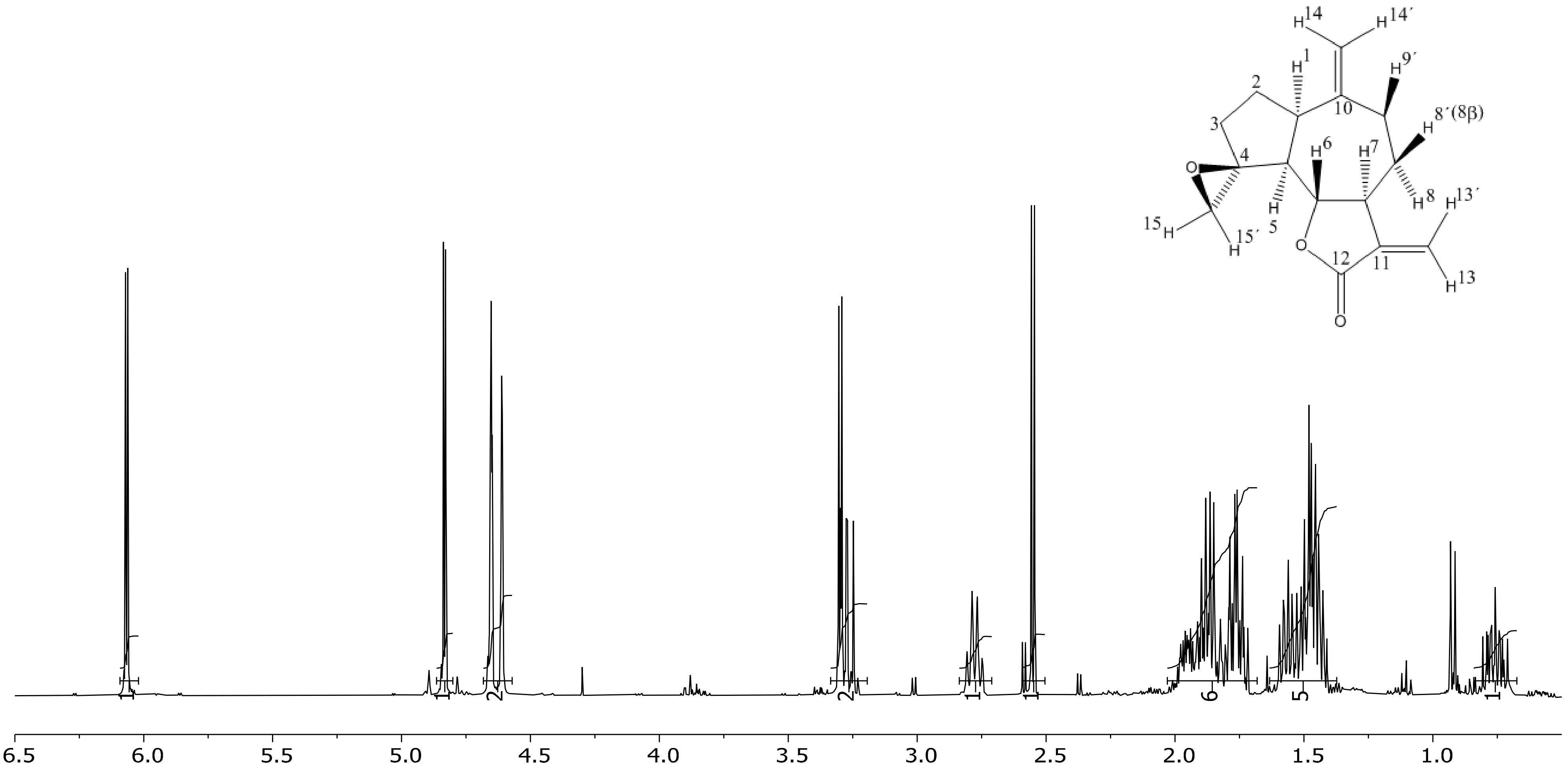

Figure S9: ^{1}H NMR spectrum (C_6D_6) of (3aS,6aR,9S,9aS,9bS)-3,6-dimethylenedecahydro-2H-spiro[azuleno[4,5-b]furan-9,2'-oxiran]-2-one **(1)** (400 MHz).

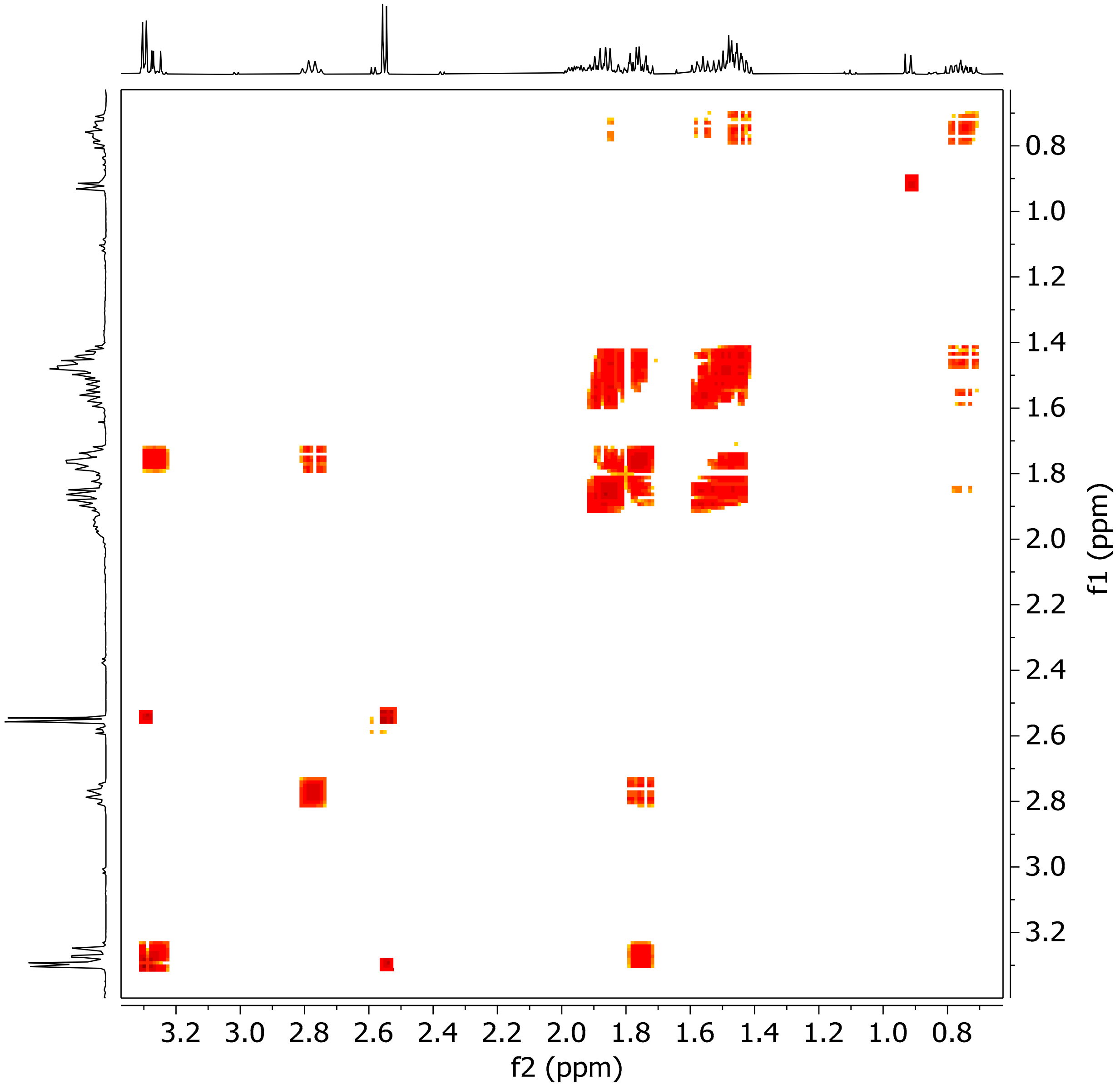

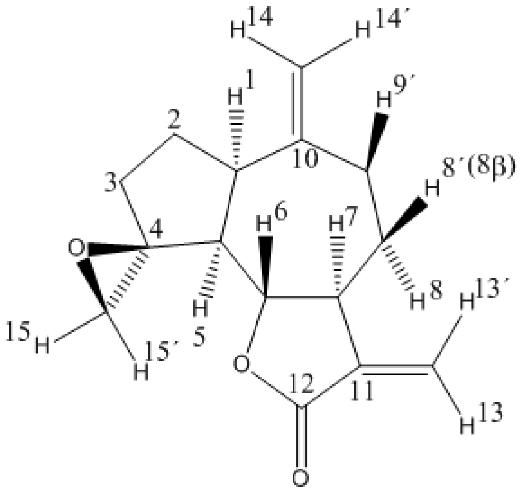

Figure S10: COSY spectrum (C_6D_6) of (3aS,6aR,9S,9aS,9bS)-3,6-dimethylenedecahydro-2H-spiro[azuleno[4,5-b]furan-9,2'-oxiran]-2-one **(1)** (400 MHz).

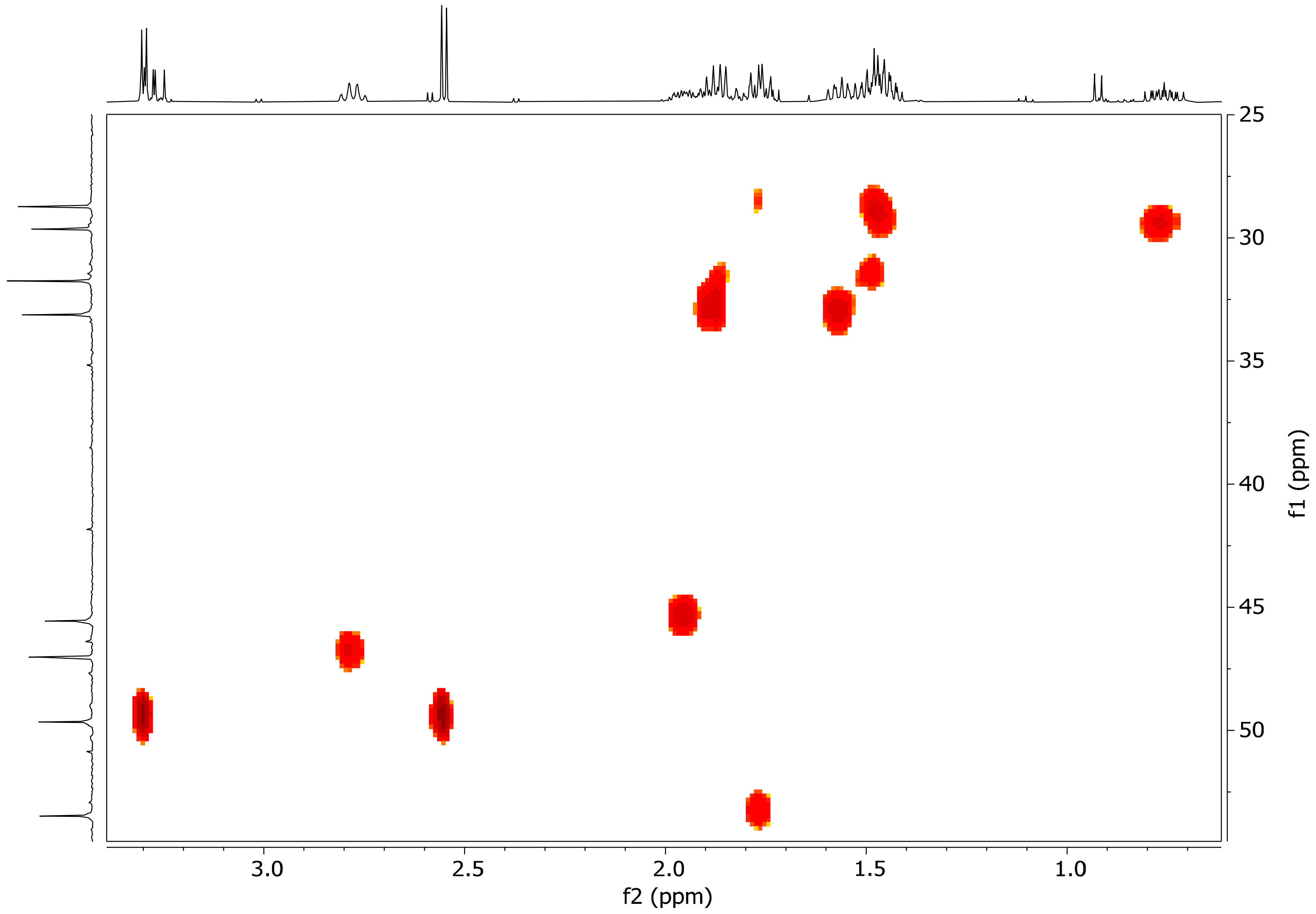

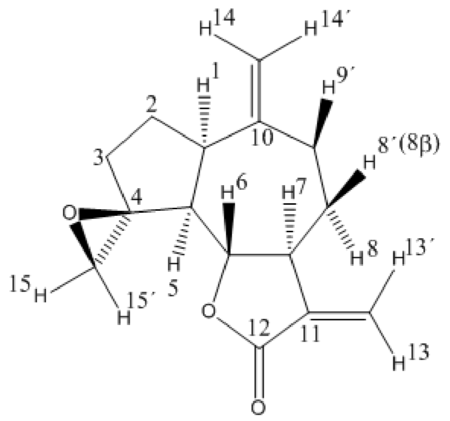

Figure S11: HSQC spectrum (C_6D_6) of (3aS,6aR,9S,9aS,9bS)-3,6-dimethylenedecahydro-2H-spiro[azuleno[4,5-b]furan-9,2'-oxiran]-2-one **(1)** (400 MHz).

Table S5: Nuclear magnetic resonance data of (3a*S*,6a*R*,9*R*,9a*S*,9b*S*)-3,6-dimethylenedecahydro-2*H*-spiro[azuleno[4,5-b]furan-9,2'-oxiran]-2-one **(2)**

^{1}H shift	Hydrogen	^{13}C shift	Carbon
0.60-0.77	8´ (m)	24.4	8
1.29-1.36	8 (m)	26.7	2
1.40-1.54	2, 9, 9´ (m)	32.4	3
1.63-1.77	2´,3 (m)	35.5	9
1.87-1.96	5, 3´ (m)	44.3	7
2.10-2.15	7 (dt, *J*=4, 12Hz)	45.4	1
2.22-2.29	1 (m)	49.4	15
3.32	15´ (d, *J*=12Hz)	50.3	5
3.80-3.83	6 (m), 15 (d, *J*=12Hz)	57.1	4
4.74	14´ (m)	83.4	6
4.82	13´ (d, *J*=3.2Hz)	108.9	14
4.88	14 (m)	118.9	13
6.03	13 (d, *J*=3.2 Hz)	140.4	10
		151.4	11
		168.9	C=O

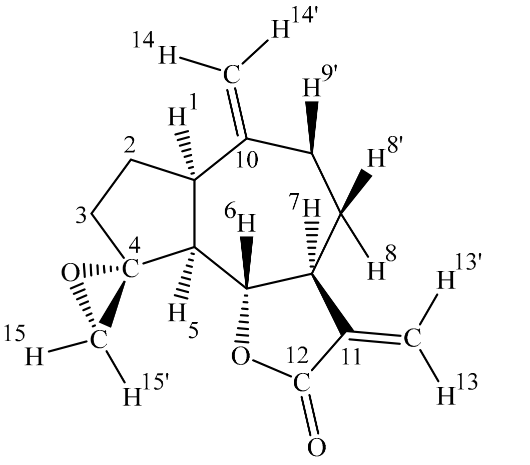

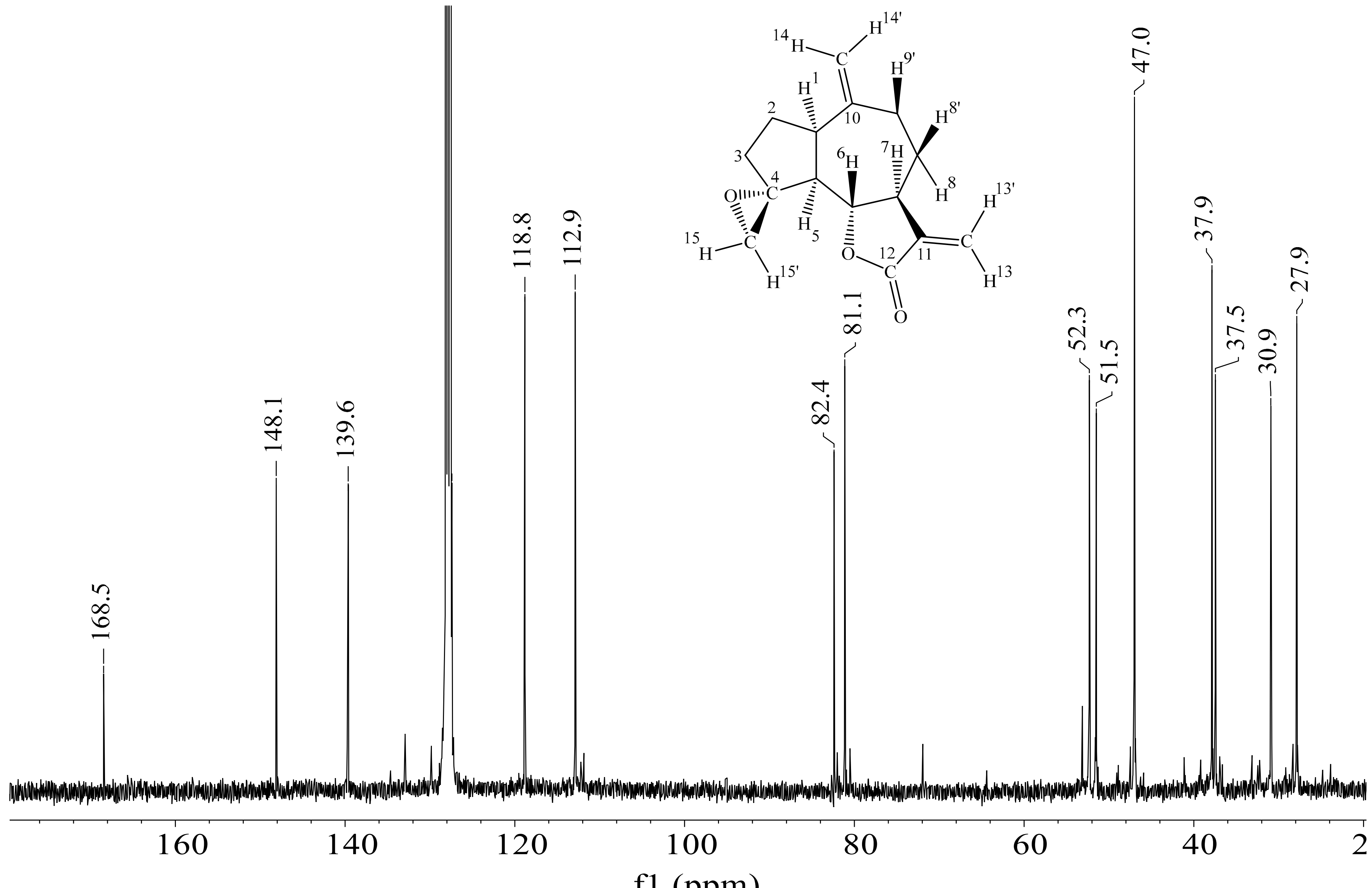

Figure S12: ^{13}C NMR spectrum (C_6D_6) of (3a*S*,6a*R*,9*R*,9a*S*,9b*S*)-3,6-dimethylenedecahydro-2*H*-spiro[azuleno[4,5-b]furan-9,2'-oxiran]-2-one **(2)** (100 MHz).

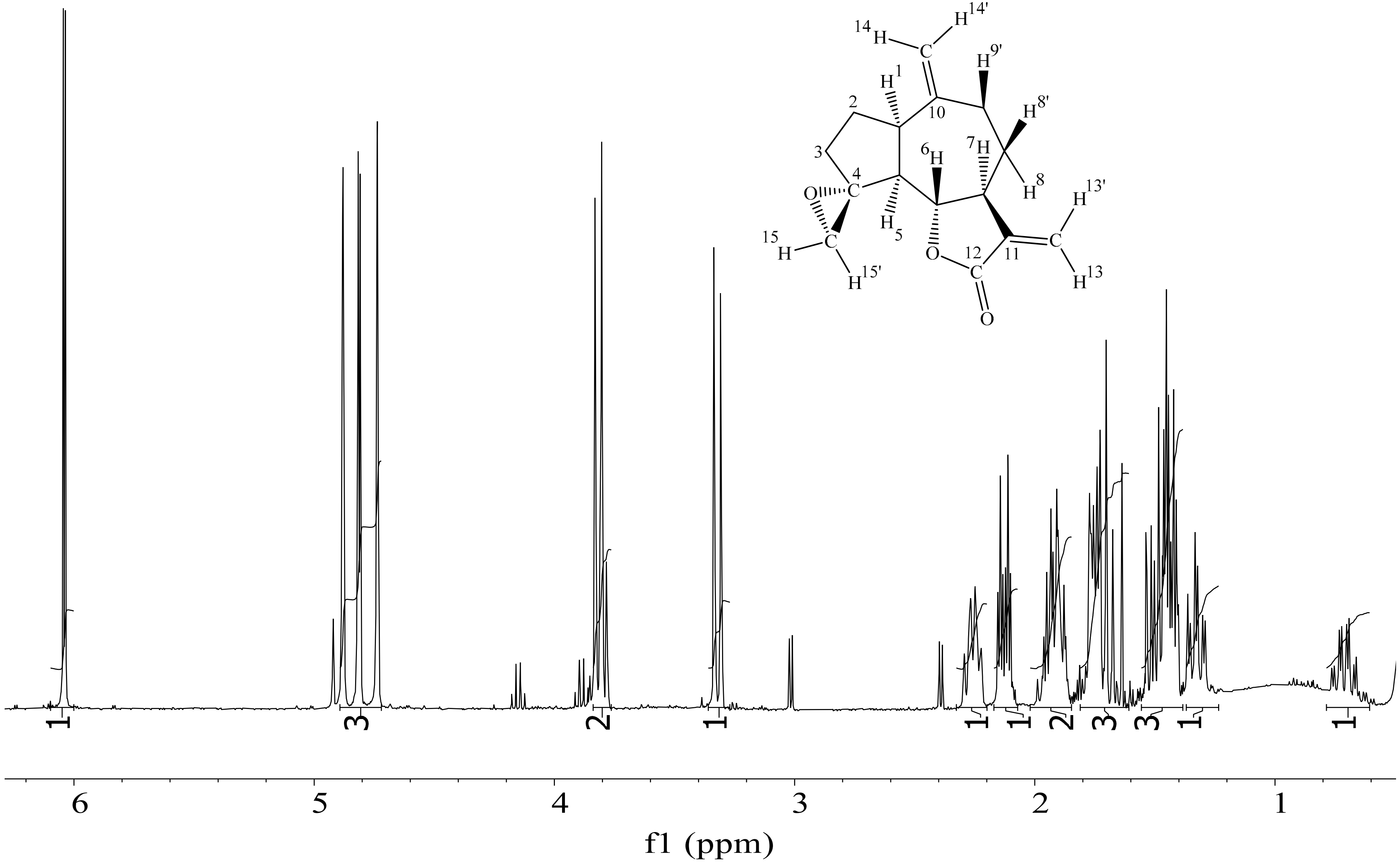

Figure S13: ^{1}H NMR spectrum (C_6D_6) of (3a*S*,6a*R*,9*R*,9a*S*,9b*S*)-3,6-dimethylenedecahydro-2*H*-spiro[azuleno[4,5-b]furan-9,2'-oxiran]-2-one **(2)** (400 MHz).

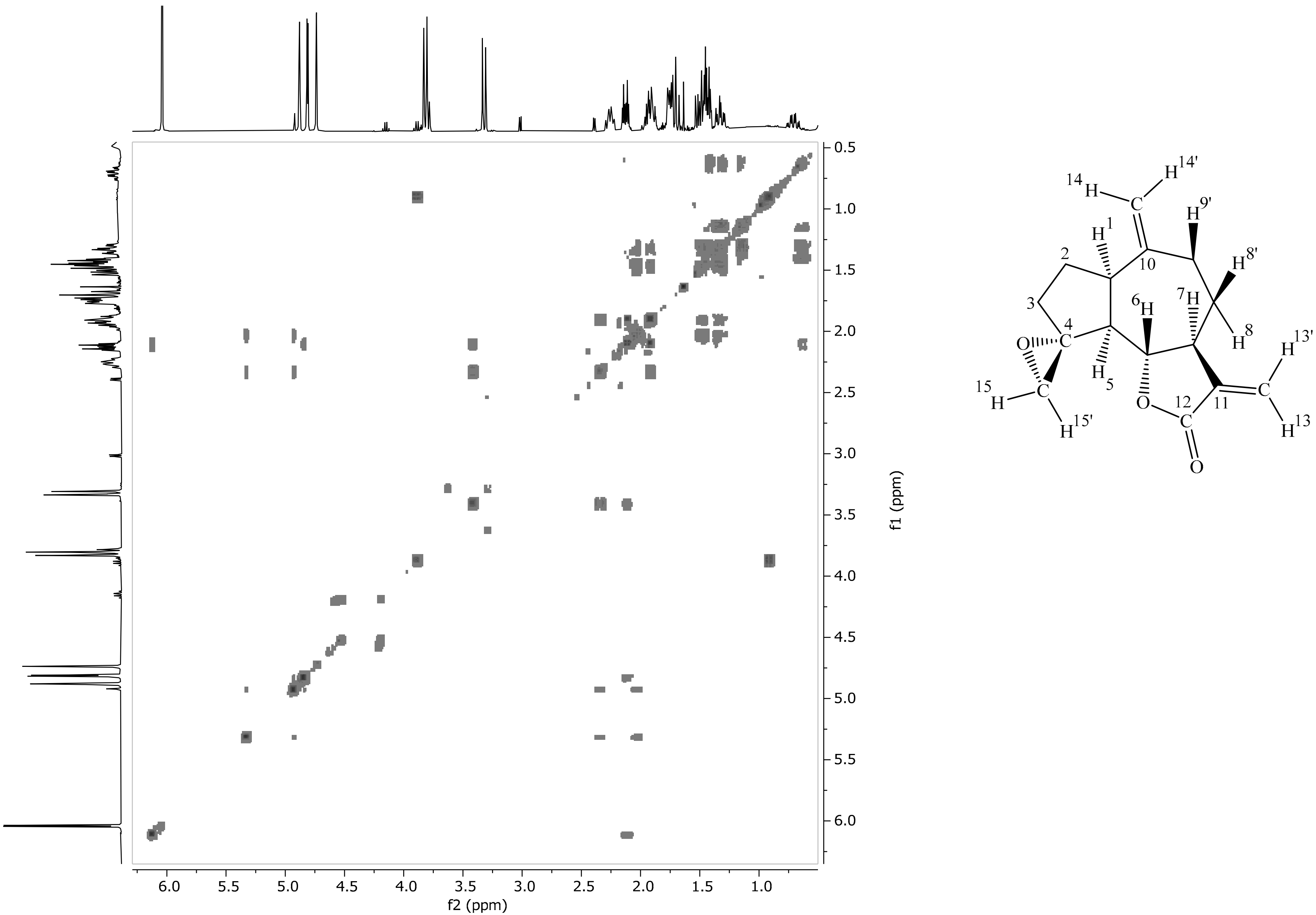

Figure S14: COSY spectrum (C_6D_6) of (3a*S*,6a*R*,9*R*,9a*S*,9b*S*)-3,6-dimethylenedecahydro-2*H*-spiro[azuleno[4,5-b]furan-9,2'-oxiran]-2-one **(2)** (400 MHz).

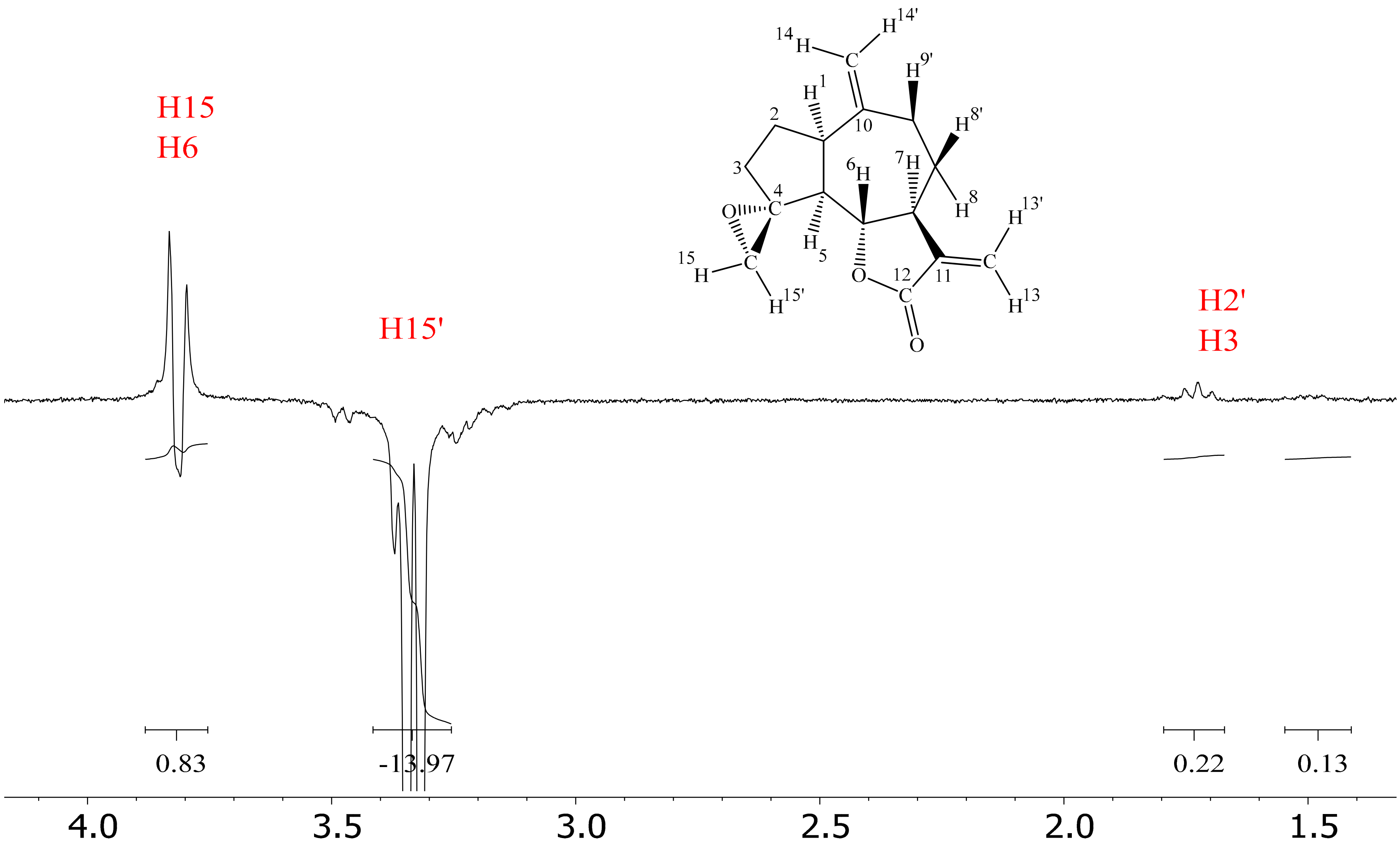

Figure S15: NOE difference NMR spectrum (C_6D_6) of (3a*S*,6a*R*,9*R*,9a*S*,9b*S*)-3,6-dimethylenedecahydro-2*H*-spiro[azuleno[4,5-b]furan-9,2'-oxiran]-2-one **(2)** (400 MHz).

Table S6: Nuclear magnetic resonance data of (3a*S*,6*R*,6a*R*,9a*R*,9b*S*)-3,9-dimethylenedecahydro-2*H*-spiro[azuleno[4,5-b]furan-6,2'-oxiran]-2-one **(3)**

δ_H	Hydrogen	HMBC	δ_C	Carbon
0.59-0.69	8′ (m)	C2-H1	24.5	2
1.12-1.18	9′(m)	C8- H6, C8-H9′	26.6	8
1.27-1.53	2, 2′, 8, 9 (m)	C3-H1	32.3	3
1.88-1.96	1, 14′ (m)	C9-H1, C9-H14′	35.4	9
2.76	7, 3, 3′, 7, 14 (m)	C7-H13, C7-H13′, C7-H9, C7-H9′, C7-H8′	44.2	7
3.23	5	C1-H9, C1-H9′, C1-H2	45.3	1
2.35	5 (m)	C14-H1, C14-H9	49.3	14
3.42	6 (dd, *J*=8, 10Hz)		50.5	5
4.84	13′ (d, *J*=2.8Hz)	C7-H13′, C12-H13′		
		C10-H14′, C10-H14, C10-H2, C10-H8, C10-H9′	57.1	10
4.93	15′(m)	C6 - H8	83.4	6
5.32	15 (m)		108.8	15
			119.1	13
		C11 - H13	140.3	11
			151.4	10
		C12-H13, C12-H13′	169.1	C=O

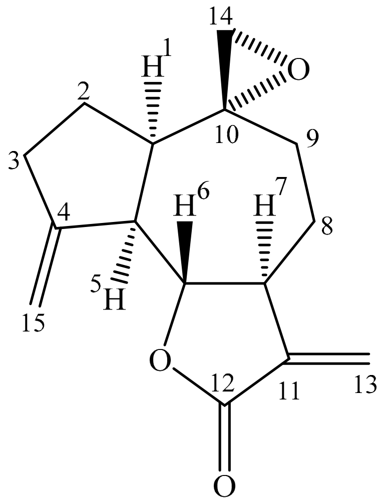

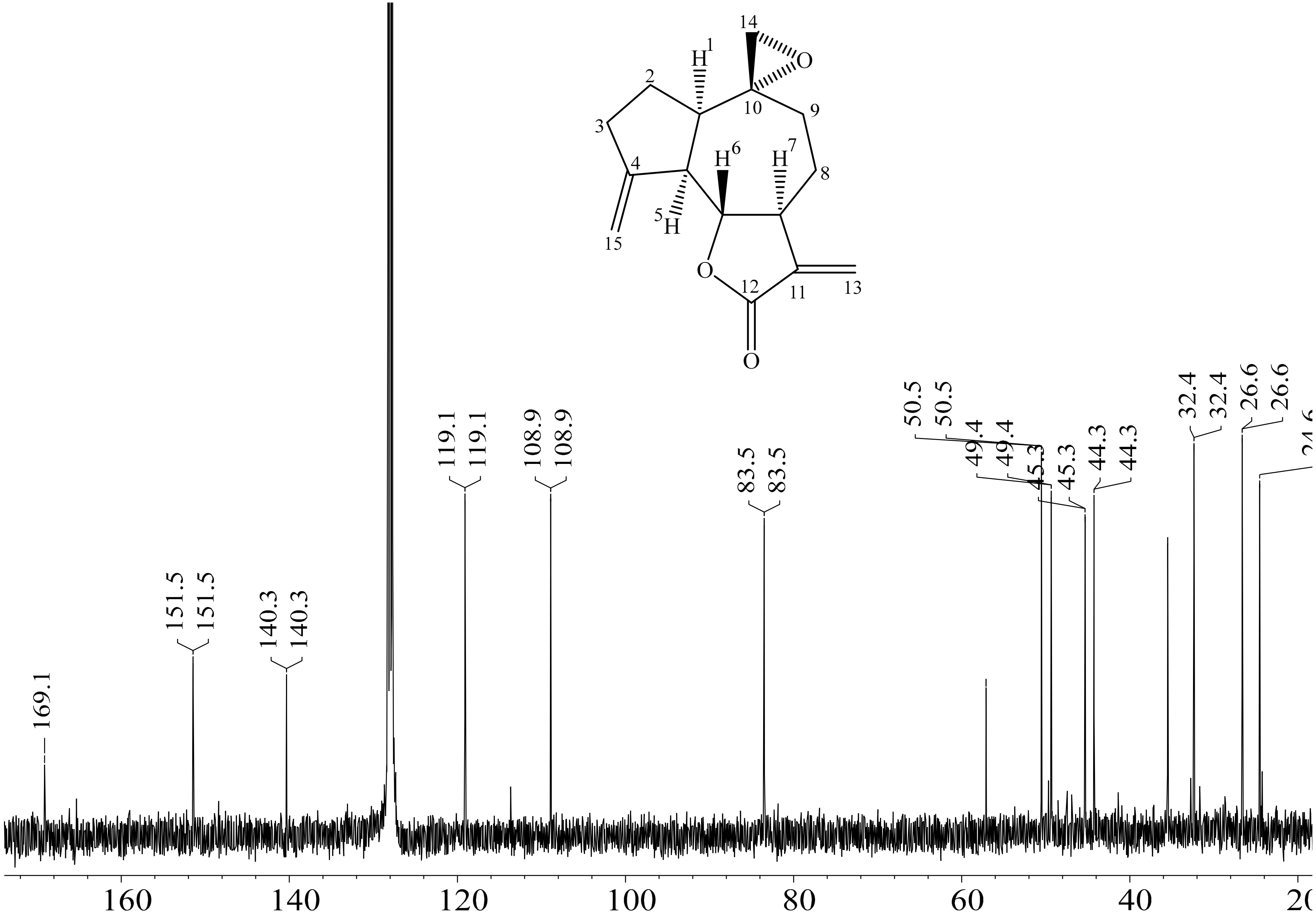

Figure S16: ^{13}C NMR spectrum (C_6D_6) of (3a*S*,6*R*,6a*R*,9a*R*,9b*S*)-3,9-dimethylenedecahydro-2*H*-spiro[azuleno[4,5-b]furan-6,2'-oxiran]-2-one **(3)** (100 MHz).

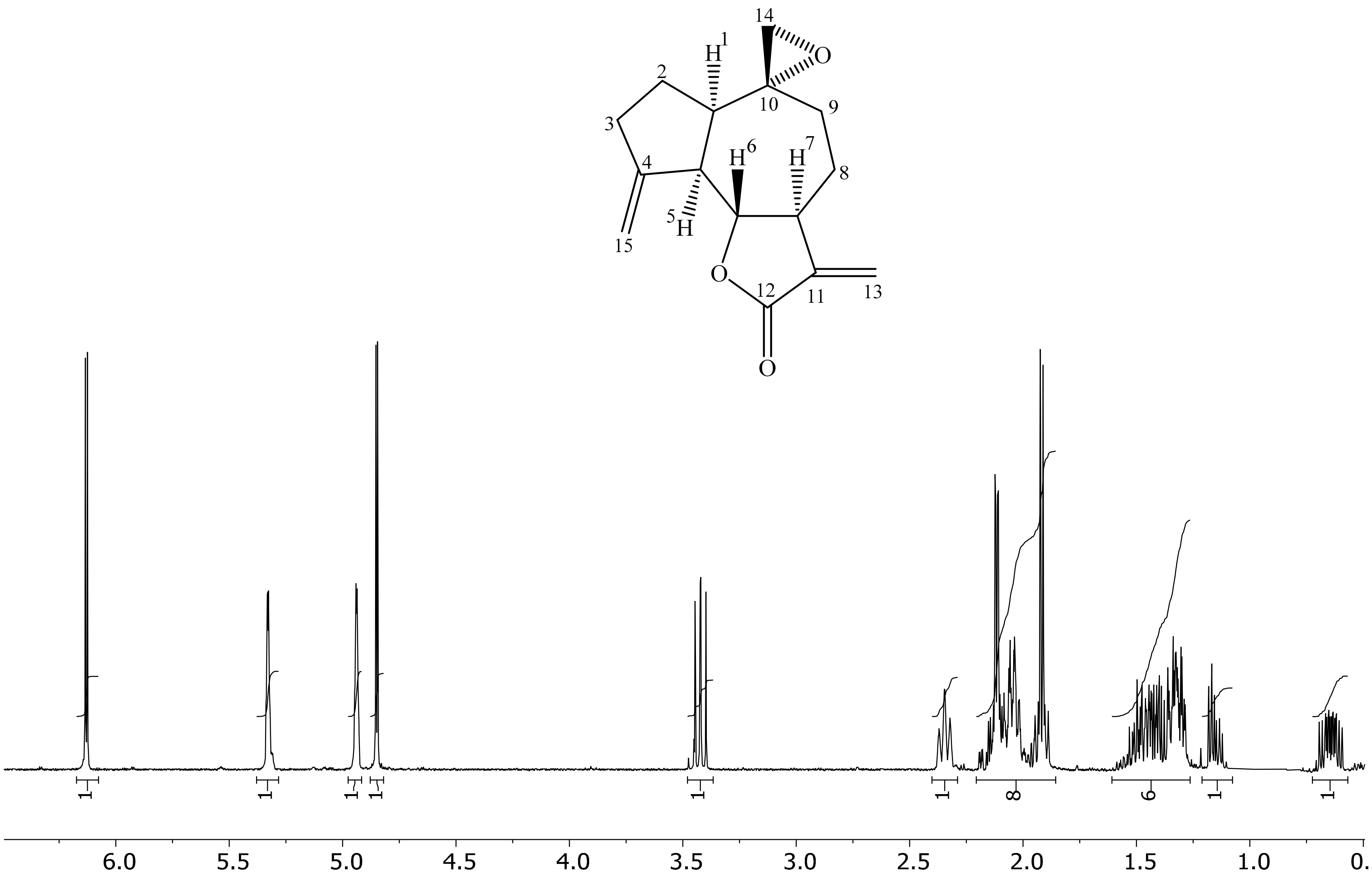

Figure S17: ^{1}H NMR spectrum (C_6D_6) of (3a*S*,6*R*,6a*R*,9a*R*,9b*S*)-3,9-dimethylenedecahydro-2*H*-spiro[azuleno[4,5-b]furan-6,2'-oxiran]-2-one **(3)** (400 MHz).

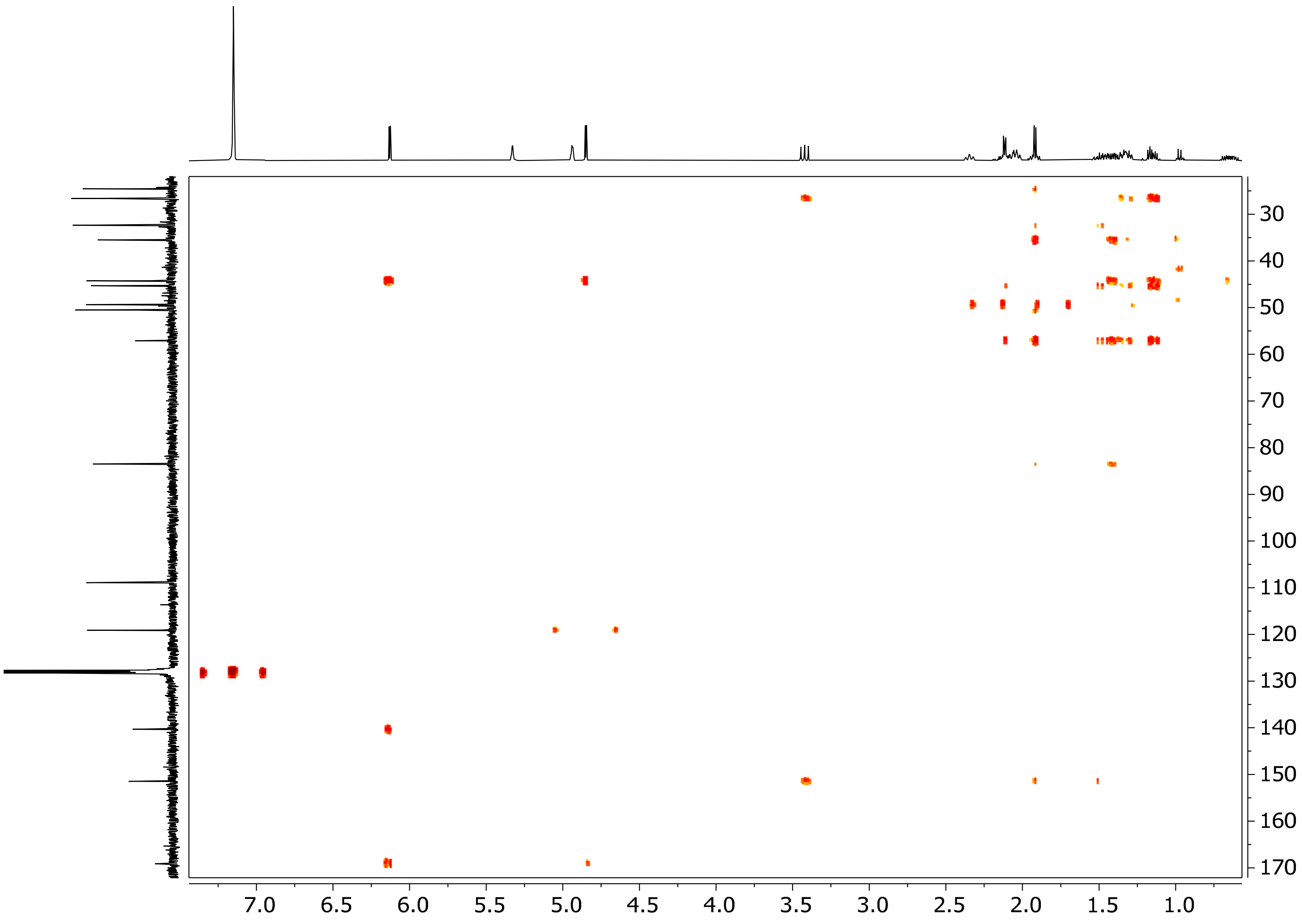

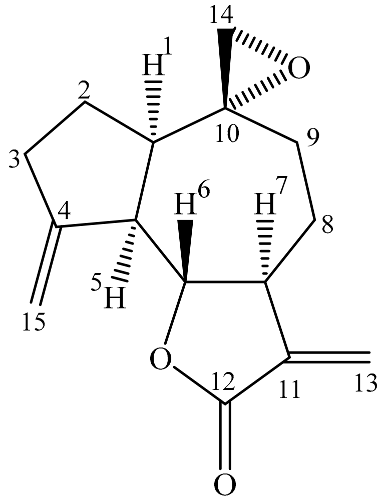

Figure S18: HMBC spectrum (CDCl3) of (3a*S*,6*R*,6a*R*,9a*R*,9b*S*)-3,9-dimethylenedecahydro-2*H*-spiro[azuleno[4,5-b]furan-6,2'-oxiran]-2-one **(3)** (400 MHz).

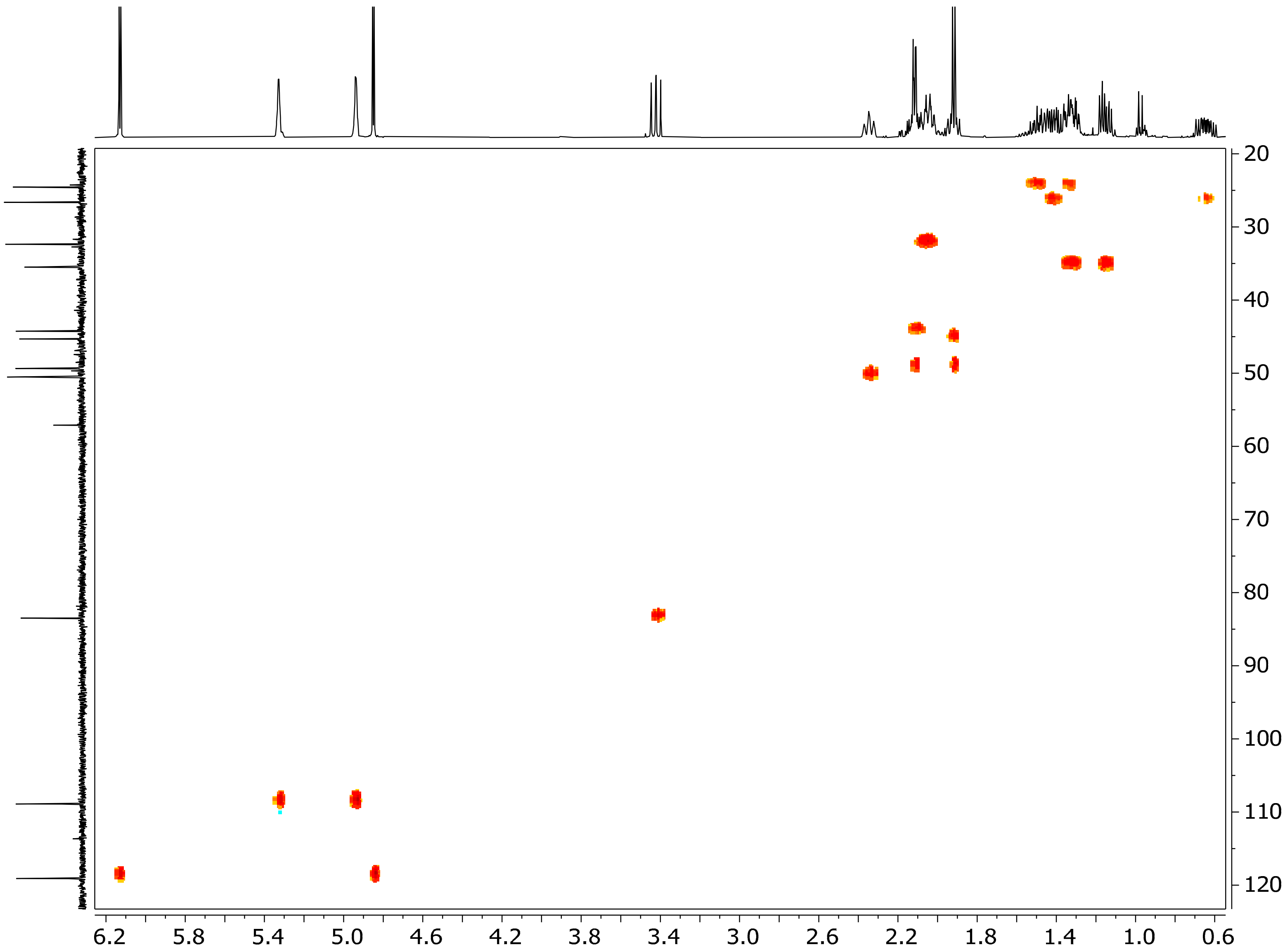

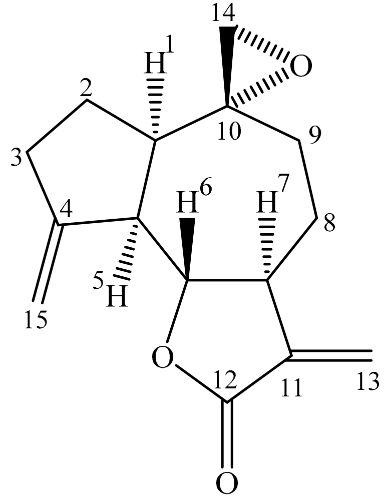

Figure S19: HSQC spectrum ($CDCl_3$) of (3a*S*,6*R*,6a*R*,9a*R*,9b*S*)-3,9-dimethylenedecahydro-2*H*-spiro[azuleno[4,5-b]furan-6,2'-oxiran]-2-one **(3)** (400 MHz).

Table S7: Nuclear magnetic resonance data of (2*R*,3a'*S*,6a'*R*,9'*R*,9a'*S*,9b'*S*)-3'-methyleneoctahydrodispiro[oxirane-2,6'-azuleno[4,5-b]furan-9',2''-oxiran]-2'(3'*H*)-one **(4)**

δ_H	Hydrogen	HMBC	δ_C	Carbon
0.50-0.61	8´ (m)	C2-H3	22.4	2
1.12-1.18	3´, 9´(m)	C8-H6, C8-H9´	27.1	8
1.21-1.30	8 (m)	C3-H15	32.7	3
1.36-1.49	2´, 9 (m)	C9-H8, C9-H8´	38.6	9
1.54-1.65	2, 3 (m)	C5-H7, C5-H3´	43.6	5
1.86-1.91	1, 7 (m)	C7-H8´	44.1	7
2.06-2.17	5, 14´, 15´ (m)	C14-H1	47.3	14
2.35	15´ (d, *J*=4.8Hz)	C1-H9	47.4	1
2.71	14 (dd, *J*=2, 4.4Hz)		48.4	15
3.12	15´(d, *J*=4.4Hz)	C10-H14, C10-H2´, C10-H2, C10-H1	56.2	10
3.63	6 (dd, 9.2, 10Hz)	C4-H15, C4-H15´, C4-H6	64.6	4
4.82	13´ (d, *J*=2.8Hz)	C6-H7, C6-H8´	80.4	6
6.10	13 (d, *J*= 2.8Hz)		119.3	13
		C11-H13	139.5	11
		C12-H13, C12-H13´	168.9	C=O

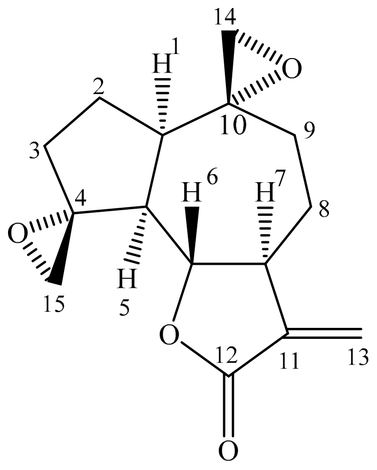

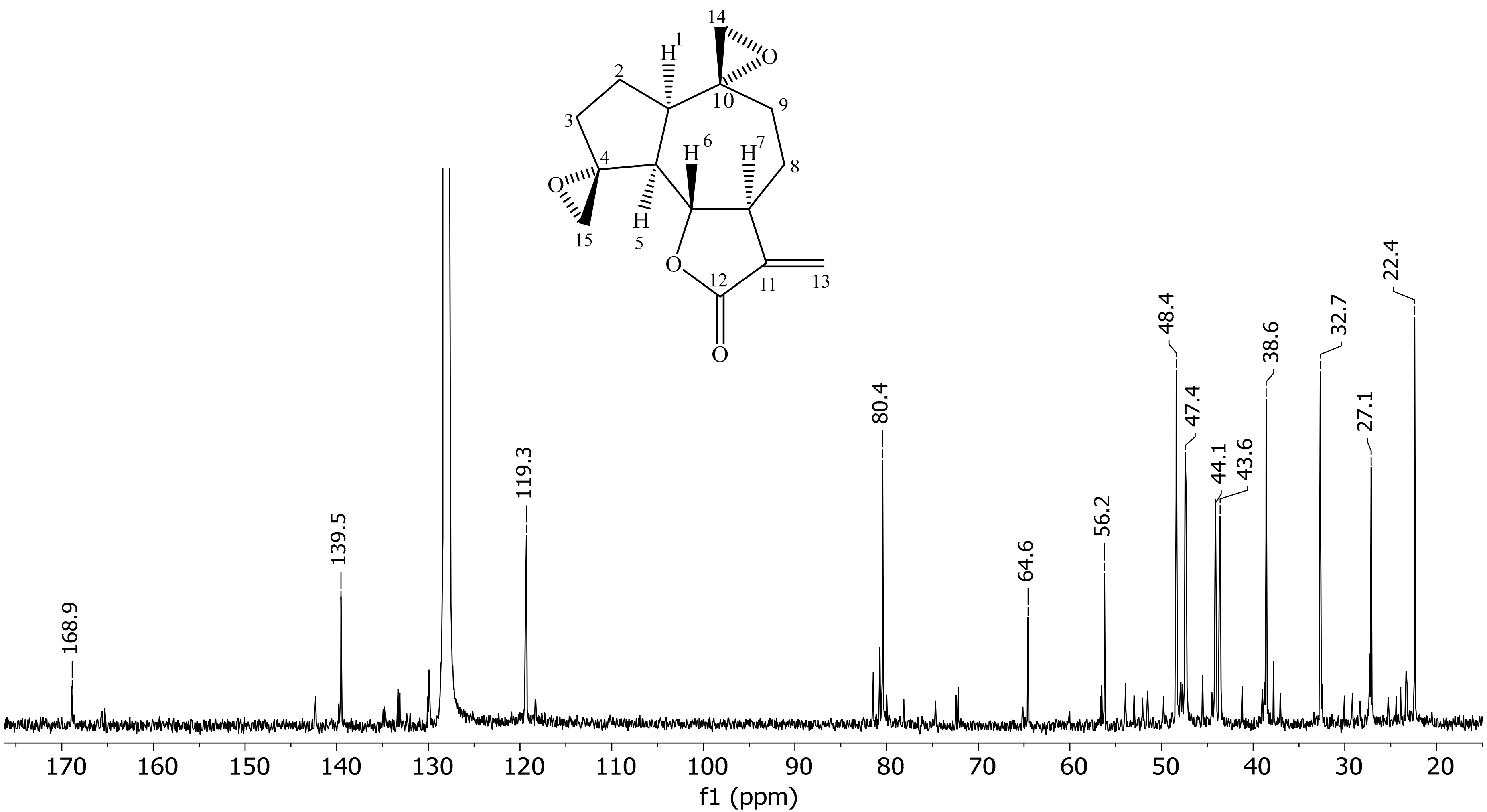

Figure S20: ^{13}C NMR spectrum (C_6D_6) of (2*R*,3a'*S*,6a'*R*,9'*R*,9a'*S*,9b'*S*)-3'-methyleneoctahydrodispiro[oxirane-2,6'-azuleno[4,5-b]furan-9',2''-oxiran]-2'(3'*H*)-one **(4)** (100 MHz).

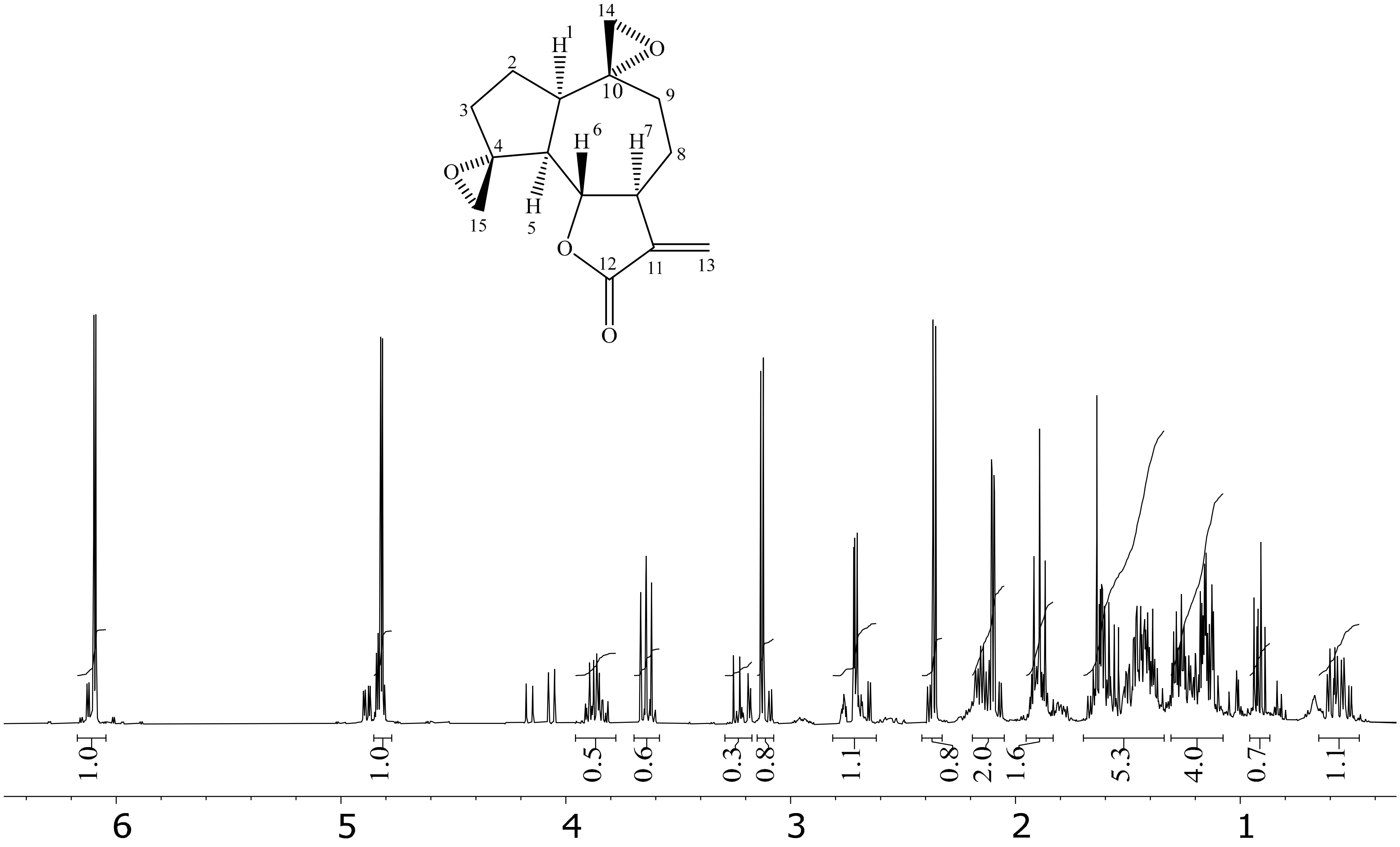

Figure S21: ^{1}H NMR spectrum (C_6D_6) of (2*R*,3a'*S*,6a'*R*,9'*R*,9a'*S*,9b'*S*)-3'-methyleneoctahydrodispiro[oxirane-2,6'-azuleno[4,5-b]furan-9',2''-oxiran]-2'(3'*H*)-one **(4)** (400 MHz).

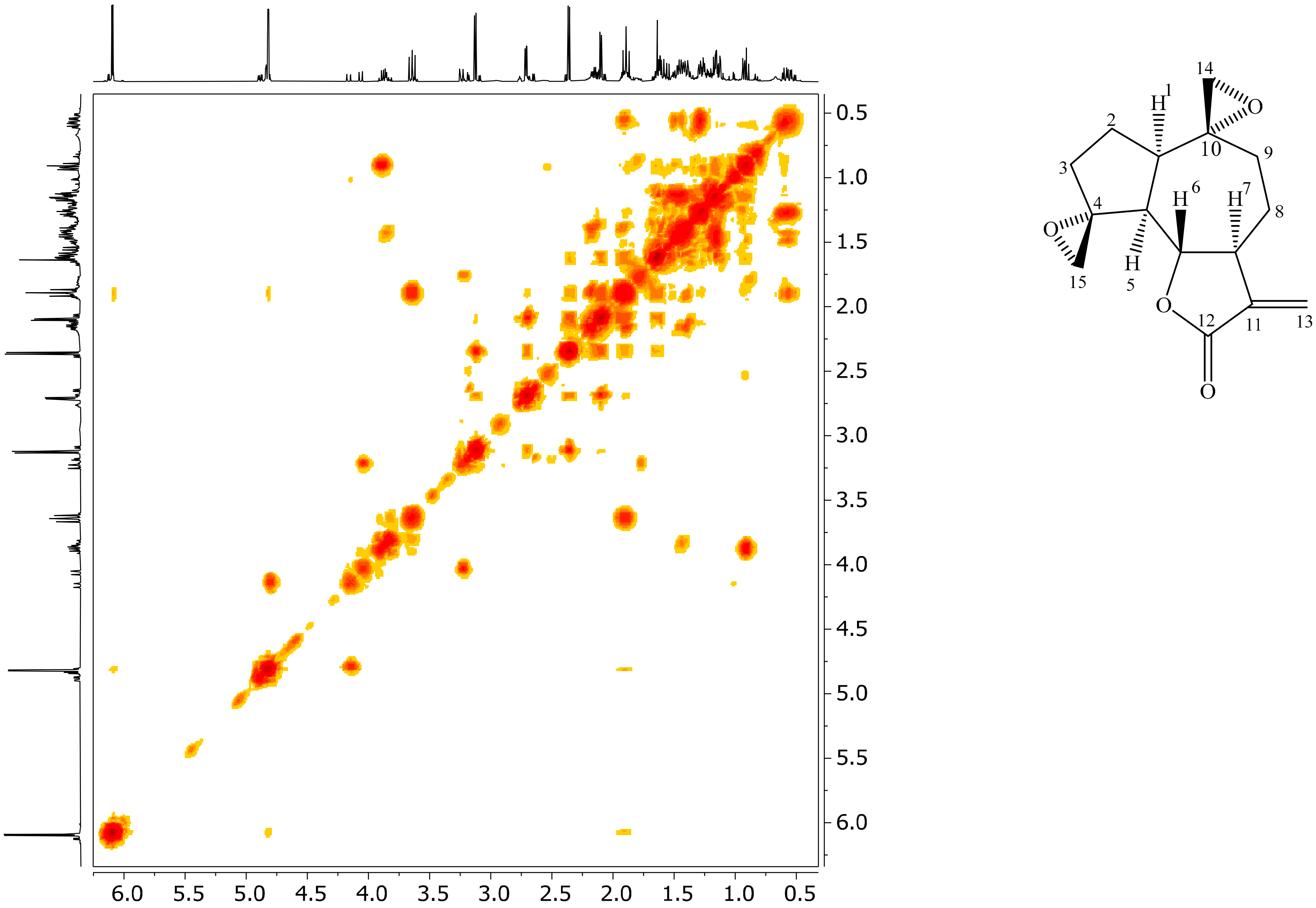

Figure S22: COSY spectrum (C_6D_6) of (2*R*,3a'*S*,6a'*R*,9'*R*,9a'*S*,9b'*S*)-3'-methyleneoctahydrodispiro[oxirane-2,6'-azuleno[4,5-b]furan-9',2''-oxiran]-2'(3'*H*)-one **(4)** (400 MHz).

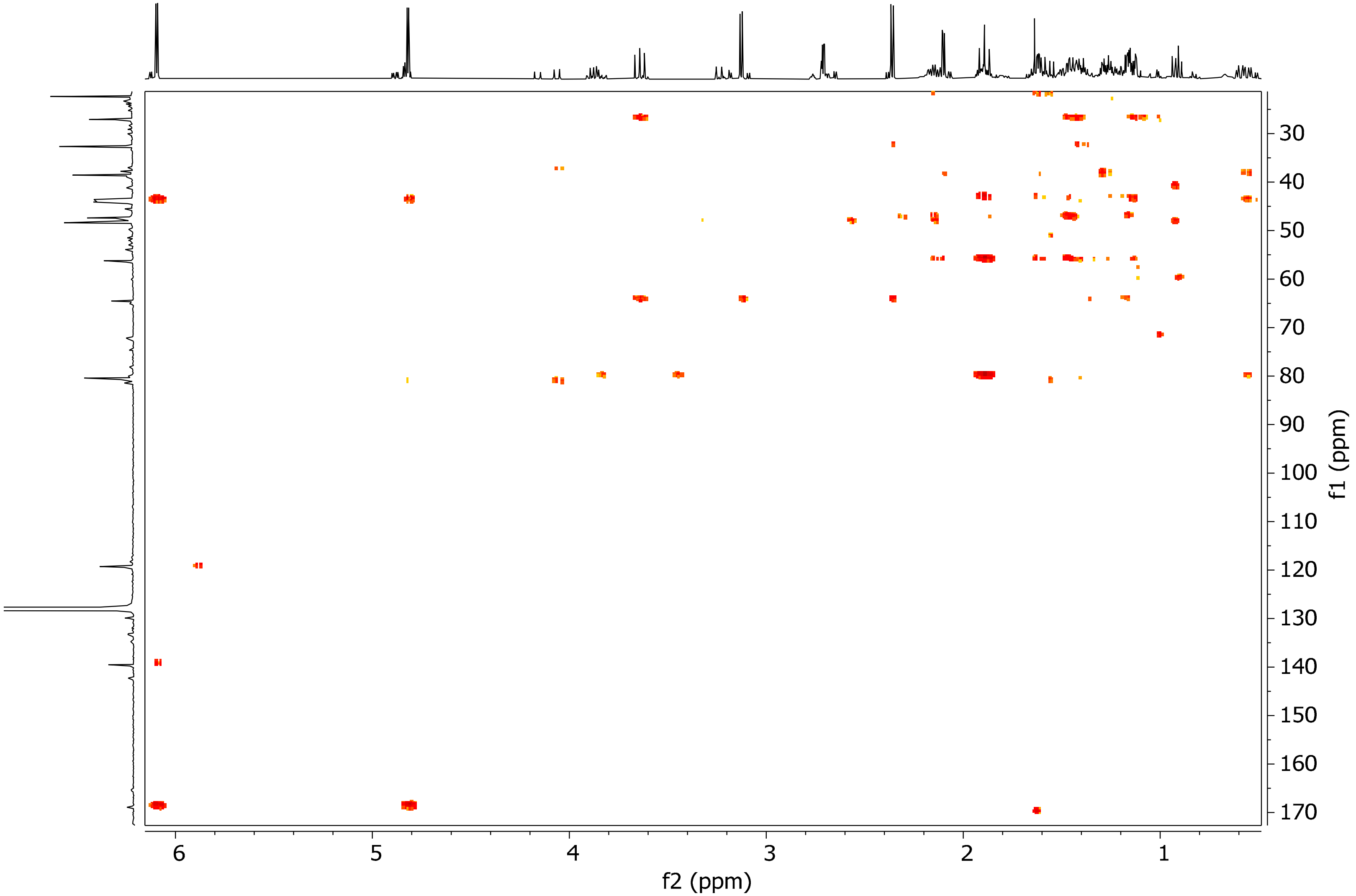

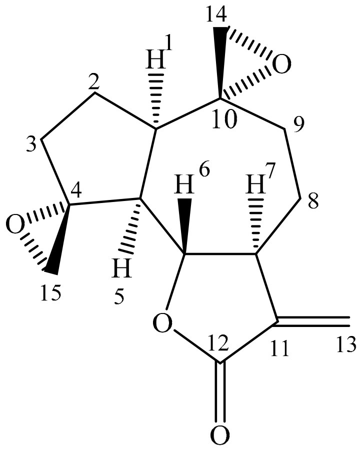

Figure S23: HMBC spectrum (C_6D_6) of (2*R*,3a'*S*,6a'*R*,9'*R*,9a'*S*,9b'*S*)-3'-methyleneoctahydrodispiro[oxirane-2,6'-azuleno[4,5-b]furan-9',2''-oxiran]-2'(3'*H*)-one **(4)** (400 MHz).

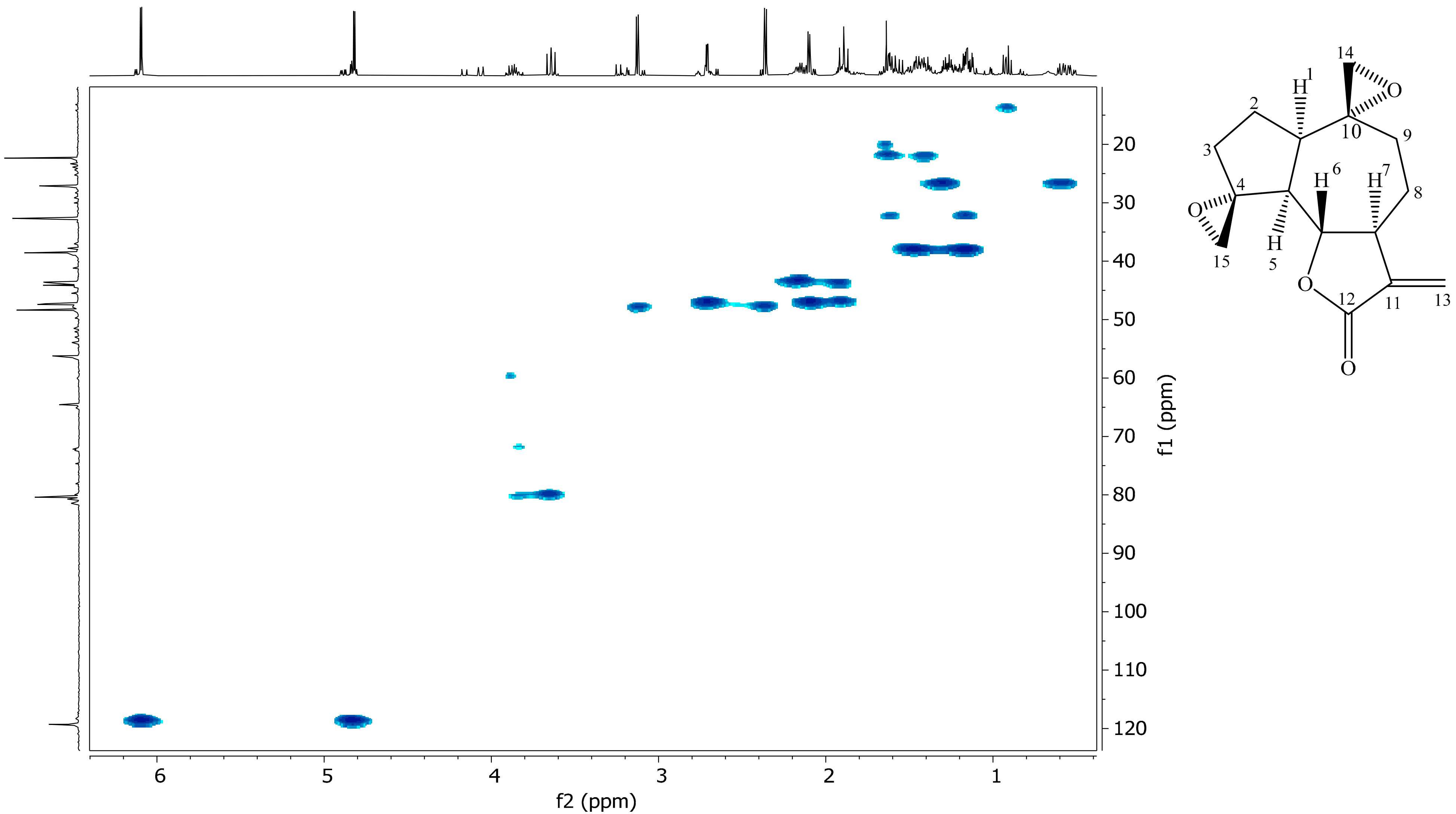

Figure S24: HSQC spectrum (C_6D_6) of (2*R*,3a'*S*,6a'*R*,9'*R*,9a'*S*,9b'*S*)-3'-methyleneoctahydrodispiro[oxirane-2,6'-azuleno[4,5-b]furan-9',2''-oxiran]-2'(3'*H*)-one **(4)** 400 MHz).

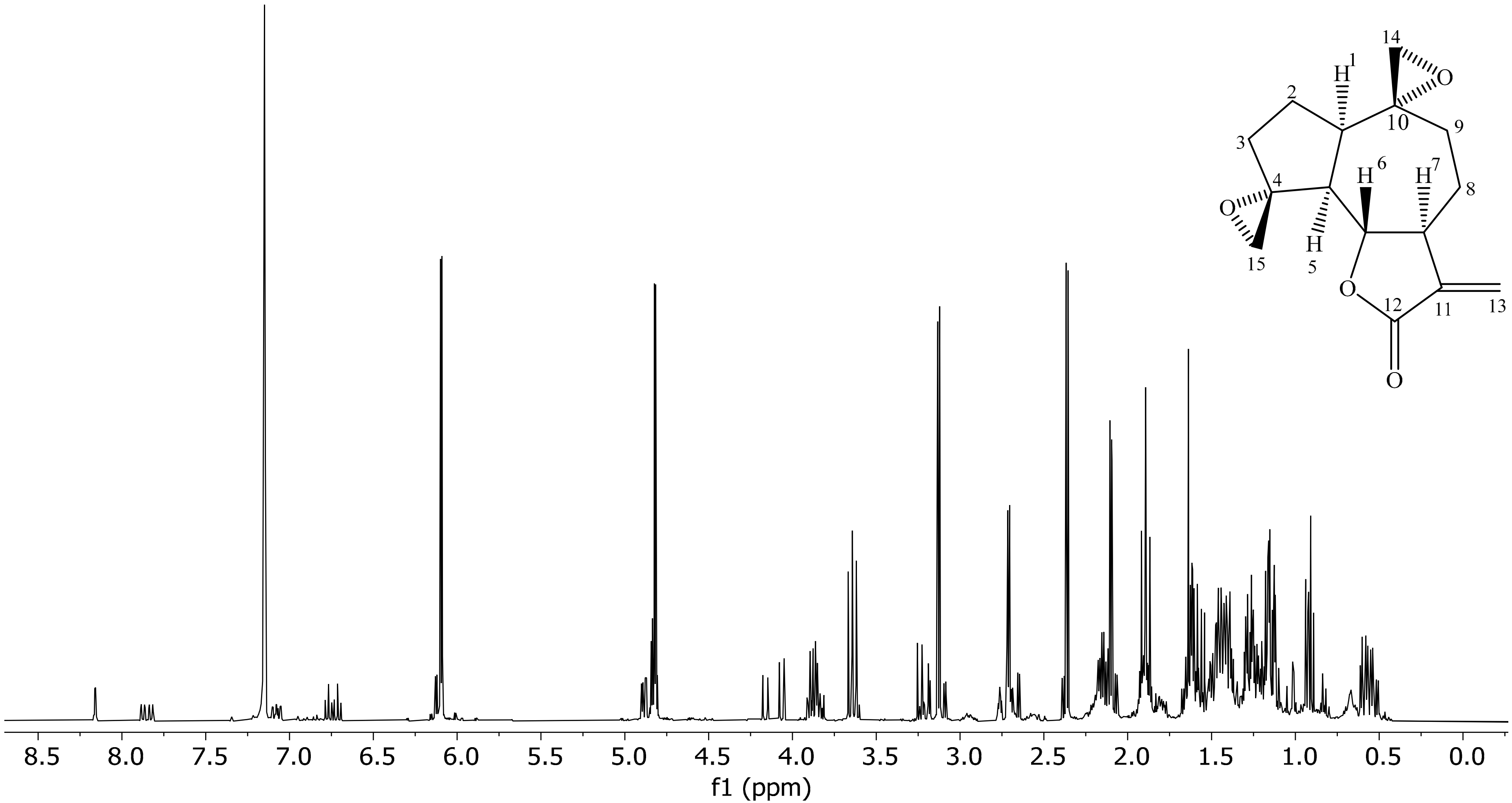

Figure S25: ^{1}H NMR spectrum (C_6D_6) of (2*R*,3a'*S*,6a'*R*,9'*R*,9a'*S*,9b'*S*)-3'-methyleneoctahydrodispiro[oxirane-2,6'-azuleno[4,5-b]furan-9',2''-oxiran]-2'(3'*H*)-one **(4)** (400 MHz).

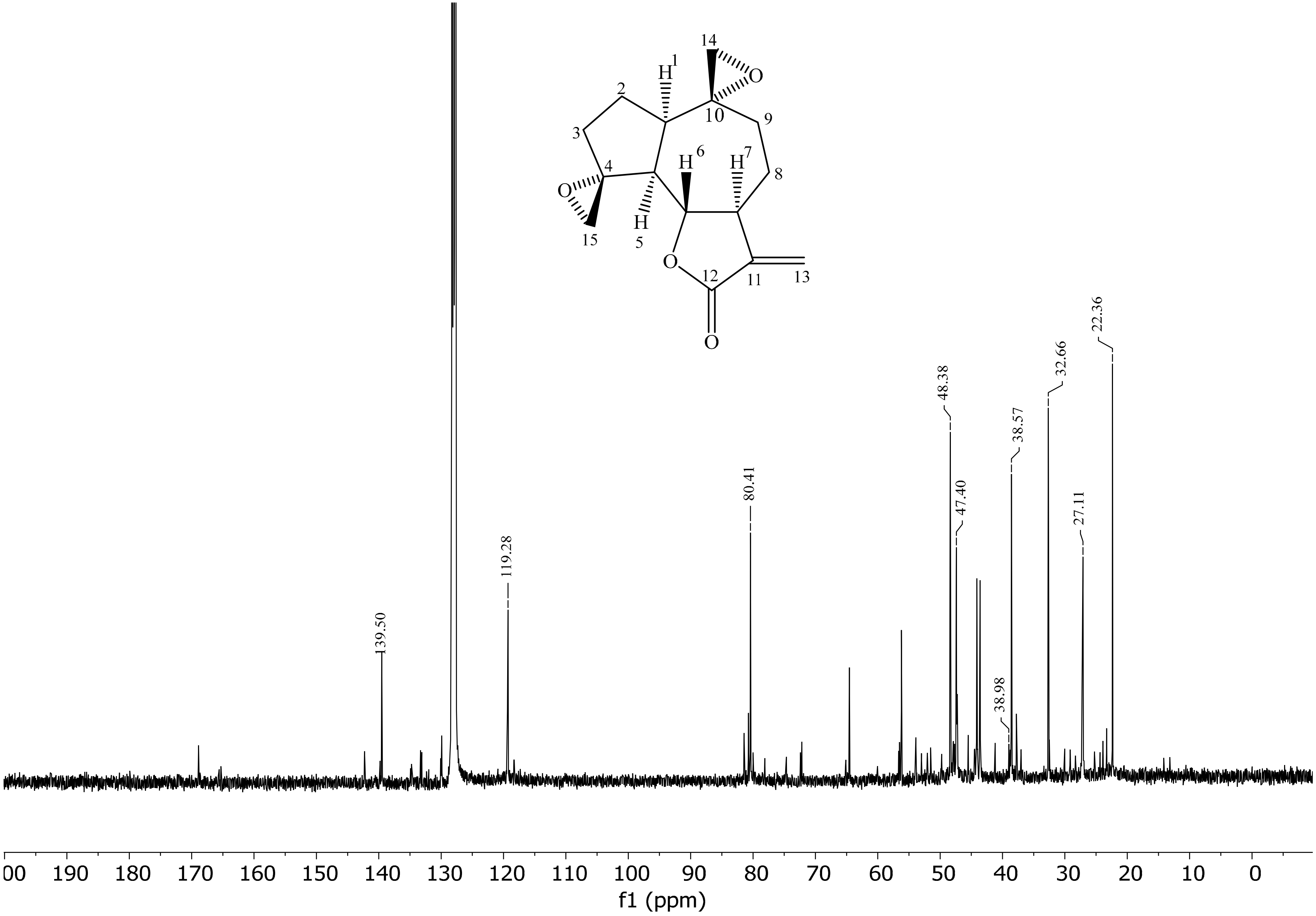

Figure S26: ^{13}C NMR spectrum (C_6D_6) of (2*R*,3a'*S*,6a'*R*,9'*R*,9a'*S*,9b'*S*)-3'-methyleneoctahydrodispiro[oxirane-2,6'-azuleno[4,5-b]furan-9',2''-oxiran]-2'(3'*H*)-one **(4)** (100 MHz).

Table S8: Nuclear magnetic resonance data of (3a*S*,6a*R*,9*S*,9a*S*,9b*S*)-9-hydroxy-9-(hydroxymethyl)-3,6-dimethylenedecahydroazuleno[4,5-b]furan-2(3*H*)-one (**5**)

δ_H	Hydrogen	COSY	HMBC	δ_C	Carbon
0.71-0.81	8´	8´x8, 8´x9, 8´x7	C2-H3, C2-H3´	29.4	2
1.42-1.66	2´, 3´, 8, 9´	2´x3, 2´x2, 3´x3, 8x9, 9´x9	C8 - H6, C8 - H9´, H2'-C3	31.0	8
1.74-1.85	2, 3, 9	9x8, 9x9´, 9x8´	-	33.4	3
1.96	5	5x6, 5x1	C9-H14, C9 - H14´, C9-H8´	38.5	9
2.24	1	1x5, 1x2, 1x2´	C7 - H13, C7 - H13´, C7 - H8, C7 - H8´, C7- H7	44.3	7
2.36	15´	15´x15	C1 - H14, C1 - H14´	46.3	1
3.01	15	15x15	C15 - H15, C15 - H15´	47.6	15
3.87	6	6x7, 6x5	C5 - H3, C5 - H7, C5 - H3´, C5 - H15, C5 - H15´	49.0	5
4.77	14´	14´x14	C4 - H2´, C4-H15, C4-H15´, C4-H6	64.3	4
4.83	13´	13´x13	C6 - H5, C6 - H8, C6 - H8´	81.0	6
4.88	14	14x14´		112.5	13
6.07	13	13x13'	C14-H1, C14 - H9´	118.9	14
			C11-H13	139.9	11
			C10-H1, C10-H5, C10-H2'	149.3	10
			C12 - H13, C12 - H13´	169.1	C12

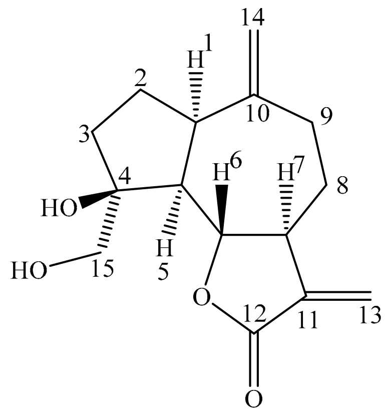

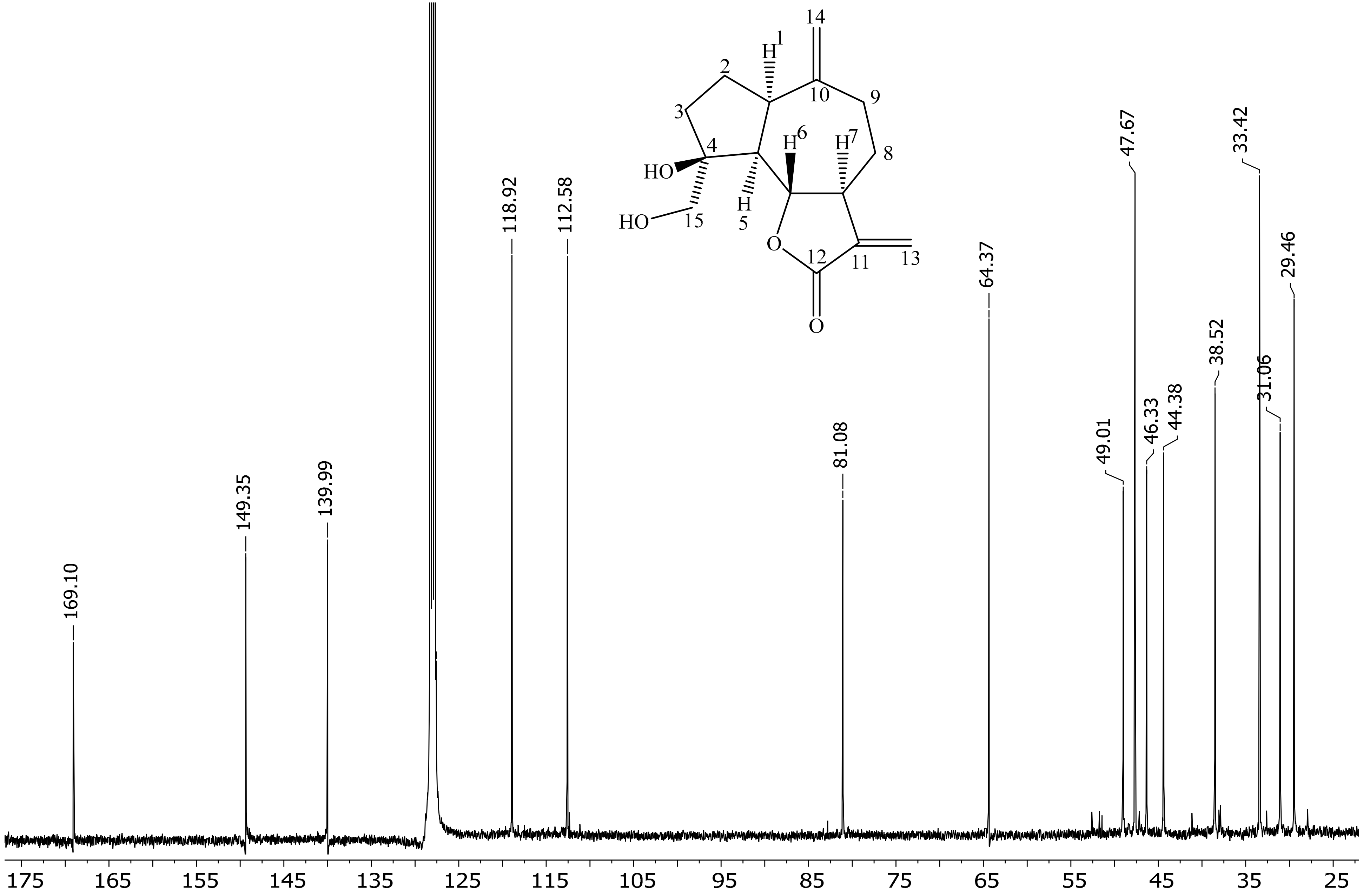

Figure S27: ^{13}C NMR spectrum (C_6D_6) of (3a*S*,6a*R*,9*S*,9a*S*,9b*S*)-9-hydroxy-9-(hydroxymethyl)-3,6-dimethylenedecahydroazuleno[4,5-b]furan-2(3*H*)-one **(5)** (100 MHz).

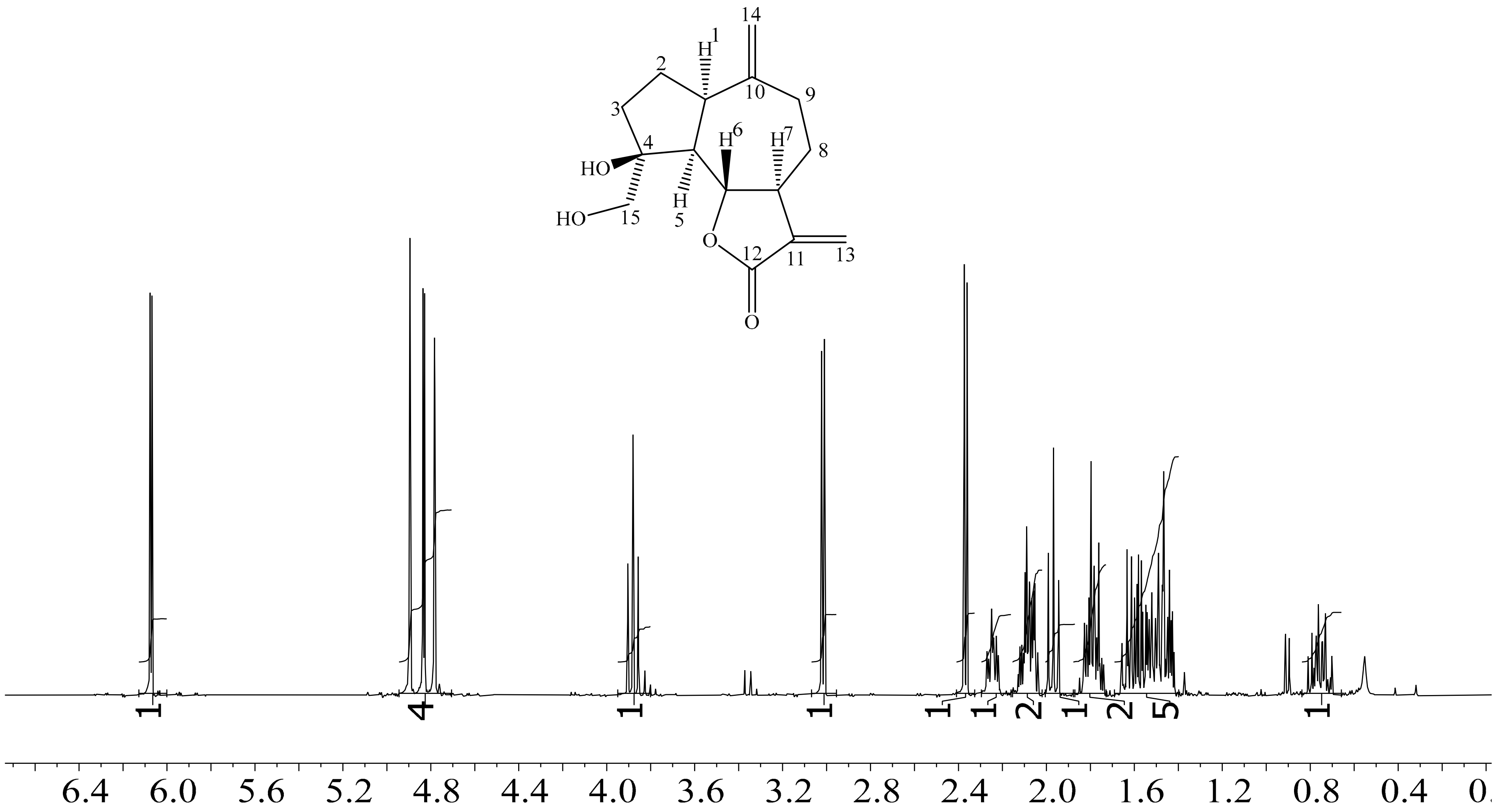

Figure S28: ^{1}H NMR spectrum (C_6D_6) of (3a*S*,6a*R*,9*S*,9a*S*,9b*S*)-9-hydroxy-9-(hydroxymethyl)-3,6-dimethylenedecahydroazuleno[4,5-b]furan-2(3*H*)-one **(5)** (400 MHz).

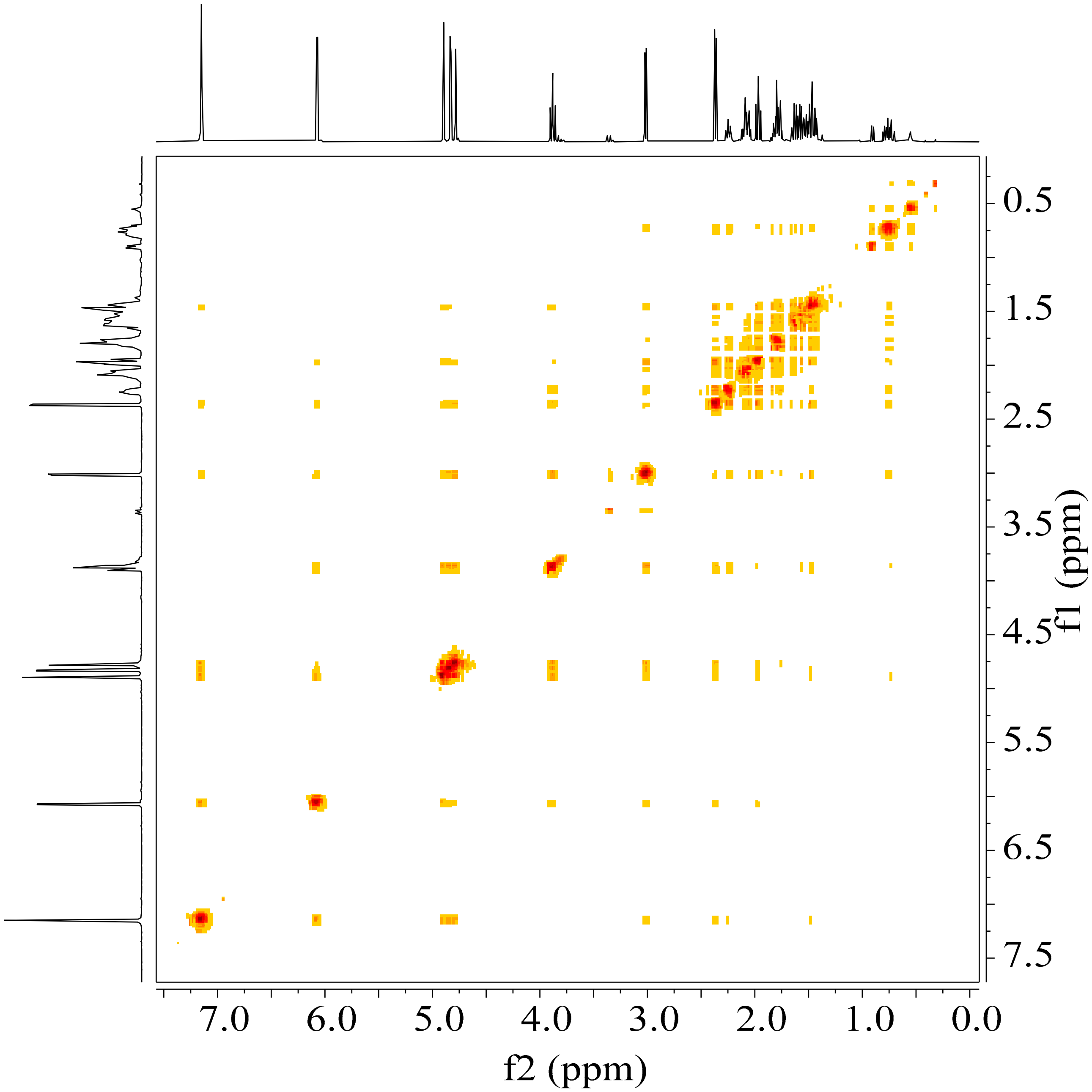

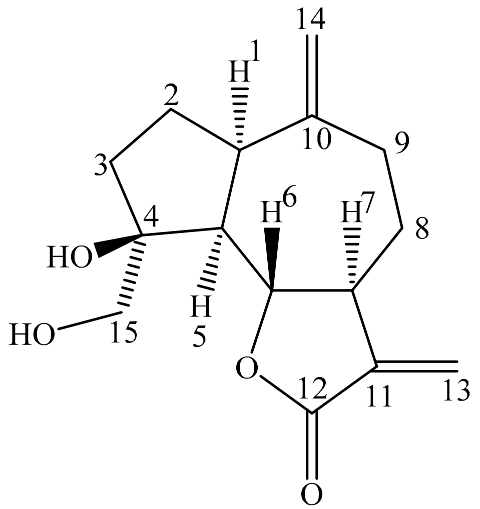

Figure S29: COSY spectrum (C_6D_6) of (3a*S*,6a*R*,9*S*,9a*S*,9b*S*)-9-hydroxy-9-(hydroxymethyl)-3,6-dimethylenedecahydroazuleno[4,5-b]furan-2(3*H*)-one **(5)** (400 MHz).

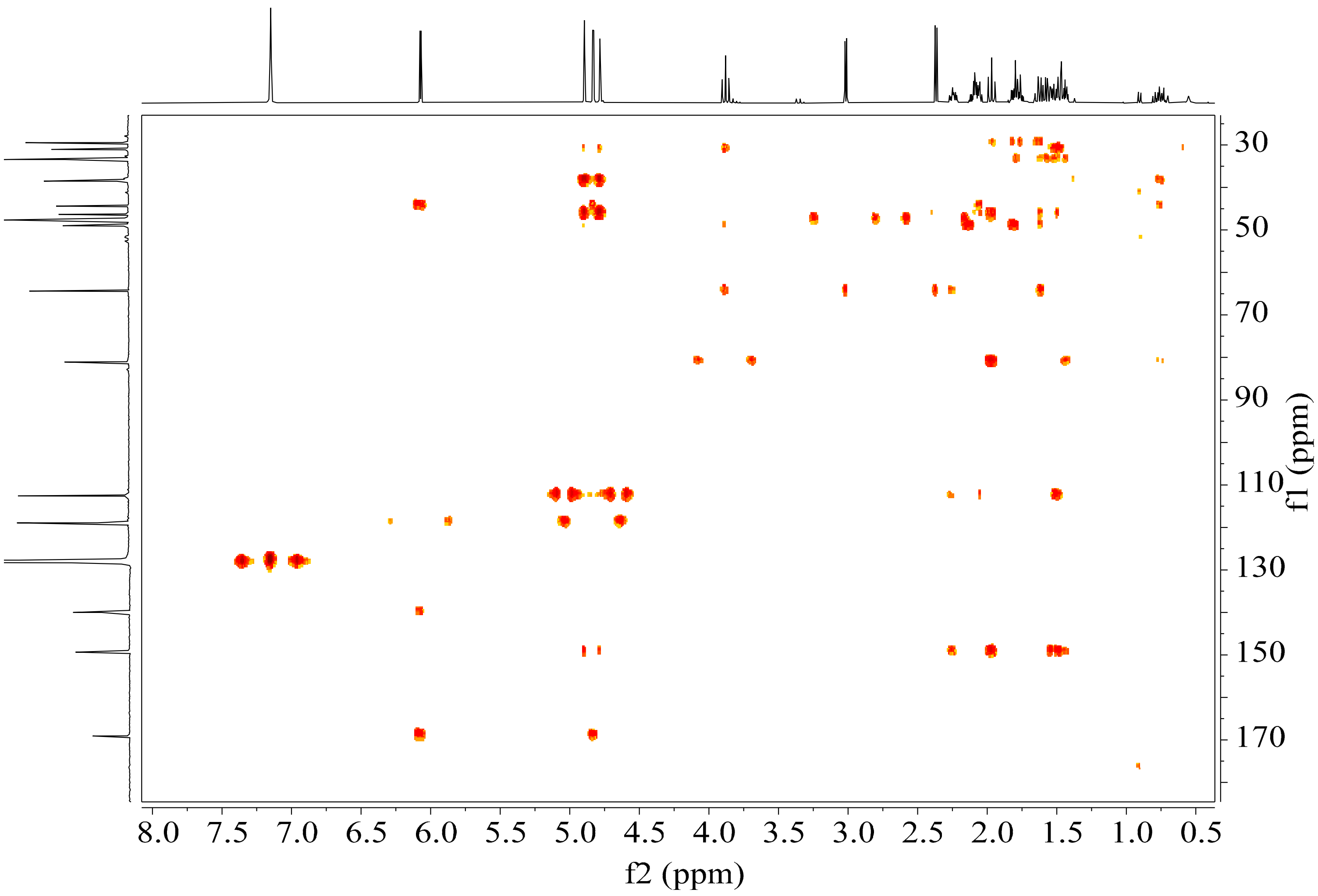

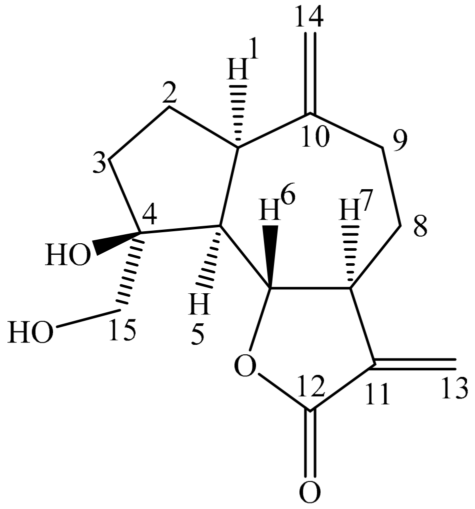

Figure S30: HMBC spectrum (C_6D_6) of (3a*S*,6a*R*,9*S*,9a*S*,9b*S*)-9-hydroxy-9-(hydroxymethyl)-3,6-dimethylenedecahydroazuleno[4,5-b]furan-2(3*H*)-one **(5)** (400 MHz).

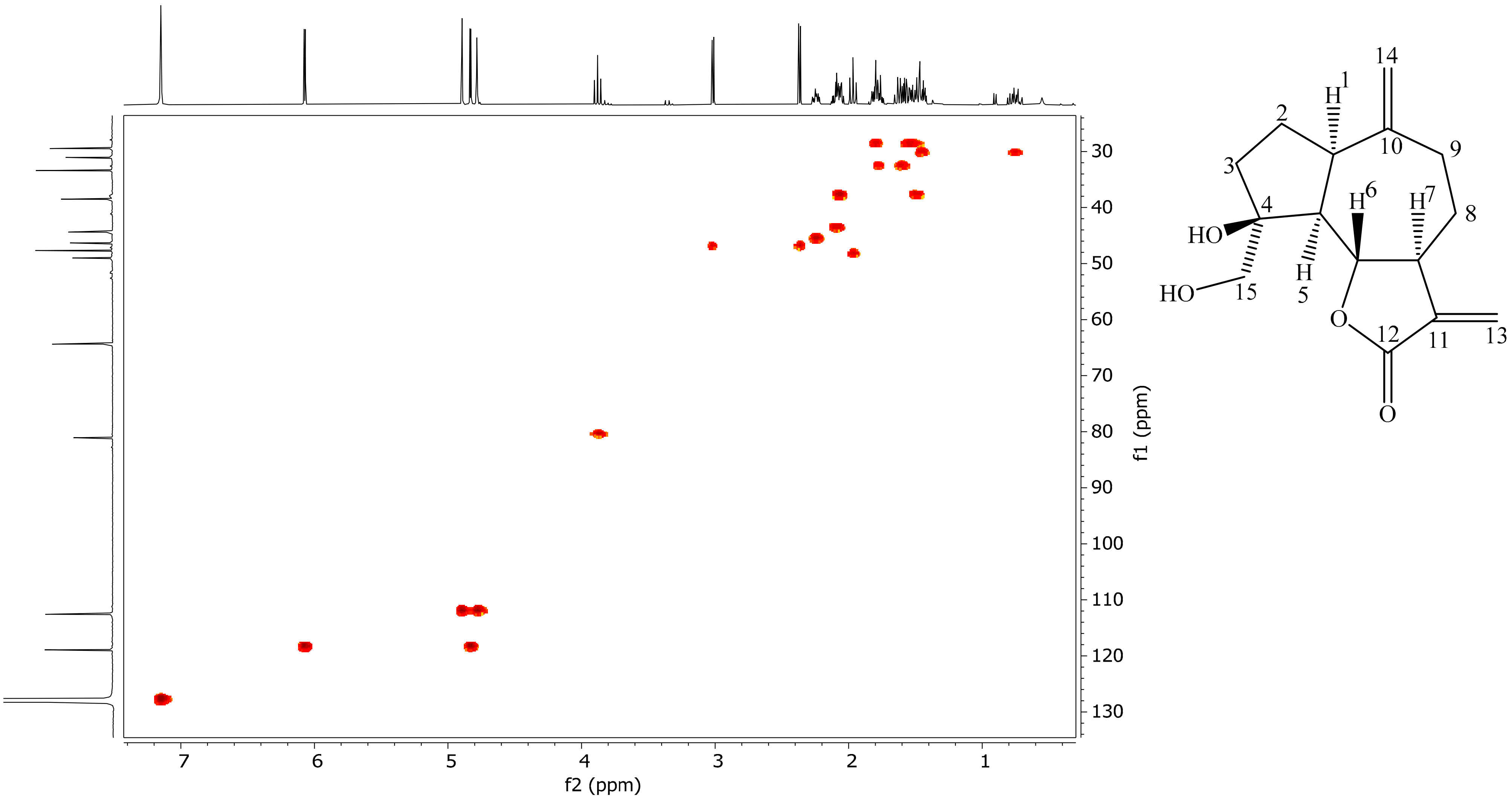

Figure S31: HSQC spectrum (C_6D_6) of (3a*S*,6a*R*,9*S*,9a*S*,9b*S*)-9-hydroxy-9-(hydroxymethyl)-3,6-dimethylenedecahydroazuleno[4,5-b]furan-2(3*H*)-one **(5)** (400 MHz).

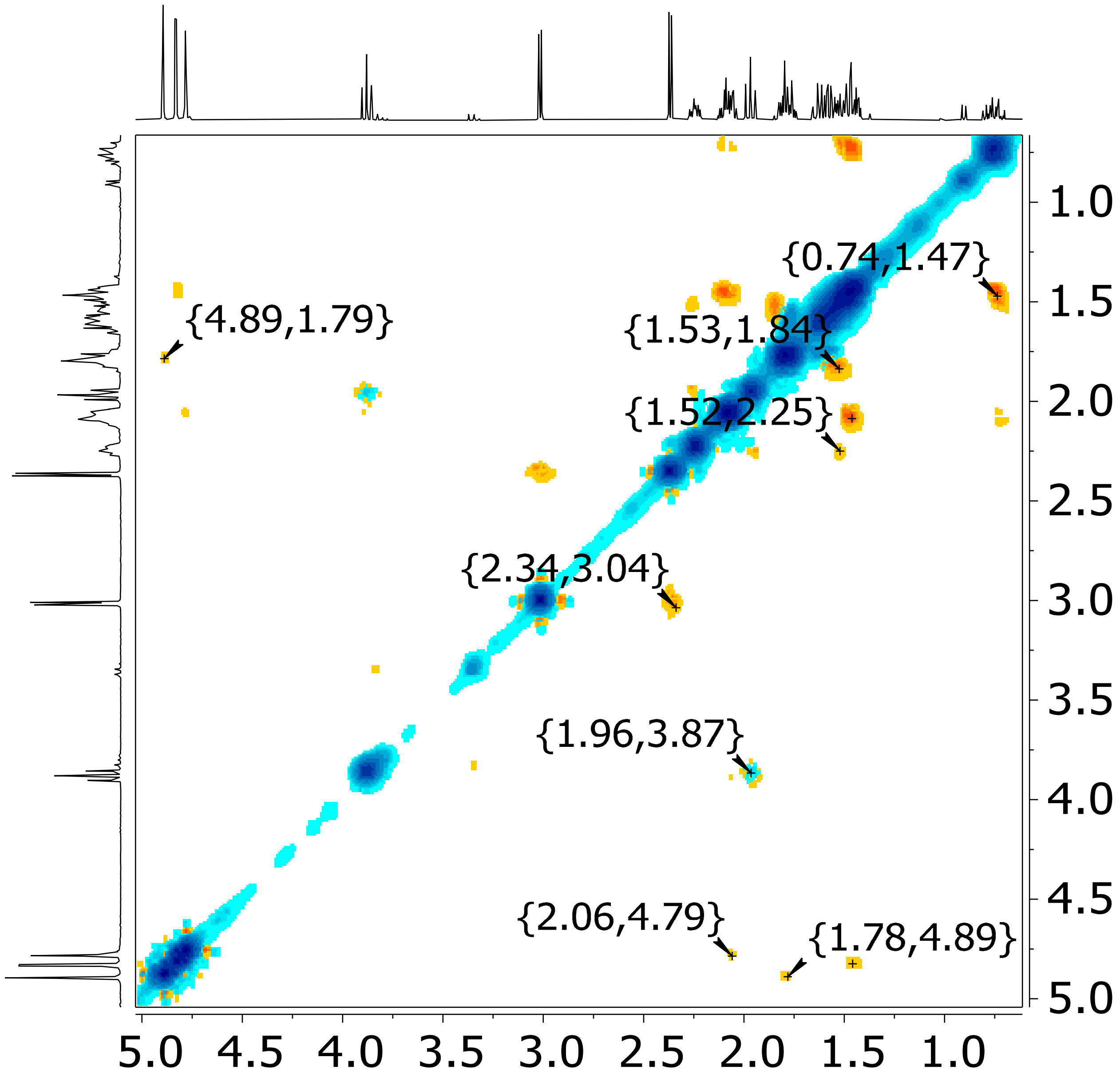

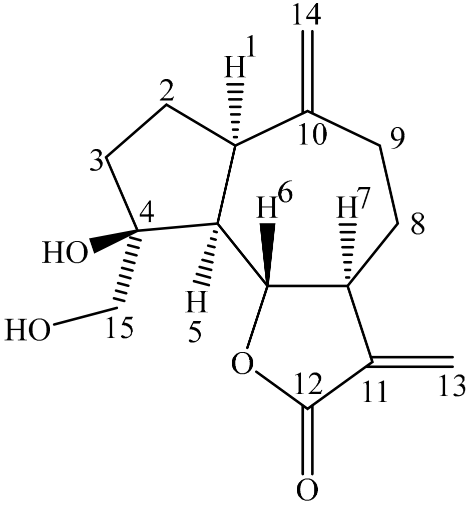

Figure S31: NOESY spectrum (C_6D_6) of (3a*S*,6a*R*,9*S*,9a*S*,9b*S*)-9-hydroxy-9-(hydroxymethyl)-3,6-dimethylenedecahydroazuleno[4,5-b]furan-2(3*H*)-one **(5)** (400 MHz).

Table S9: Results of bioassays on *Allium cepa* (onion) seed germination.

	BIOACTIVITY									
	GERMINATION			ROOT			SHOOT			
	% from control	S. D.	α	% from control	S. D.	α	% from control	S. D.	α	
100 *μM*	4	15	n.s.	-78	4	a	-73	4	a	Reference Compound (Logran)
60 *μM*	-14	17	n.s.	-76	3	a	-73	2	a	
30 *μM*	-11	4	n.s.	-73	4	a	-63	3	a	
15 *μM*	-13	9	n.s.	-76	2	a	-59	3	a	
5 *μM*	-5	14	n.s.	-64	3	a	-53	4	a	
100 *μM*	-16	8	n.s.	-43	4	a	-12	8	n.s.	Compound 2
60 *μM*	0	5	n.s.	-18	6	b	7	2	n.s.	
30 *μM*	-7	13	n.s.	-8	15	n.s.	4	8	n.s.	
15 *μM*	-23	13	n.s.	8	11	n.s.	16	11	n.s.	
5 *μM*	5	18	n.s.	-1	18	n.s.	10	4	n.s.	
100 *μM*	-4	6	n.s.	-80	2	a	-51	7	a	Compound 4
60 *μM*	-30	8	b	-77	4	a	-60	6	a	
30 *μM*	-14	32	n.s.	-6	12	n.s.	-4	6	n.s.	
15 *μM*	-61	12	a	-33	14	b	-26	14	n.s.	
5 *μM*	2	18	n.s.	-17	22	n.s.	-18	24	n.s.	
100 *μM*	18	16	n.s.	-52	9	a	-34	9	a	Compound 5
60 *μM*	-30	20	n.s.	-20	12	n.s.	-13	16	n.s.	
30 *μM*	9	25	n.s.	-19	16	n.s.	5	15	n.s.	
15 *μM*	20	15	n.s.	-15	5	n.s.	1	6	n.s.	
5 *μM*	-7	5	n.s.	-11	14	n.s.	-2	5	n.s.	
100 *μM*	-7	9	n.s.	-47	7	a	-40	16	b	Compound 6
60 *μM*	-14	9	n.s.	-23	14	n.s.	-4	6	n.s.	
30 *μM*	-14	9	n.s.	12	11	n.s.	9	7	n.s.	
15 *μM*	-11	19	n.s.	4	32	n.s.	2	13	n.s.	
5 *μM*	-18	11	n.s.	-5	29	n.s.	-3	19	n.s.	
100 *μM*	-18	23	n.s.	-57	6	a	-29	4	b	Compound 7
60 *μM*	-7	20	n.s.	-14	6	n.s.	-11	5	n.s.	
30 *μM*	-13	18	n.s.	-25	20	n.s.	-19	9	n.s.	
15 *μM*	4	15	n.s.	-41	8	a	-20	12	n.s.	
5 *μM*	-18	6	n.s.	1	26	n.s.	-5	13	n.s.	

S.D. = Standard Deviation
α = Confidence Level

a = p < 0,01
b = 0,01 < p < 0,05
n.s. = p > 0,05 (not significant)

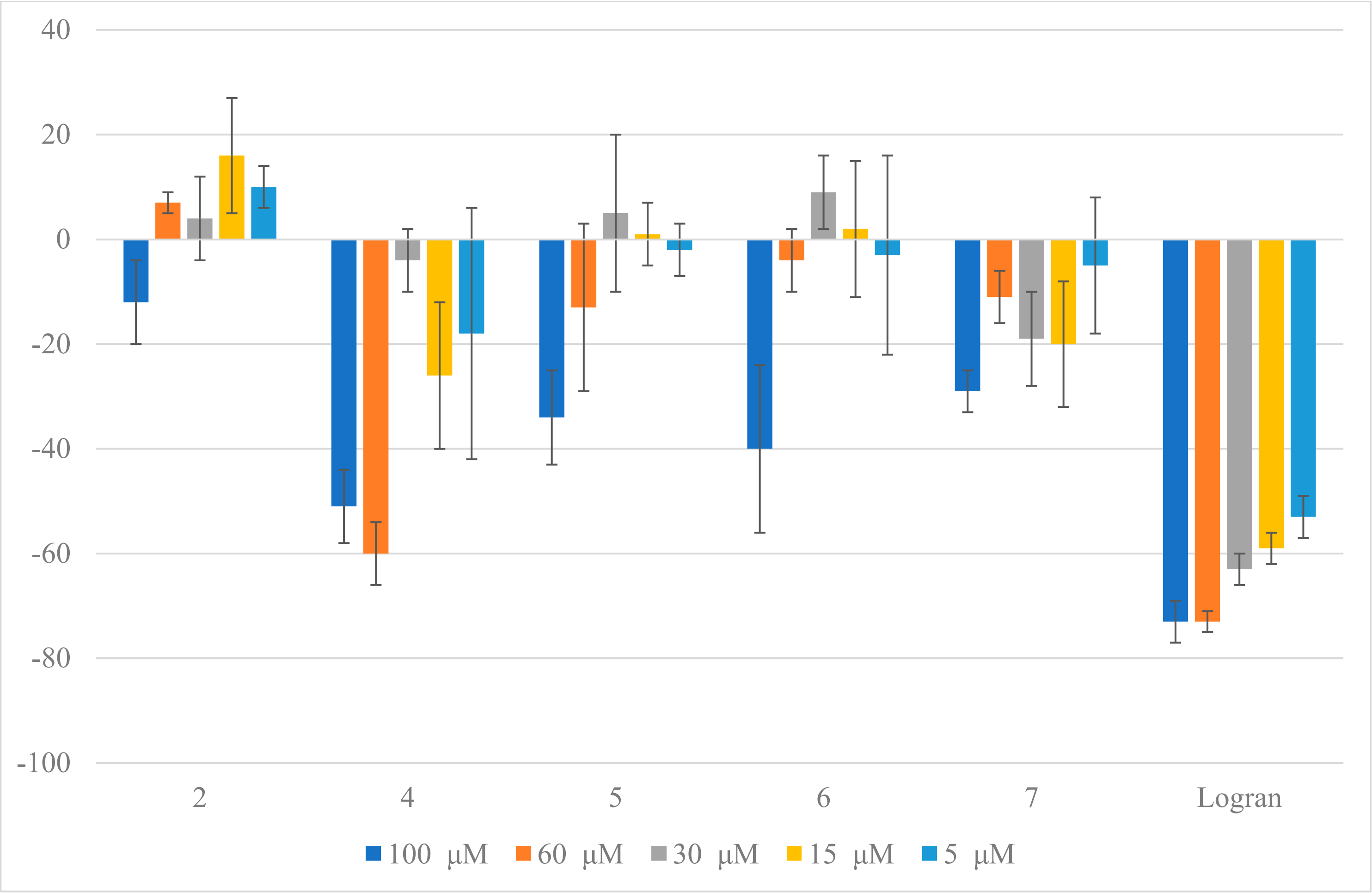

Figure S32: Effect of compounds 2 and 4-7 on the shoot length of *Allium cepa*. Shoot length of seedlings from *A. cepa* (onion) seeds exposed to aqueous 0.1% (v/v) DMSO solutions of compounds at different concentrations. Controls consisted of deionized water with the same concentration of DMSO. Values are expressed as percentage difference from the negative control: shoot length (%) = [(length – length of negative control)/length of negative control]x100. Error bars represent the standard deviation.

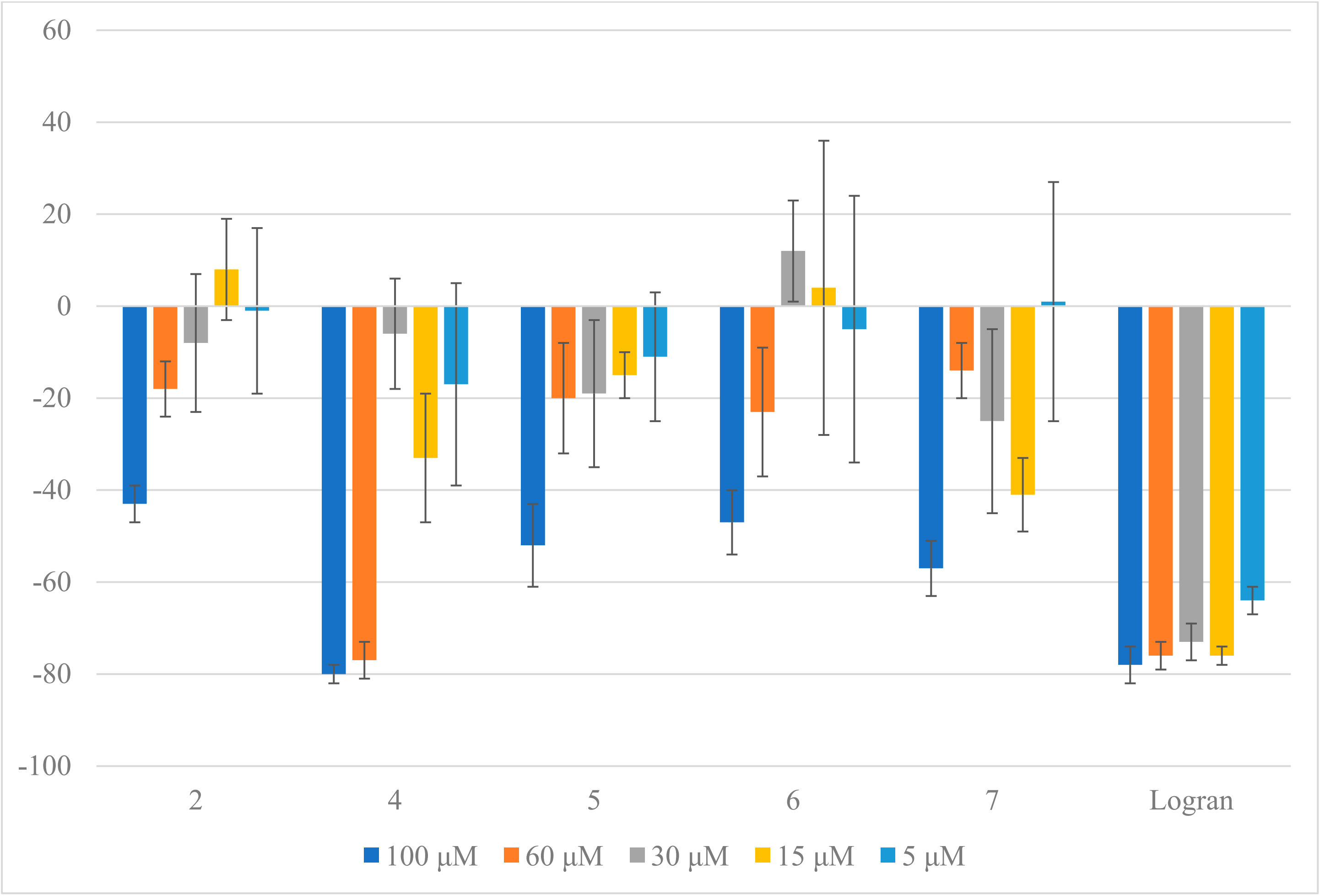

Figure S33: Effect of compounds 2 and 4-7 on the radicle length of *Allium cepa*. Radicle length of seedlings from *A. cepa* (onion) seeds exposed to aqueous 0.1% (v/v) DMSO solutions of compounds at different concentrations. Controls consisted of deionized water with the same concentration of DMSO. Values are expressed as percentage difference from the negative control: shoot length (%) = [(length – length of negative control)/length of negative control]x100. Error bars represent the standard deviation.

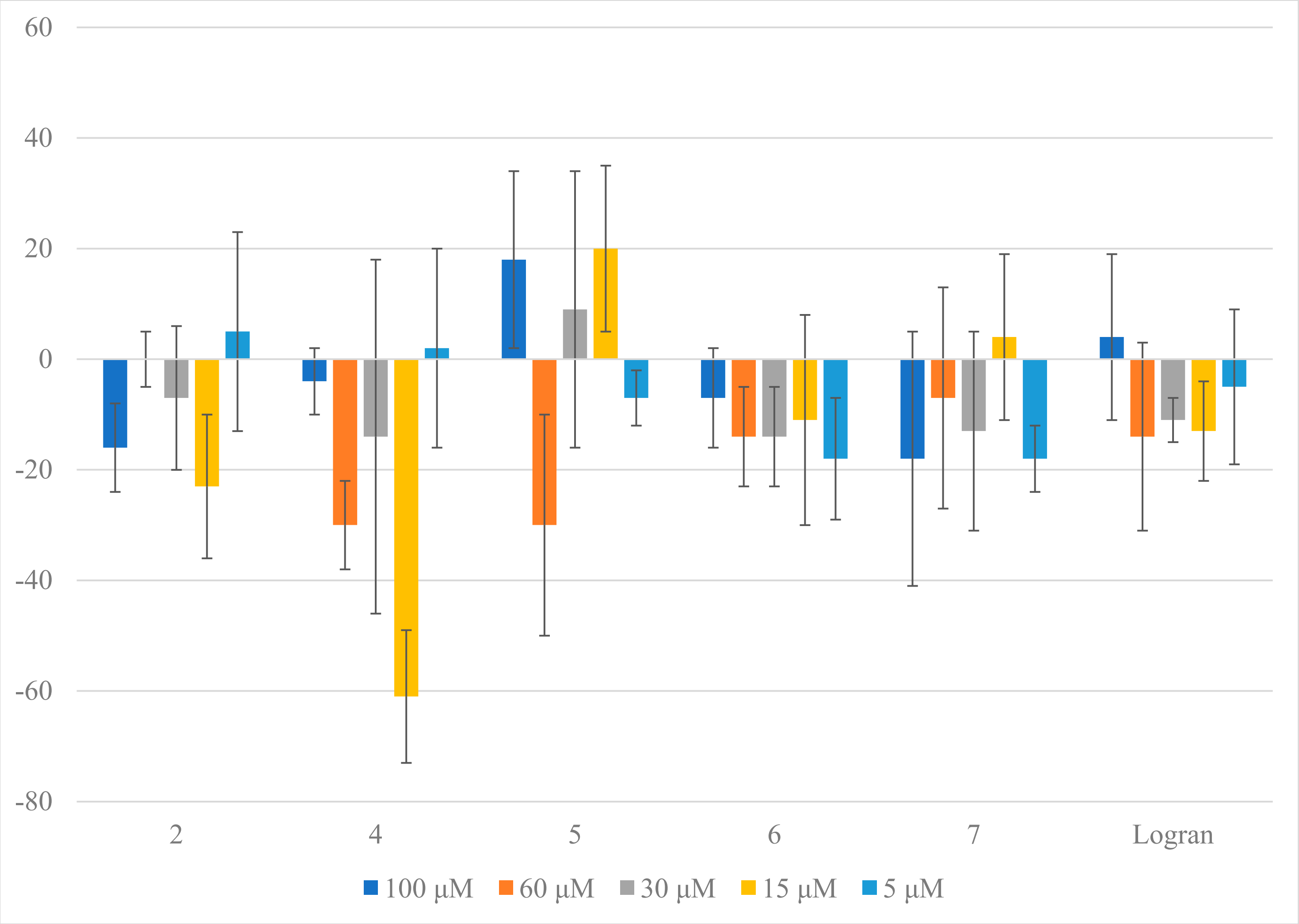

Figure S34: Effect of compounds 2 and 4-7 on the germination of *Allium cepa*. *A. cepa* (onion) seeds were exposed to aqueous 0.1% (v/v) DMSO solutions of compounds at different concentrations. Controls consisted of deionized water with the same concentration of DMSO. Values are expressed as percentage difference from the negative control: shoot length (%) = [(length – length of negative control)/length of negative control]x100. Error bars represent the standard deviation.

Table S10: Results of bioassays on *Lepidium sativum* (garden cress) seed germination.

	BIOACTIVITY									
	GERMINATION			ROOT			SHOOT			
	% from control	S. D.	α	% from control	S. D.	α	% from control	S. D.	α	
100 µM	-3	6	n.s.	-35	14	b	-35	7	a	Reference Compound (Logran)
60 µM	-1	9	n.s.	-13	3	n.s.	-18	2	a	
30 µM	-13	6	b	9	6	n.s.	-5	8	n.s.	
15 µM	1	4	n.s.	57	9	a	7	3	n.s.	
5 µM	-7	10	n.s.	49	31	n.s.	3	10	n.s.	
100 µM	1	4	n.s.	-20	8	n.s.	-5	6	n.s.	Compound 2
60 µM	-4	2	n.s.	75	14	a	11	3	b	
30 µM	5	0	n.s.	114	17	a	11	4	b	
15 µM	0	4	n.s.	123	19	a	19	6	a	
5 µM	-3	3	n.s.	118	43	b	19	6	a	
100 µM	-25	14	b	-88	3	a	-80	1	a	Compound 4
60 µM	-7	7	n.s.	95	25	a	0	2	n.s.	
30 µM	-7	2	b	122	13	a	0	6	n.s.	
15 µM	-7	2	b	138	20	a	8	7	n.s.	
5 µM	-5	7	n.s.	109	26	a	9	3	n.s.	
100 µM	-3	6	n.s.	-37	10	b	-10	6	n.s.	Compound 5
60 µM	-3	3	n.s.	51	19	b	1	5	n.s.	
30 µM	-5	6	n.s.	52	35	n.s.	9	4	n.s.	
15 µM	-13	3	a	66	30	b	8	7	n.s.	
5 µM	-3	8	n.s.	61	16	a	5	5	n.s.	
100 µM	0	4	n.s.	-23	12	n.s.	2	9	n.s.	Compound 6
60 µM	-1	6	n.s.	92	31	a	6	6	n.s.	
30 µM	-3	5	n.s.	122	27	a	1	12	n.s.	
15 µM	-1	7	n.s.	130	28	a	-4	3	n.s.	
5 µM	-4	4	n.s.	91	19	a	5	6	n.s.	
100 µM	0	4	n.s.	192	11	a	20	3	a	Compound 7
60 µM	-4	8	n.s.	157	37	a	16	7	b	
30 µM	-5	4	n.s.	86	20	a	14	5	b	
15 µM	-7	4	n.s.	53	8	a	3	10	n.s.	
5 µM	-3	5	n.s.	73	35	b	10	9	n.s.	

S.D. = Standard Deviation
α = Confidence Level

a = $p < 0{,}01$
b = $0{,}01 < p < 0{,}05$
n.s. = $p > 0{,}05$ (not significant)

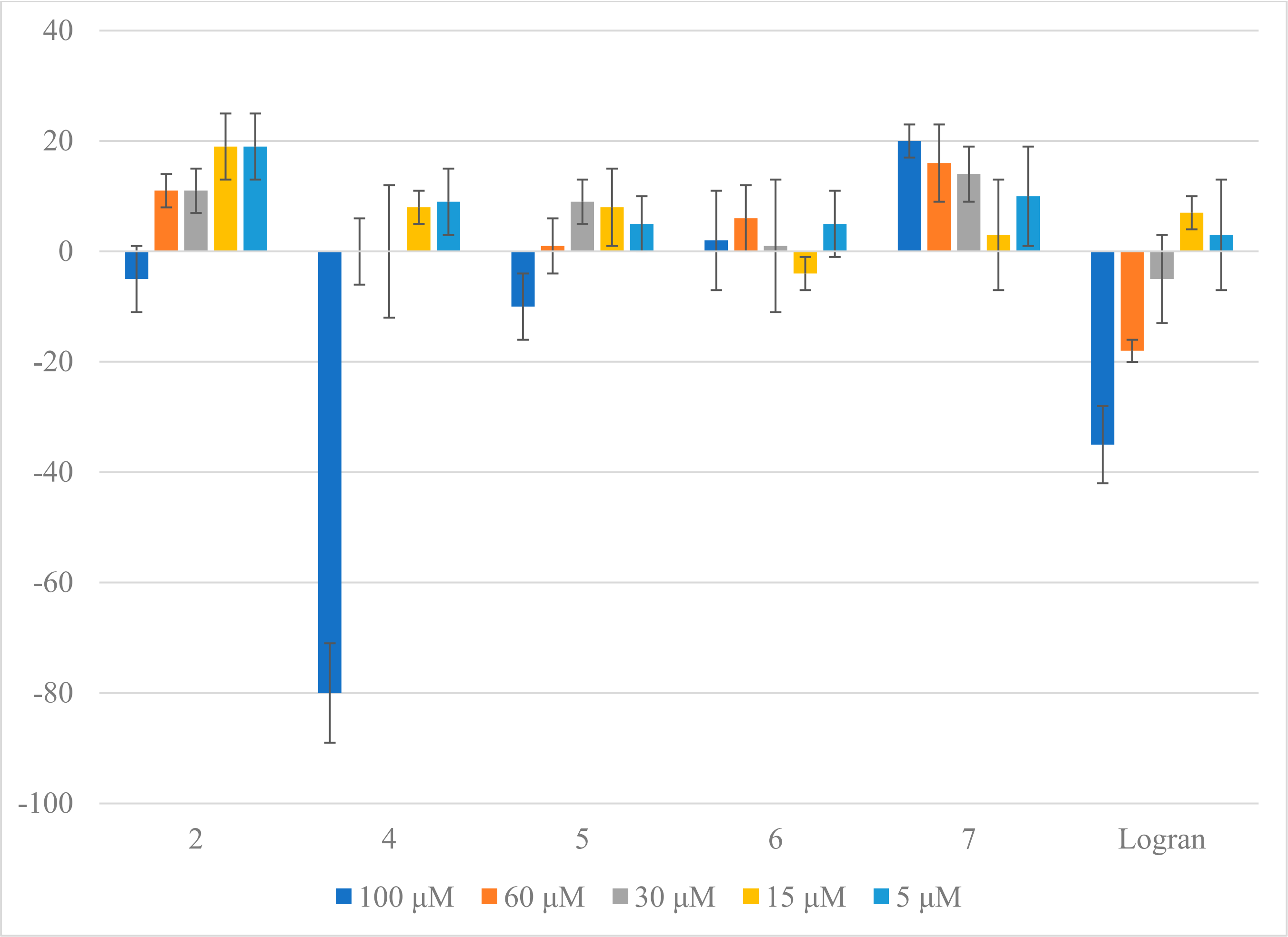

Figure S35: Effect of compounds 2 and 4-7 on the stalk length of *Lepidium sativum* (garden cress). Shoot length of seedlings from *L. sativum* (garden cress) seeds exposed to aqueous 0.1% (v/v) DMSO solutions of compounds at different concentrations. Controls consisted of deionized water with the same concentration of DMSO. Values are expressed as percentage difference from the negative control: shoot length (%) = [(length – length of negative control)/length of negative control]x100. Error bars represent the standard deviation.

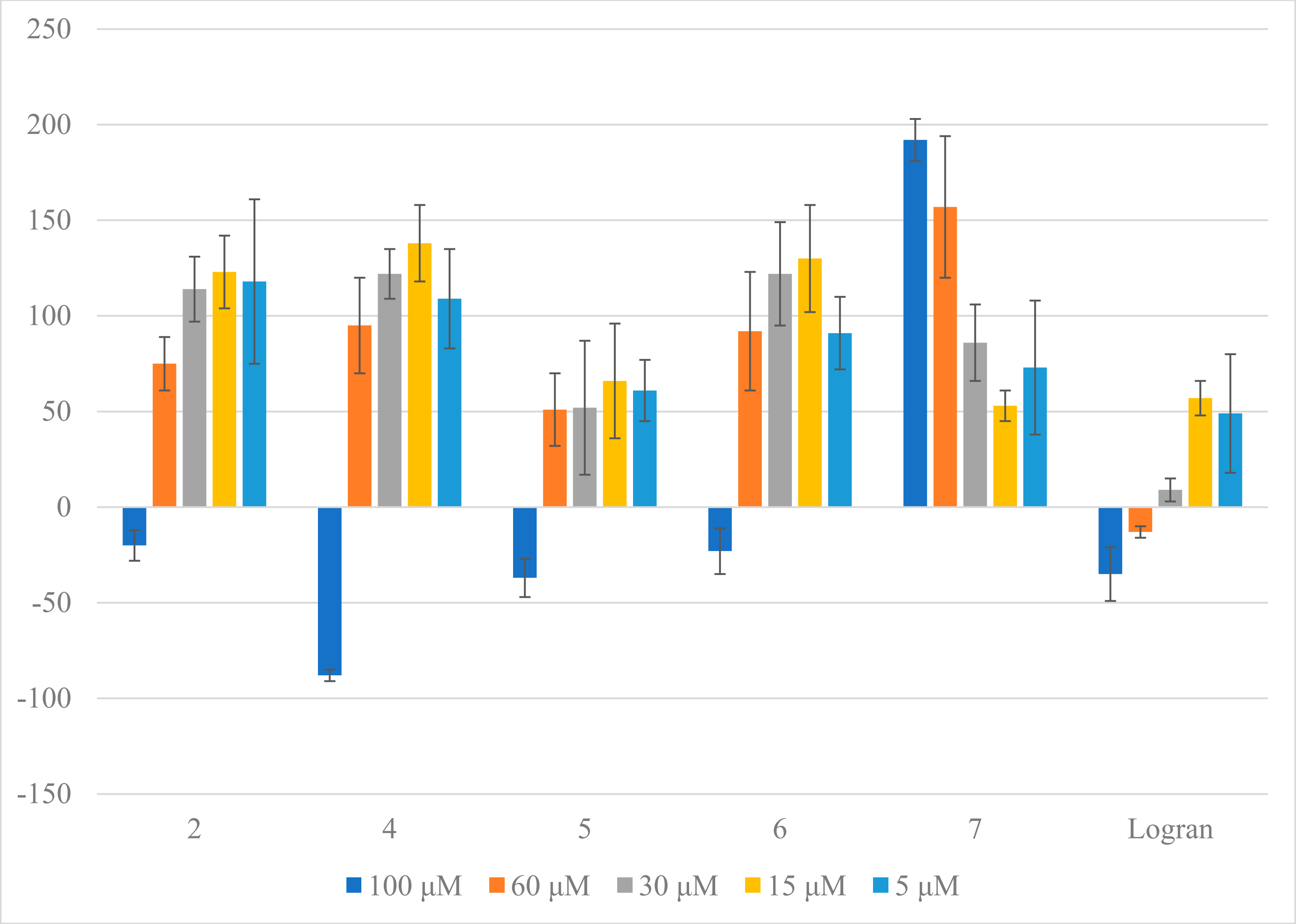

Figure S36: Effect of compounds **2** and **4-7** on the radicle length of *Lepidium sativum*. Radicle length of seedlings from *L. sativum* (garden cress) seeds exposed to aqueous 0.1% (v/v) DMSO solutions of compounds at different concentrations. Controls consisted of deionized water with the same concentration of DMSO. Values are expressed as percentage difference from the negative control: shoot length (%) = [(length – length of negative control)/length of negative control]x100. Error bars represent the standard deviation.

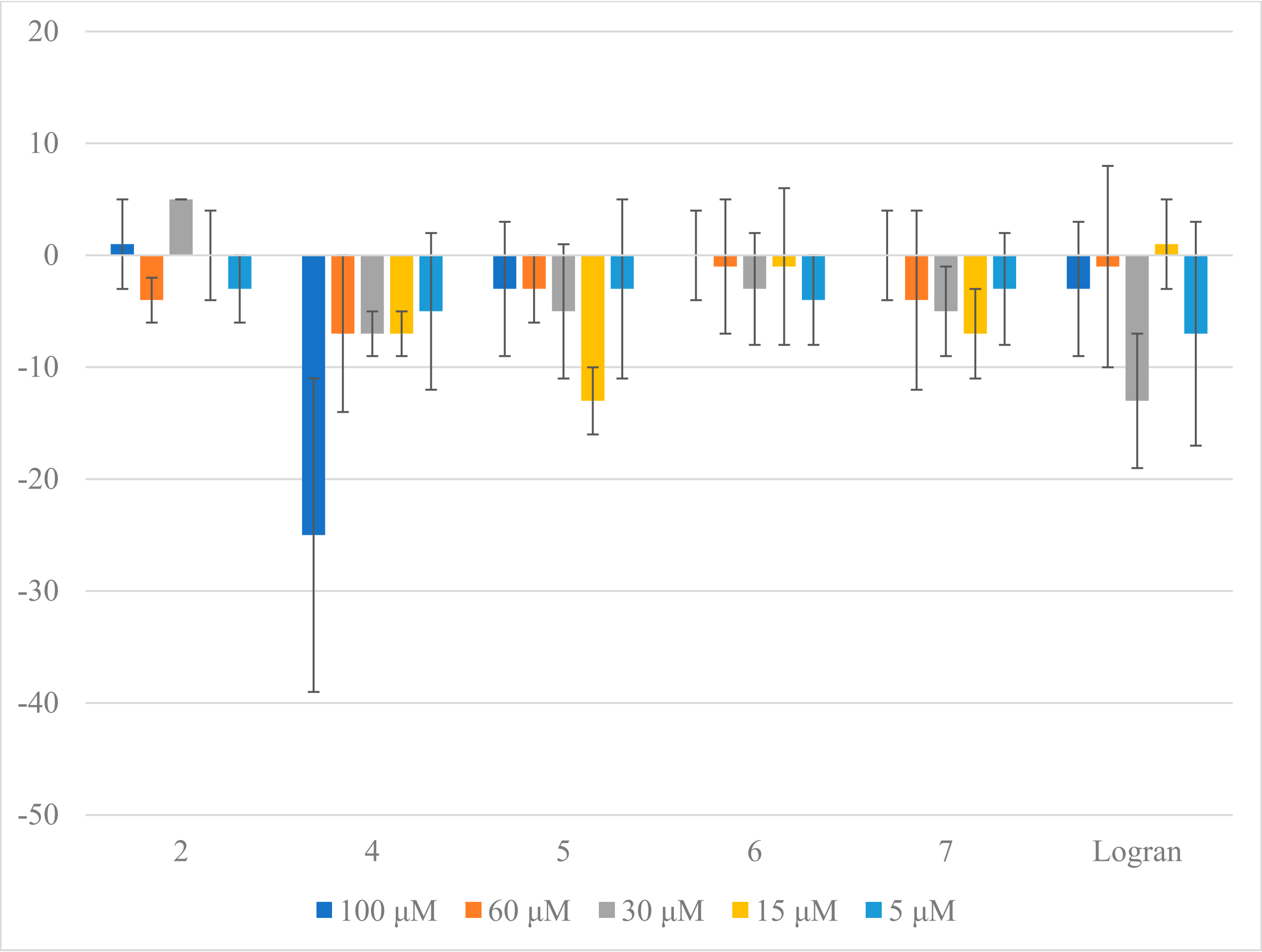

Figure S37: Effect of compounds **2** and **4-7** on the germination of *Lepidium sativum* (garden cress). *L. sativum* (garden cress) seeds were exposed to aqueous 0.1% (v/v) DMSO solutions of compounds at different concentrations. Controls consisted of deionized water with the same concentration of DMSO. Values are expressed as percentage difference from the negative control: shoot length (%) = [(length – length of negative control)/length of negative control]x100. Error bars represent the standard deviation.

Table S11: Results of bioassays on *Lactuca sativa* (lettuce) seed germination.

	BIOACTIVITY									
	GERMINATION			ROOT			SHOOT			
	% from control	S. D.	α	% from control	S. D.	α	% from control	S. D.	α	
100 μM	-9	6	n.s.	-66	3	a	-34	2	a	Reference Compound
60 μM	0	2	n.s.	-64	3	a	-26	6	a	
30 μM	-9	6	n.s.	-54	6	a	-5	4	n.s.	
15 μM	-5	9	n.s.	-22	11	b	-7	17	n.s.	
5 μM	-8	8	n.s.	-19	4	a	0	3	n.s.	
100 μM	-38	14	b	-21	13	n.s.	18	6	b	Compound 2
60 μM	0	2	n.s.	14	16	n.s.	5	4	n.s.	
30 μM	3	2	n.s.	12	6	n.s.	-1	4	n.s.	
15 μM	-1	4	n.s.	20	11	b	5	2	b	
5 μM	-4	6	n.s.	12	9	n.s.	-1	3	n.s.	
100 μM	-12	5	b	-18	5	b	9	2	a	Compound 4
60 μM	-1	0	n.s.	36	6	a	3	4	n.s.	
30 μM	-1	4	n.s.	36	12	a	2	6	n.s.	
15 μM	-1	4	n.s.	29	5	a	-10	12	n.s.	
5 μM	-1	5	n.s.	24	7	a	0	2	n.s.	
100 μM	-25	17	n.s.	-28	7	a	2	4	n.s.	Compound 5
60 μM	-3	6	n.s.	8	10	n.s.	3	4	n.s.	
30 μM	0	4	n.s.	-4	5	n.s.	1	3	n.s.	
15 μM	-1	4	n.s.	7	10	n.s.	-3	4	n.s.	
5 μM	-4	6	n.s.	12	7	n.s.	-7	1	a	
100 μM	-5	9	n.s.	8	8	n.s.	11	4	a	Compound 6
60 μM	-1	4	n.s.	33	15	b	3	5	n.s.	
30 μM	-14	11	n.s.	30	13	b	-3	7	n.s.	
15 μM	-5	8	n.s.	31	9	a	-5	3	b	
5 μM	-5	6	n.s.	15	6	b	-2	6	n.s.	
100 μM	-32	13	b	34	12	a	4	8	n.s.	Compound 7
60 μM	0	7	n.s.	29	14	b	-3	4	n.s.	
30 μM	1	3	n.s.	7	7	n.s.	-9	2	a	
15 μM	-6	7	n.s.	-1	15	n.s.	-4	7	n.s.	
5 μM	-3	6	n.s.	-2	11	n.s.	-2	3	n.s.	

S.D. = Standard Deviation
α = Confidence Level

a = $p < 0,01$
b = $0,01 < p < 0,05$
n.s. = $p > 0,05$ (not significant)

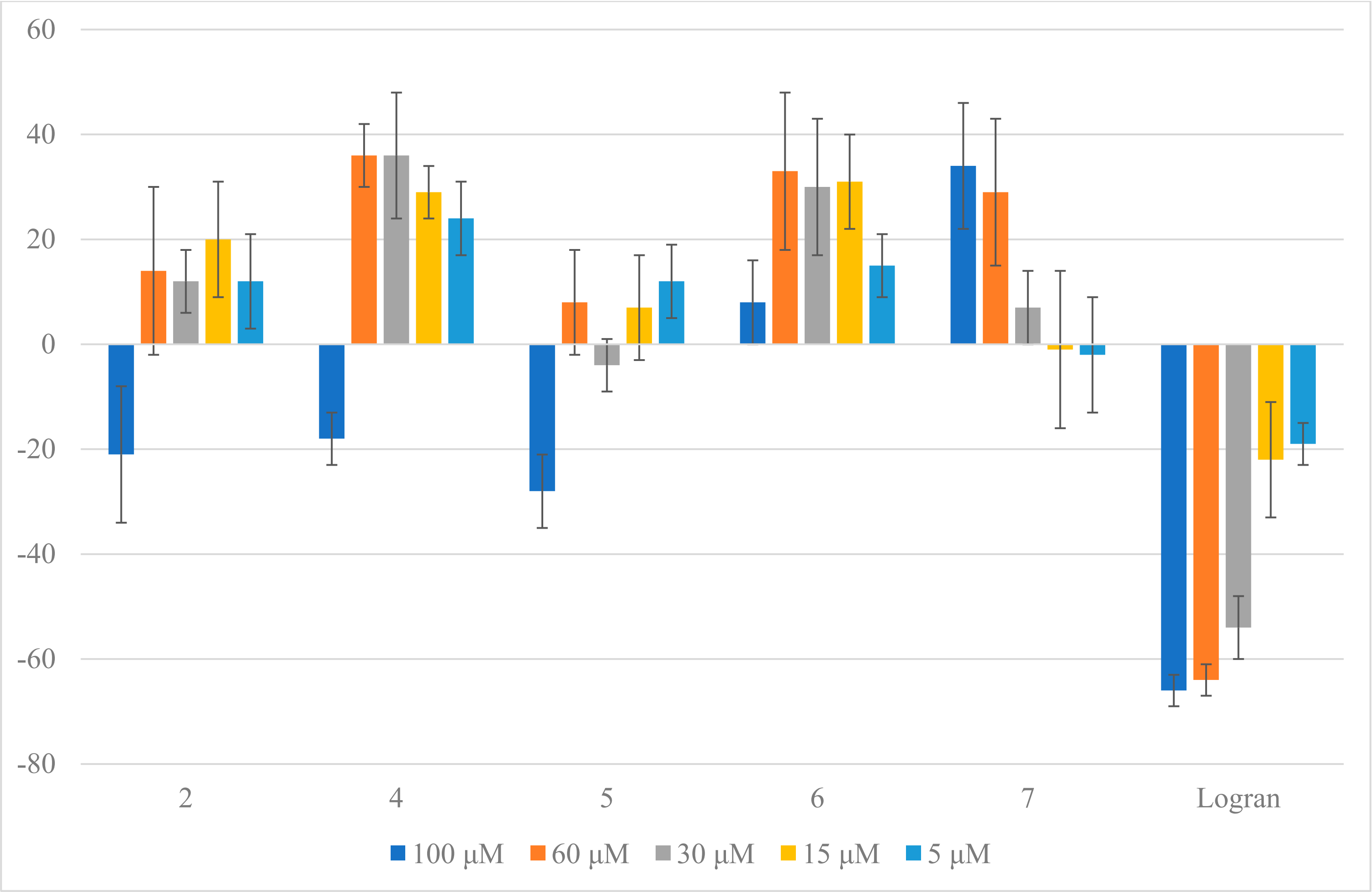

Figure S38: Effect of compounds **2** and **4-7** on the radicle length of *L. sativa*. Radicle length of seedlings from *L. sativa* (lettuce) seeds exposed to aqueous 0.1% (v/v) DMSO solutions of compounds at different concentrations. Controls consisted of deionized water with the same concentration of DMSO. Values are expressed as percentage difference from the negative control: shoot length (%) = [(length – length of negative control)/length of negative control]x100. Error bars represent the standard deviation.

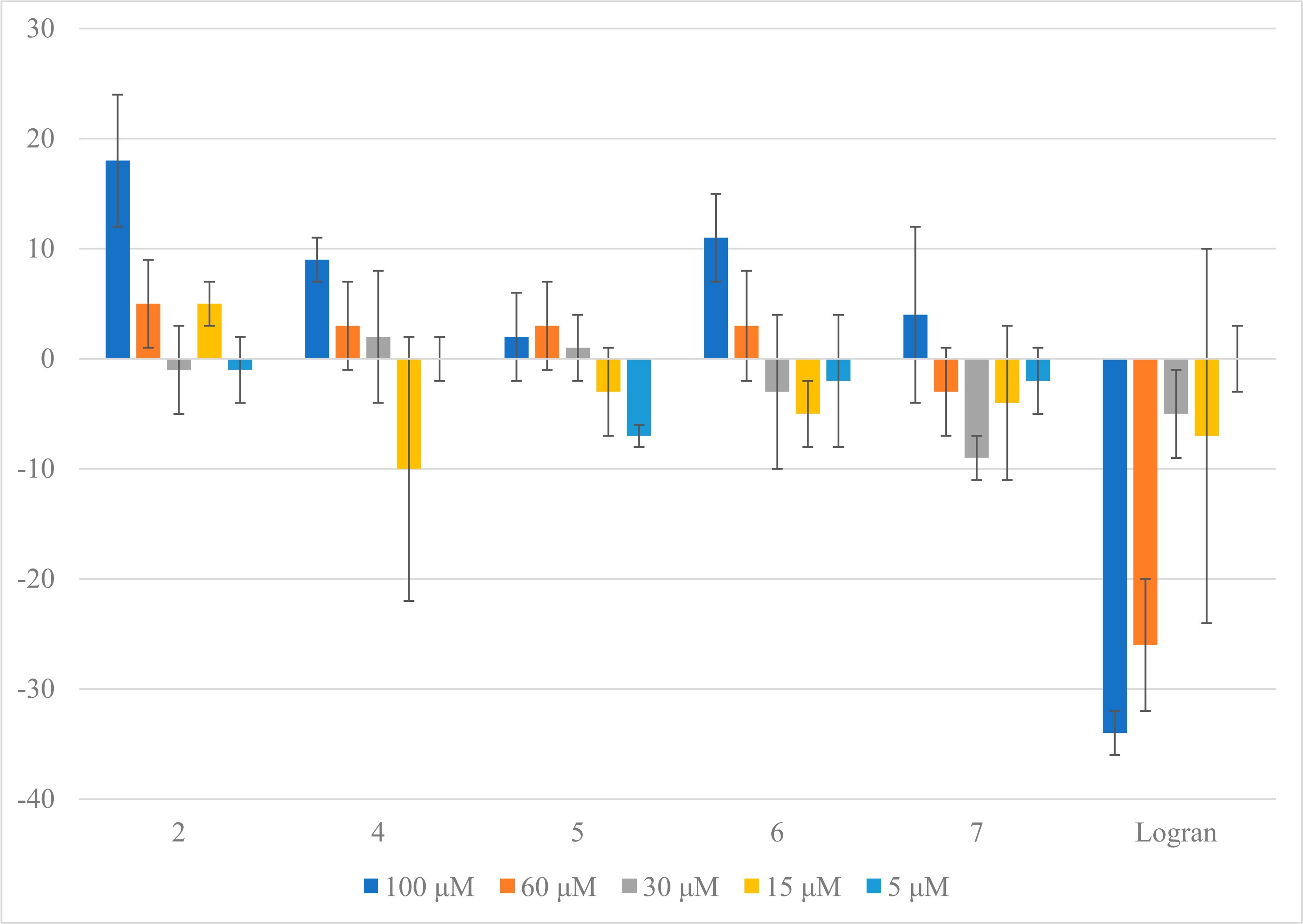

Figure S39: Effect of compounds **2** and **4-7** on the stalk length of *Lactuca sativa* (lettuce). Shoot length of seedlings from *L. sativa* (lettuce) seeds exposed to aqueous 0.1% (v/v) DMSO solutions of compounds at different concentrations. Controls consisted of deionized water with the same concentration of DMSO. Values are expressed as percentage difference from the negative control: shoot length (%) = [(length – length of negative control)/length of negative control]x100. Error bars represent the standard deviation.

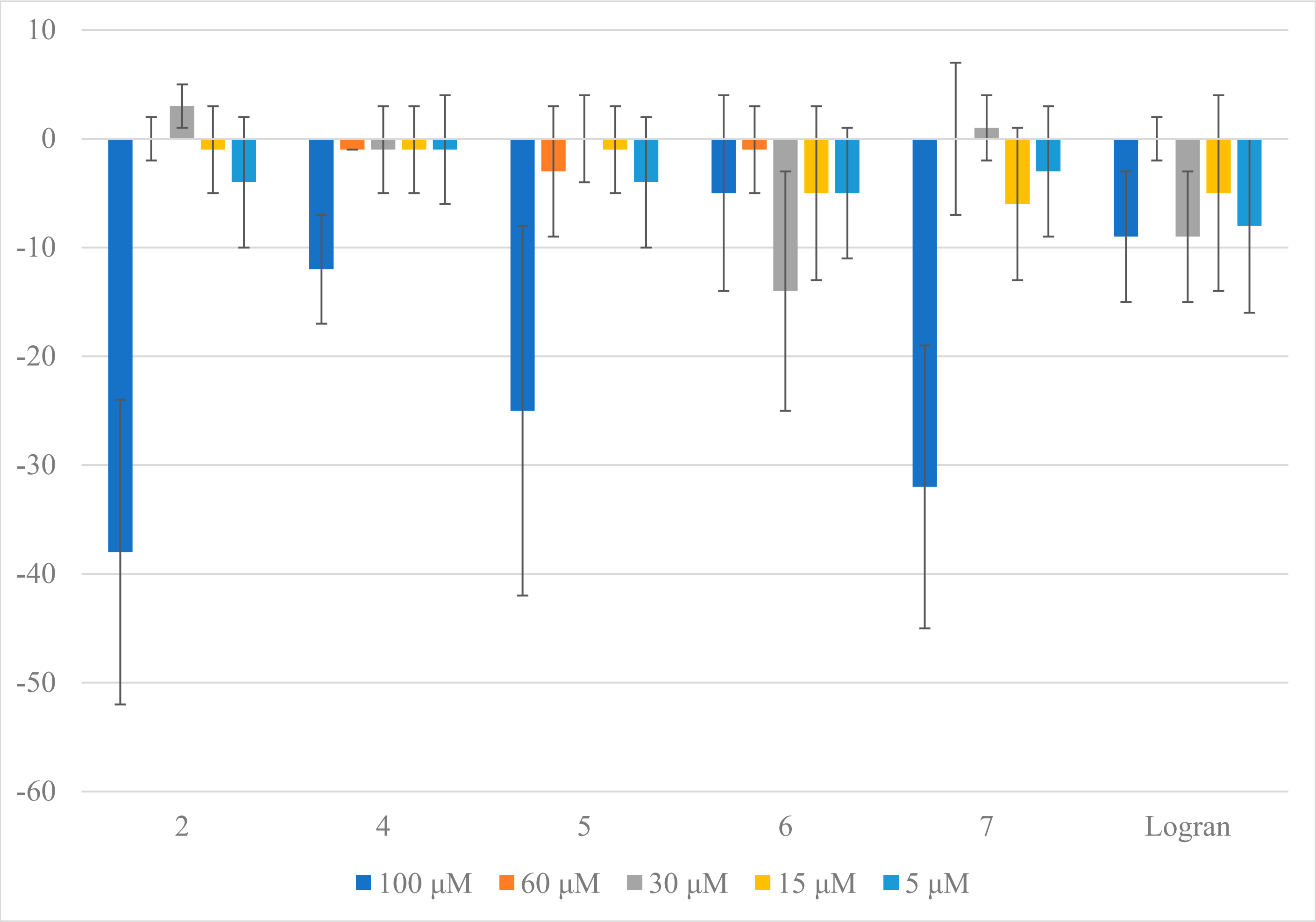

Figure S40: Effect of compounds **2** and **4-7** on the germination of *Lactuca sativa* (lettuce). *L. sativa* (lettuce) seeds were exposed to aqueous 0.1% (v/v) DMSO solutions of compounds at different concentrations. Controls consisted of deionized water with the same concentration of DMSO. Values are expressed as percentage difference from the negative control: shoot length (%) = [(length – length of negative control)/length of negative control]x100. Error bars represent the standard deviation.
